# Nucleoside diphosphate kinases 1 and 2 regulate a protective liver response to a high-fat diet

**DOI:** 10.1126/sciadv.adh0140

**Published:** 2023-09-06

**Authors:** Domenico Iuso, Isabel Garcia-Saez, Yohann Couté, Yoshiki Yamaryo-Botté, Elisabetta Boeri Erba, Annie Adrait, Nour Zeaiter, Malgorzata Tokarska-Schlattner, Zuzana Macek Jilkova, Fayçal Boussouar, Sophie Barral, Luca Signor, Karine Couturier, Azadeh Hajmirza, Florent Chuffart, Ekaterina Bourova-Flin, Anne-Laure Vitte, Lisa Bargier, Denis Puthier, Thomas Decaens, Sophie Rousseaux, Cyrille Botté, Uwe Schlattner, Carlo Petosa, Saadi Khochbin

**Affiliations:** ^1^ Univ. Grenoble Alpes, CNRS UMR 5309, INSERM U1209, Institute for Advanced Biosciences, La Tronche 38706, France.; ^2^ Univ. Grenoble Alpes, CNRS, CEA, Institut de Biologie Structurale (IBS), Grenoble 38000, France.; ^3^ Univ. Grenoble Alpes, INSERM, CEA, UMR BioSanté U1292, CNRS, CEA, FR2048, Grenoble 38000, France.; ^4^ Univ. Grenoble Alpes, INSERM, Laboratory of Fundamental and Applied Bioenergetics, Grenoble, France.; ^5^ CHU Grenoble Alpes, Service d’hépato-gastroentérologie, Pôle Digidune, La Tronche 38700, France.; ^6^ Aix Marseille Université, INSERM, TAGC, TGML, Marseille 13288, France.; ^7^ Univ. Grenoble Alpes, INSERM, Institut Universitaire de France, Laboratory of Fundamental and Applied Bioenergetics, Grenoble, France.

## Abstract

The synthesis of fatty acids from acetyl–coenzyme A (AcCoA) is deregulated in diverse pathologies, including cancer. Here, we report that fatty acid accumulation is negatively regulated by nucleoside diphosphate kinases 1 and 2 (NME1/2), housekeeping enzymes involved in nucleotide homeostasis that were recently found to bind CoA. We show that NME1 additionally binds AcCoA and that ligand recognition involves a unique binding mode dependent on the CoA/AcCoA 3′ phosphate. We report that *Nme2* knockout mice fed a high-fat diet (HFD) exhibit excessive triglyceride synthesis and liver steatosis. In liver cells, NME2 mediates a gene transcriptional response to HFD leading to the repression of fatty acid accumulation and activation of a protective gene expression program via targeted histone acetylation. Our findings implicate NME1/2 in the epigenetic regulation of a protective liver response to HFD and suggest a potential role in controlling AcCoA usage between the competing paths of histone acetylation and fatty acid synthesis.

## Teaser

NME1/2 mediate transcriptional reprogramming of liver cells in response to a high-fat diet.

## INTRODUCTION

**Fig. 1.**
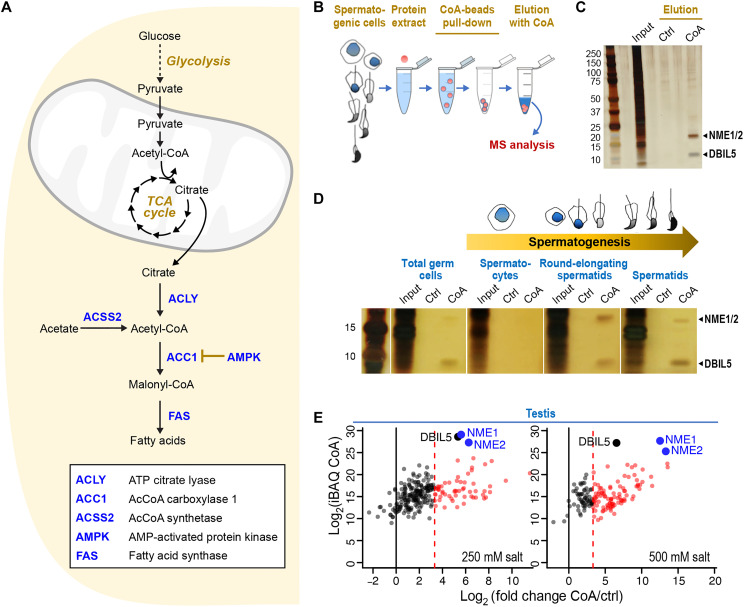
NME1/2 are major CoA-binding factors in mouse spermatogenic cells. (**A**) Overview of DNL. Under conditions of carbohydrate excess, mitochondrial citrate produced by the tricarboxylic acid (TCA) cycle is exported to the cytosol and converted to AcCoA by ACLY. Acetate is converted to AcCoA by ACSS2 (*65*). The commitment step of DNL catalyzed by ACC1 is negatively regulated by AMPK. (**B**) Strategy for purifying CoA-binding factors present in extracts from total testis or fractionated spermatogenic cells. After the incubation of extracts with CoA-coated Sepharose beads and successive washes, bound proteins were eluted with either free CoA or water as a control. (**C**) Silver-stained electrophoresis gel of proteins eluted from CoA beads with either water (Ctrl) or CoA. The major proteins captured by CoA are indicated. (**D**) Total testis extracts or equivalent amounts of extracts from the indicated fractionated spermatogenic cells were incubated with CoA beads and processed as in (B). (**E**) The identities and abundances of proteins eluted by free CoA (CoA) and water (Ctrl) were deduced from MS-based proteomic analyses. The graphs display the log_2_(iBAQ) of the proteins in CoA (representative of the relative abundance of the different proteins identified in this sample) on the *y* axis plotted against the log_2_(fold change CoA/ctrl) on the *x* axis (representative of the differential abundance between CoA and ctrl fishing). For each representation, the black and dashed red vertical lines indicate, respectively, log_2_(fold change) = 0 and log_2_(fold change) = 3.32 [i.e., fold change (CoA/ctrl) = 10]. Black and red dots highlight proteins with a fold change (CoA/ctrl) < 10 and ≥ 10, respectively. The three most abundant proteins, NME1/2 and DBIL5, are indicated. CoA pull-downs were performed on total testis extracts in the presence of either 250 mM (left) or 500 mM (right) KCl.

De novo lipogenesis (DNL) is the pathway used primarily by the liver and adipose tissue to synthesize fatty acids from excess carbohydrates or other precursors (*1*). Deregulated DNL, especially in the liver, is implicated in diverse pathologies (*2*). Increased rates of DNL are associated with nonalcoholic fatty liver disease (*3*, *4*), insulin resistance and type 2 diabetes (*4*, *5*), cardiovascular disease (*6*), incident heart failure (*7*), and cancer (*8*). The main carbon source for DNL is the cytoplasmic pool of acetyl–coenzyme A (AcCoA), which is derived from either citrate or acetate through the activities of adenosine triphosphate (ATP) citrate lyase (ACLY) and AcCoA synthetase (ACSS2), respectively (Fig. 1A) (*9*, *10*). AcCoA is irreversibly converted to malonyl-CoA by the action of AcCoA carboxylases 1 and 2 (ACC1 and ACC2), which localize to the cytosol and outer mitochondrial membrane, respectively. While the malonyl-CoA produced by ACC2 negatively regulates fatty acid import into mitochondria and hence β-oxidation, that produced by ACC1 is used in iterative cycles of fatty acid elongation by fatty acid synthase (FAS) to generate fatty acids, predominantly the C16:0 fatty acid, palmitate.

ACC1 catalyzes the first committed step of DNL and hence is subject to tight regulation. Besides allosteric activation by citrate and product inhibition by malonyl- and palmitoyl-CoA, ACC1 is also tightly regulated by the cellular energy level. Energy deficiency, characterized by a depletion of ATP and an accumulation of adenosine diphosphate (ADP) and adenosine monophosphate (AMP), triggers the inactivating phosphorylation of ACC1 by AMP-activated protein kinase (AMPK), thereby inhibiting DNL. High levels of ATP, in contrast, favor the massive use of AcCoA in fatty acid synthesis. This well-characterized mechanism establishes a tight relationship between ATP levels, ACC1 activity, and fatty acid synthesis and storage (*11*). Here, we report the discovery that fatty acid accumulation is controlled by an additional layer of regulation, in which the key players are the nucleoside diphosphate kinases (NDPKs) 1 and 2 (NME1 and NME2).

NDPKs mediate a large variety of cellular functions that include nucleotide homeostasis, endocytosis, intracellular trafficking, cell motility, and DNA repair, with implications in cancer and metastatic cancer development (*12*). In mammals, the most abundant and best-studied NDPKs are NME1 and NME2 (hereafter abbreviated NME1/2), which are closely related isoforms (88% sequence identity) with similar functional attributes and localization in the cytosol and nucleus. The best-characterized activity of these enzymes is the phosphorylation of NDPs, using nucleoside triphosphates (NTPs), primarily ATP, as a donor. This reaction, which plays a key role in equilibrating the nucleotide pools in the cell, proceeds by a ping-pong mechanism involving the formation of a phosphohistidine intermediate in the catalytic site. NME1/2 can also transfer the phosphate group from this intermediate to a histidine in unrelated substrate proteins and accordingly are also described as histidine kinases (*13*).

Recently, NME1/2 were reported to have the remarkable and unexpected ability to bind CoA (*14*). CoA binds noncovalently to NME1 and competitively inhibits NDPK activity. Moreover, in cells under oxidative or metabolic stress, NME1 undergoes CoAlation (covalent modification via disulfide formation with the CoA thiol group) at a specific cysteine near the enzyme’s active site, resulting in the stable inhibition of NDPK activity (*14*). A more recent study reported that NME1/2 also interact with long-chain fatty acyl (LCFA)–CoA, which binds via its nucleotide moiety to the enzyme’s active site, as revealed by the crystal structure of NME2 bound to myristoyl-CoA (*15*). The study also showed that the increased production of cellular LCFA-CoA inhibited clathrin-mediated endocytosis, an NME1/2-dependent process, and that LCFA-CoA compromised the metastasis suppressor function of NME1 in mouse models of breast cancer under high-fat diet (HFD) conditions (*15*). Together, these studies suggest that CoA and its derivatives may regulate diverse NME1/2-mediated processes.

Here, we report a previously undescribed role for NME1/2 in the control of fatty acid accumulation as well as in a protective liver cell response to an HFD challenge. We present in vitro and structural data showing that, in addition to CoA and LCFA-CoA, NME1 also binds AcCoA, the most abundant CoA derivative, and that ligand recognition occurs via a unique nucleotide-binding mode critically dependent on the CoA 3′ phosphate group. Moreover, binding is inhibited by ATP-induced histidine phosphorylation and hence potentially sensitive to the cellular energy status. Focusing on a mouse knockout (ko) model with drastically reduced NME1/2 levels, we show that the major cytoplasmic AcCoA–consuming pathway is stimulated during liver fatty acid synthesis, leading to increased liver triglyceride levels and liver steatosis. Under HFD challenge, NME1/2 exerts an inhibitory role on fatty acid accumulation, specifically repressing genes encoding key transcription factors involved in lipogenesis and fatty acid metabolism. At the same time, NME1/2 mediate an increased targeted histone H3K9 acetylation, activating a gene signature known to protect liver cells during regeneration. These observations identify NME1/2 as a critical regulator of the competing processes of histone acetylation and fatty acid synthesis, placing NME1/2 among a select group of metabolic enzymes with a moonlighting function in epigenetic regulation (*16*).

## RESULTS

### NME1/2 are major CoA-binding factors in mouse spermatogenic cells

NME1/2 were previously identified as CoA- or LCFA-CoA–interacting proteins in human embryonic kidney 293 cell lines and rat heart extracts (*14*, *15*). We independently found that NME1/2 bind CoA while investigating the global genome reprogramming that occurs in mouse spermatogenic cells during postmeiotic (spermatid) maturation, which is characterized by a switch from a nucleosome- to a protamine-based organization following the nearly genome-wide eviction of histones. Surmising that the genome-wide increase in histone acetylation observed before histone eviction (*17*–*19*) requires an enhanced production, management, and use of AcCoA, we sought to identify CoA-binding regulatory factors involved in managing the AcCoA pool in spermatids. We incubated CoA-coated beads with extracts from mouse testis and spermatogenic cells enriched at different stages of their development by fractionation and subsequently eluted CoA-binding proteins with free CoA (Fig. 1B). Mass spectrometry (MS)–based proteomic analysis identified several CoA-associated proteins common to all samples, including total testis extracts pulled down with different stringencies as well as extracts from fractionated spermatogenic cells (table S1). In addition to the known testis-specific acyl-CoA–binding protein DBIL5 (*20*), the most prominent CoA-binding factors were NME1/2 (Fig. 1, C to E; fig. S1; and table S1). This is consistent with the strong prevalence of NME1/2 detected among CoA-binding factors in diverse somatic rat tissues (*14*), highlighting the ubiquitous nature of NME1/2’s CoA-binding functionality.

### NME1 binds AcCoA in vitro

**Fig. 2.**
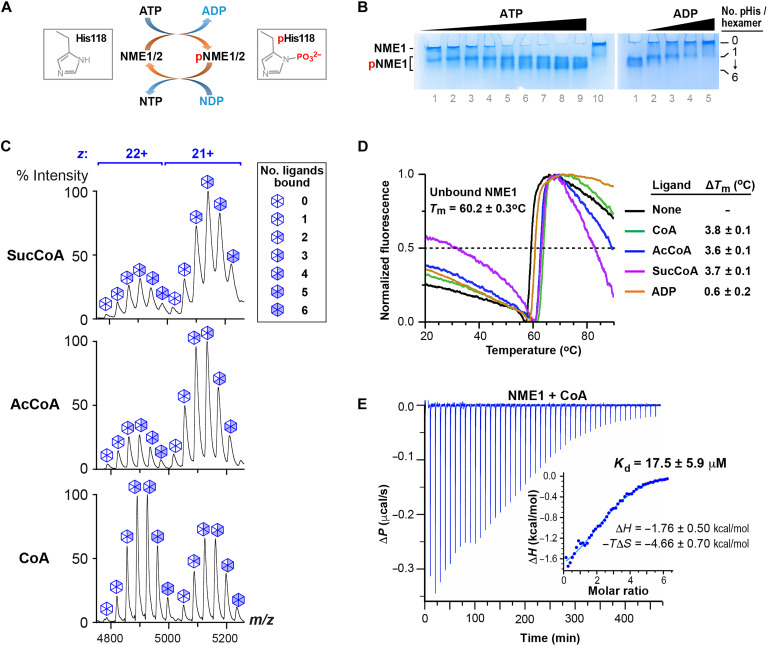
NME1 binds short-chain acyl-CoA ligands in vitro. (**A**) Scheme illustrating phosphohistidine-mediated NDPK activity. Only one direction of the fully reversible reaction is shown. (**B**) Gel shift experiments confirm NDPK activity of purified recombinant NME1. Left gel: NME1 is phosphorylated by ATP. Coomassie-stained native polyacrylamide gel of bacterially expressed NME1 incubated without (lane 10) or with (lanes 1 to 9) increasing amounts of ATP. At higher ATP concentrations, more subunits within each NME1 hexamer are phosphorylated, resulting in additional higher mobility bands on the gel. Right gel: NME1 is dephosphorylated by ADP. Native gel of phosphorylated NME1 incubated without (lane 1) or with (lanes 2 to 5) increasing amounts of ADP, illustrating phosphoryl group transfer from NME1 to ADP. (**C**) Native MS analysis of recombinant purified NME1 incubated with CoA, AcCoA, or SucCoA, as indicated. Peaks corresponding to charge states +22 and +21 represent the NME1 hexamer with 0 to 6 ligands bound. (**D**) Thermal denaturation profile of NME1 measured by nanoDSF. Representative profiles are shown for NME1 in the absence and presence of CoA ligands. *T*_m_ and Δ*T*_m_ values represent the means and SD from three independent experiments. (**E**) Representative ITC profile of CoA binding by NME1. Differential power (Δ*P*) data time course of raw injection heats for a titration of CoA into NME1. Inset: Normalized binding enthalpies corrected for heat of dilution as a function of binding site saturation. Data were fit using a single-site binding model. *K*_d_ and thermodynamic parameters represent means and SD values determined from three independent experiments.

Since NME1/2 recognizes myristoyl-CoA exclusively by its nucleotide moiety (*15*), NME1/2 should also bind AcCoA and other short-chain acyl-CoA molecules such as succinyl-CoA (SucCoA). To verify this, we assessed the ability of purified NME1 to bind these molecules in vitro. NME1/2 are hexameric enzymes that catalyze the reaction XTP + YDP ⇆ XDP + YTP by first transferring the γ-phosphoryl group from XTP (usually ATP) to residue His^118^, and then from the phosphohistidine to YDP, where Y is any of the four common (deoxy)nucleosides (Fig. 2A). We confirmed that recombinant murine NME1 purified from bacteria could be phosphorylated by ATP and subsequently dephosphorylated by ADP or guanosine diphosphate (GDP) (Fig. 2B and fig. S2, A and B). NME1 incubated with CoA was then analyzed by native MS, which allows the mass measurement of intact noncovalent complexes (*21*). NME1 hexamers were observed in the unbound state and bound to a variable number of CoA molecules ranging from one to six (Fig. 2C). The observed pattern of peak intensities matched the distribution of ligand-bound states expected for a hexamer with six independent binding sites that are each approximately half occupied (fig. S3). Incubating NME1 with either AcCoA or SucCoA yielded similar spectra, revealing that NME1 does not strongly discriminate between these ligands and CoA.

To further evaluate the relative affinity of NME1 for CoA, AcCoA, and SucCoA, we used nano-differential scanning fluorimetry (nanoDSF) to assess ligand-induced thermal stabilization. The recorded profiles revealed that the melting temperature of NME1 (*T*_m_ = 60°C) was enhanced similarly by each of the tested ligands (Δ*T*_m_ ≈ 3.7°C), confirming that AcCoA and SucCoA bind NME1 with similar affinity to CoA (Fig. 2D). Since the latter affinity has not been previously quantified, we analyzed the NME1:CoA interaction by isothermal titration calorimetry (ITC). This revealed a dissociation constant (*K*_d_) of ~18 μM (Fig. 2E), comparable to or lower than the *K*_d_ values of 25 to 120 μM reported for the binding of NDPKs to ADP (*22*–*25*). Consistent with this observation, ADP was less effective than the CoA ligands at stabilizing NME1 in the nanoDSF assay (Δ*T*_m_ = 0.6°C, Fig. 2D). Together, these data indicate that CoA, AcCoA, and SucCoA have similar affinity for NME1 and bind at least as well, if not better, than canonical NDP substrates.

### NME1 recognizes CoA via a unique nucleotide-binding mode

**Fig. 3.**
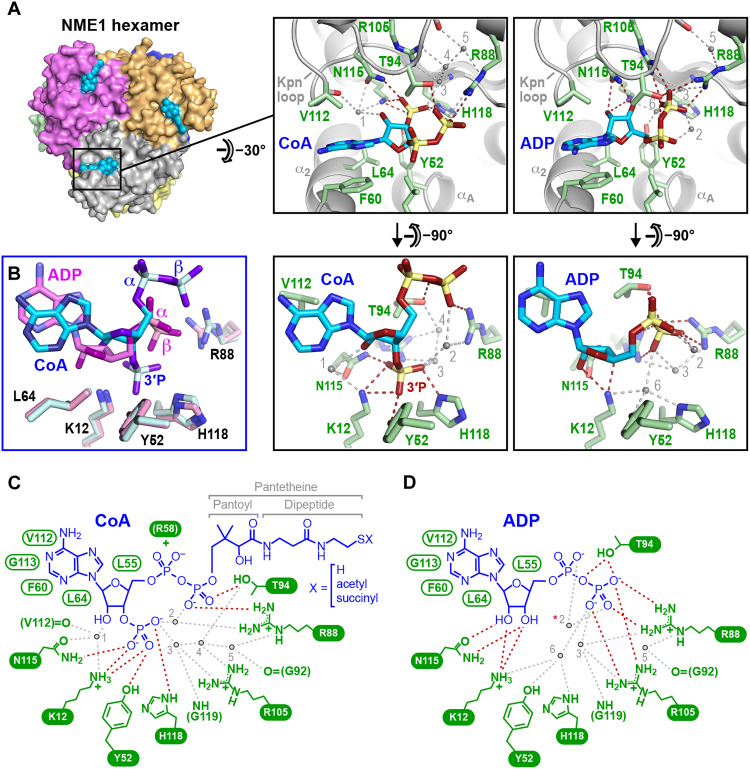
NME1 recognizes CoA via a unique binding mode dependent on the 3′ phosphate. (**A**) Structural comparison of NME1 bound to CoA/SucCoA (left) and ADP (right). Direct and water-mediated hydrogen bonds are shown as red and gray dashed lines, respectively. (**B**) Superimposition of the CoA and ADP ligands showing rotation of the adenine base and repositioning of the α- and β-phosphate groups, relatively shifted by 2.9 and 3.9 Å, respectively. (**C** and **D**) Schematic summary of interactions for (C) CoA/SucCoA and (D) ADP. Red and gray dashed lines represent direct and water-mediated hydrogen bonds. NME1 residues mediating H bonding are indicated in white on a green background; residues mediating van der Waals or aromatic stacking interactions are labeled green. Arg^58^ forms a salt bridge with the CoA α phosphate in a subset of NME1 subunits. Important water molecules are numbered 1 to 6. Three of these are conserved between the ADP- and SucCoA-bound structures. In previous ADP-bound NDPK structures (PDB 1NUE and 1NDP), a Mg^2+^ ion replaces water molecule 2 (red asterisk). The CoA 3′ phosphate group forms direct H bonds with Lys^12^, Tyr^52^, Asn^115^, and His^118^ as well as water-mediated H bonds with Arg^88^, Arg^105^, and the Gly^119^ backbone. In the ADP-bound structure, these residues either hydrogen bond with the ribose 2′ and 3’ OH groups or with a water molecule H-bonded to the β phosphate. Because the CoA ribose is displaced away from the protein by the 3′ phosphate, the H bonds that NME1 residues Lys^12^ and Asn^115^ make with the 2’ OH group of ADP are replaced by water-mediated bonds that allow the larger protein-ligand distance to be bridged. Similarly, the outward shift of the CoA β phosphate is accommodated by water molecules that satisfy the H bonding potential of residues Arg^88^ and Arg^105^, which, in the ADP-bound structure, directly contact the β-phosphate.

Efforts to cocrystallize murine NME1 with the above ligands led to high-resolution (1.96 and 2.2 Å, respectively) crystal structures of NME1 bound to SucCoA and ADP, and a somewhat lower (2.6 Å)–resolution structure bound to CoA (table S2). As expected, the structure with ADP closely matches that of the ADP-bound human ortholog (*26*), while those with CoA and SucCoA are nearly identical to each other and closely resemble that of human NME2 bound to myristoyl-CoA (table S3 and fig. S4, A and B) (*15*). Briefly, each NME1 subunit within the D_3_-symmetric hexamer comprises an antiparallel four-stranded β sheet flanked on either side by a layer of helices (*27*). ADP is sandwiched between the α_A_-α_2_ helical hairpin and an α3-β4 loop segment called the “Kpn loop” that define the enzyme’s active site (Fig. 3A). CoA and SucCoA bind to this site via their common nucleotide moiety, the only part of these ligands specifically recognized by NME1. (Hence, the CoA and SucCoA ligands in our structures are hereafter collectively called “CoA” unless otherwise specified.) In the electron density maps, no or only poorly defined density is observed for the pantetheine and succinyl moieties of these ligands (fig. S5, compare A and B), except in a few NME1 subunits where they make nonspecific contacts with nearby residues. Consequently, the acyl-bearing end of CoA remains solvent accessible when bound to NME1 and potentially free to interact with other protein partners, consistent with our findings that NME1 binds similarly to acylated and non-acylated CoA (Fig. 2, C and D).

How NME1/2 recognizes the CoA nucleotide in atomic detail has not previously been described and hence is presented here. Unexpectedly, NME1 recognizes ADP and CoA via completely distinct binding modes (Fig. 3, A to D). Of the 10 direct hydrogen bonds mediating the NME1:ADP interaction, only two are conserved in the NME1:CoA complex. This is notable because the ligand-interacting residues of NME1 adopt nearly identical conformations in both complexes and because, apart from its 3′ phosphate group, the CoA nucleotide is otherwise chemically identical to ADP. The CoA 3′-phosphate group sits next to the catalytic His^118^ side chain at the bottom of the ligand binding pocket (Fig. 3, A and B), the same site putatively occupied by the ATP γ phosphate group (fig. S6). As a result, the ribose ring sits 2 Å higher than in the ADP-bound structure, adopting a C_2′_ instead of a C_3′_ *endo* pucker. Compared to ADP, the α and β phosphates of CoA are displaced upward and are solvent accessible, pointing out of the pocket to direct the CoA pantetheine end away from the protein surface. This contrasts with the ADP-bound structure, where the β phosphate is buried deep inside the protein and folds back toward the ribose 3′ OH group, with which it forms an intramolecular H bond critical for catalysis (*28*, *29*). Hence, whereas the β phosphate of ADP is intimately recognized by NME1 via numerous H bonds, that of CoA makes fewer protein contacts (Fig. 3, C and D) and exhibits high thermal mobility (fig. S7A). The α phosphate of CoA is also highly mobile, only contacting NME1 through a salt bridge with residue Arg^58^ in a few hexamer subunits (fig. S7B).

How does NME1 accommodate two very different nucleotide-binding modes? First, as in the ADP complex, the adenine base of CoA is sandwiched between hydrophobic residues from the Kpn loop and α_A_-α_2_ helical hairpin but is rotated by ~40° in the plane of the base to compensate for the shifted ribose ring and maintain a stacking interaction with residue Phe^60^ (Fig. 3, A and B). This rotation is possible because NME1 binds nucleotides without forming H bonds with the base moiety, allowing NME1 to bind substrates regardless of the identity of the base. Second, the high resolution of our NME1/SucCoA crystal structure revealed several water molecules structurally conserved across hexamer subunits that play an important role in compensating for the shift of CoA nucleotide atoms relative to their counterparts in ADP (Fig. 3, C and D). Last, the outward shift of the β phosphate is accompanied by an inward shift of residue Thr^94^ in the α3-β4 loop that allows it to hydrogen bond with the repositioned β phosphate (Fig. 3A and fig. S8A).

To confirm the noncanonical binding mode of CoA, we exploited the shift in Thr^94^ position to design a mutation that would selectively disrupt CoA binding without completely abolishing the interaction with ADP and ATP. In silico modeling suggested that replacing Thr^94^ by an Asp residue should yield an NME1 mutant that could accommodate ADP, but not CoA, in the active site (fig. S8B), thereby disrupting CoA binding while allowing NDP phosphorylation to continue. Accordingly, we generated the T94D mutant and evaluated it for NDPK and CoA-binding activity. Wild-type (WT) and mutant forms of glutathione *S*-transferase (GST)–tagged NME1 could both be phosphorylated by ATP and subsequently dephosphorylated by GDP, confirming that the T94D mutant retained notable NDPK activity, albeit at a reduced level compared to the WT (fig. S8, C, top, and D). In contrast, whereas WT NME1 bound strongly to immobilized CoA, no interaction was detected between the T94D mutant and the CoA beads, revealing a dramatic loss of CoA-binding capacity (fig. S8C, bottom). The result confirms our structural observation that NME1 engages CoA and ADP by distinct interaction modes.

### The 3′ phosphate of CoA is a critical binding epitope

**Fig. 4.**
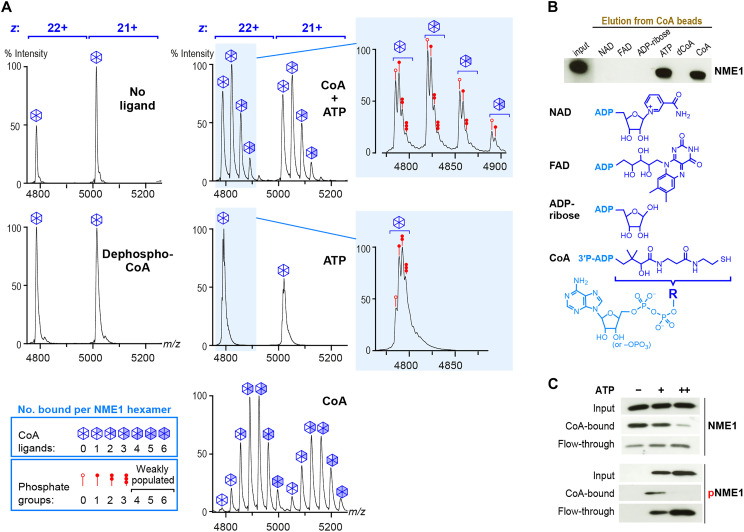
CoA binding by NME1 is mutually exclusive with histidine phosphorylation. (**A**) Native MS analysis of NME1 incubated in the absence of ligand (top left) or the presence of dephospho-CoA (bottom left), CoA (bottom right; same spectrum as in Fig. 2C), ATP (middle right), or an equimolar mixture of CoA and ATP (each in fivefold excess relative to NME1; top right), which leads to a mixture of CoA binding and histidine phosphorylation. Spectra show that the 3′ phosphate of CoA is critical for binding and that CoA binding and phosphorylation of the NME1 hexamer are mutually competitive. (**B**) NME1 does not promiscuously bind common ADP-containing metabolites. NME1-loaded CoA beads (input) were incubated with an excess of the indicated molecules, and the release of NME1 was visualized by an immunoblot of the flow-through probed with an NME1 antibody. dCoA is dephospho-CoA. (**C**) NME1 (3 μg) loaded on CoA beads were incubated in the absence or presence of either 30 or 300 μM ATP, and the amounts of bound (CoA-bound) and released (flow-through) NME1 were visualized by immunoblotting (top). Bottom panels show the same blots probed with an anti-phosphohistidine antibody.

NME1 recognizes the 3′ phosphate group that distinguishes CoA from ADP with high specificity via five direct and two water-mediated H bonds (Fig. 3, A and C). Although the nucleotide moiety of a CoA molecule lacking this 3′ phosphate, dephospho-CoA, has the same structure as ADP, dephospho-CoA is predicted to bind NME1 poorly because its pantoyl moiety would sterically hinder the adjacent β phosphate from becoming buried in the active site, thereby prohibiting the canonical ADP binding mode. NME1 incubated with dephospho-CoA yielded a native MS spectrum indistinguishable from that of the unliganded protein, confirming that NME1 does not bind dephospho-CoA (Fig. 4A). Thus, the 3′ phosphate of CoA is a critical feature required for NME1 recognition.

Since NME1 recognizes ADP as well as the ADP-containing end of CoA, it might conceivably bind structurally related metabolites such as nicotinamide adenine dinucleotide (NAD), flavin adenine dinucleotide (FAD), and ADP-ribose. To verify this, we bound NME1 to CoA beads and tested metabolites for their ability to elute the protein. As expected, NME1 was efficiently released from the beads by ATP and CoA but not by dephospho-CoA (Fig. 4B). NAD, FAD, and ADP-ribose were equally ineffective at eluting NME1, revealing that NME1 does not promiscuously bind ADP-containing metabolites (Fig. 4B). Like dephospho-CoA, the lack of a 3′ phosphate group prevents these metabolites from binding like CoA, while their ADP-linked moieties sterically hinder them from burying their β phosphate in the active site like ADP. These data suggest that CoA/acyl-CoA recognition by NME1 may be modulated by cellular ADP and ATP levels but not by other common ADP-containing metabolites.

### CoA binding and histidine phosphorylation are mutually inhibitory

Although CoA binds to the NDP/NTP binding site of NME1, previous studies have reached disparate conclusions regarding the effect of CoA on the NDPK activity of NME1/2, with one study reporting competitive inhibition by CoA in a coupled enzymatic assay (*14*) and another reporting no substantial inhibition by CoA or short-chain acyl-CoA in a chromatography-based assay (*15*). Moreover, it is unknown whether the phosphoenzyme intermediate that mediates NDPK activity is able to bind CoA. Another metabolite, 3′-phosphoadenosine 5′-phosphosulfate, which chemically resembles the CoA nucleotide and adopts a similar ligand binding mode (fig. S9A), was reported to inhibit *Dictyostelium discoideum* NDPK with threefold higher binding affinity than ADP (*25*). Furthermore, structurally aligning CoA-bound NME1 with an NDPK phosphoenzyme intermediate (*30*) closely juxtaposes the CoA 3′ and histidine phosphate groups, which would yield a strong steric and electrostatic repulsion (fig. S9B). These observations predict that CoA binding should be mutually exclusive with NDPK activity and with phosphoenzyme formation.

To verify this hypothesis, we incubated NME1 with immobilized CoA in the absence or presence of ATP and detected the total and phosphorylated protein in bound and unbound fractions by immunoblotting. Whereas in the absence of ATP, most NME1 remained in the bound fraction, the addition of ATP caused the release of phosphorylated NME1 from the CoA beads, confirming that CoA binds poorly to the phosphoenzyme intermediate (Fig. 4C). For further proof, we used native MS to analyze NME1 hexamers following incubation with either CoA, ATP, or both ligands. Incubation with CoA resulted in a predominance of NME1 hexamers bound to three or four CoA ligands, consistent with a ~60% binding site occupancy (Fig. 4A, bottom right, and fig. S10A). Coincubation with both CoA and ATP resulted in a leftward shift of peak intensities, revealing a prevalence of NME1 hexamers bound to only one or two CoA ligands and a ~20% binding site occupancy (Fig. 4A, top right and fig. S10B), confirming that ATP inhibits CoA binding. Incubation with ATP did not yield any detectable nucleotide-bound hexamers, but inspection of individual peaks revealed closely spaced sub-peaks corresponding to phosphorylated hexamers (Fig. 4A, bottom inset), analogous to the closely spaced bands seen on a native gel (Fig. 2B). Most hexamers carried one to three phosphate groups, consistent with the monophosphorylation of 32% of NME1 monomers (Fig. 4A, middle right, and fig. S10C). In contrast, following coincubation with both ATP and CoA, the phosphorylation level of the unbound NME1 hexamer was only 16%, and this level progressively decreased to 10% for hexamers bound to either one, two, or three CoA ligands, directly demonstrating that CoA inhibits histidine phosphorylation (Fig. 4A, top right, and fig. S10B). Together, these data provide compelling evidence that the NDPK and CoA-binding functionalities of NME1 are mutually inhibitory.

### NME1/2 repress lipogenesis in the liver

**Fig. 5.**
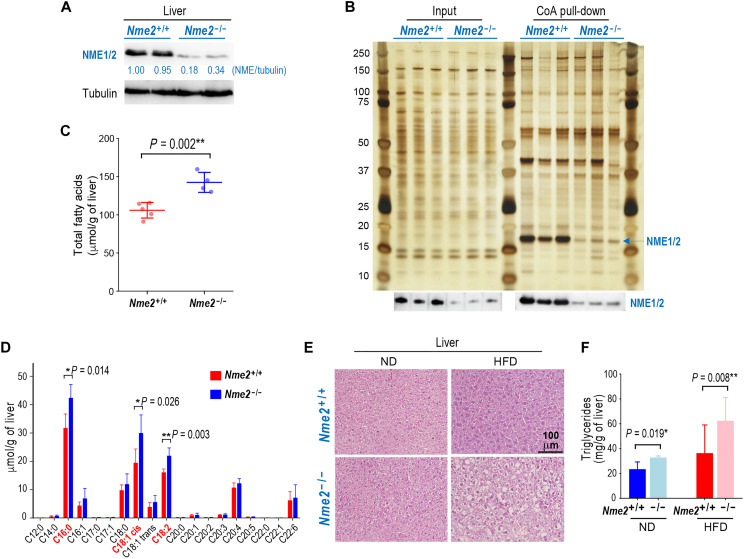
NME1/2 repress lipogenesis in the liver. (**A**) Total amounts of NME1 and NME2 were visualized in the liver of *Nme2^+/+^* and *Nme2*^−/−^ mice. Values represent the NME1/2 signal intensity relative to tubulin as measured by gel densitometry. (**B**) Liver extracts from three different *Nme2^+/+^* and *Nme2*^−/−^ mice were used in a CoA-bead pull-down experiment as described in Fig. 1B. Input (0.5%) and CoA-bound materials were visualized on a silver-stained gel, and a fraction was used to show the presence of NME1/2 in the corresponding samples by immunoblotting (bottom). (**C**) Values corresponding to the total amount of fatty acids measured in liver extracts from five different *Nme2^+/+^* and four *Nme2*^−/−^ mice are shown. Error bars represent SD. (**D**) The amounts of different fatty acid species (indicated as C*x*:*y*, where *x* and *y* are the number of carbon atoms and double bonds, respectively) in liver extracts from five different *Nme2^+/+^* and four *Nme2*^−/−^ mice are shown. Error bars represent SD. Fatty acid species showing the highest increase in *Nme2* ko liver are highlighted in red. (**E**) *Nme2^+/+^* and *Nme2*^−/−^ mice were subject to normal diet (ND; four *Nme2^+/+^* and five *Nme2*^−/−^ mice) or high-fat diet (HFD; six *Nme2^+/+^* and seven *Nme2*^−/−^ mice). Representative images of hematoxylin and eosin–stained paraffin-embedded liver sections from *Nme2^+/+^* and *Nme2*^−/−^ male mice after 6 weeks of ND or HFD are shown as indicated. Scale bar, 100 μm. (**F**) Triglyceride concentrations were measured in liver extracts from 4 *Nme2^+/+^* and 4 *Nme2*^−/−^ mice in ND and from 10 *Nme2*^+/+^ and 12 *Nme2*^−/−^ after 6 weeks of HFD. Error bars represent SD. *P* values are indicated. In all panels, statistical significance is indicated by symbols * and ** for *P* values <0.05 and < 0.01, respectively (Student’s *t* test).

We next turned to a mouse ko model to explore the potential importance of NME1/2 for CoA/acyl-CoA–utilizing pathways. Since an *Nme1/2* double ko is embryonic lethal, we decided to work on *Nme2*^−/−^ (ko) mice, which present only a mild phenotype (*12*, *31*). As expected from its high sequence similarity, NME2 exhibits an ATP-sensitive CoA binding activity comparable to that of NME1 (fig. S11A). An initial screen of various tissues revealed that the total level of NME1/2 is dramatically reduced in the liver of *Nme2*^−/−^ mice compared to WT (*Nme2*^+/+^) mice (Fig. 5A). Accordingly, analysis of transcriptomic data (presented below) from *Nme2*^+/+^ and ^−/−^ mouse liver showed that, as expected, *Nme2* mRNA was detected at a background level in *Nme2*^−/−^ mouse liver. In the absence of NME2, *Nme1* expression was also significantly down-regulated (fig. S11B). A CoA pull-down approach confirmed that NME1/2 were highly abundant CoA-binding factors in WT mouse liver extracts and were drastically reduced in those from *Nme2*^−/−^ mice (Fig. 5B), identifying the liver as an expedient model system for subsequent experiments.

Since the liver is one of the most active sites for DNL, which depends on a continuous supply of AcCoA, we focused on the role of NME2 in this process. MS-based lipidomic analysis of liver extracts from *Nme2*^+/+^ and ^−/−^ mice revealed that the disruption of *Nme2* resulted in a significant increase in the total fatty acid content (Fig. 5C). A detailed analysis of hepatocyte fatty acids showed significantly increased levels of C16:0, C18:1*cis*, and C18:2 fatty acid species in *Nme2*^−/−^ mice (Fig. 5D). These findings demonstrate that NME2 negatively regulates the accumulation of cellular fatty acids.

### NME2 regulates a liver cell response to an HFD challenge

Since an HFD is known to suppress DNL (*32*–*35*), and we found NME2 to counteract fatty acid accumulation, we surmised a role for NME1/2 in the HFD-dependent repression of DNL. To test this idea, we fed *Nme2*^+/+^ and *Nme2^−/−^* mice with an HFD or a normal diet (ND) for 6 weeks. Unlike WT mice, the *Nme2*^−/−^ mice exhibited clear signs of liver steatosis (Fig. 5E) and increased liver triglyceride levels (Fig. 5F) under HFD. These results suggest that, under an HFD challenge, *Nme2*^−/−^ liver cells fail to suppress the expression of genes promoting liver lipogenesis.

**Fig. 6.**
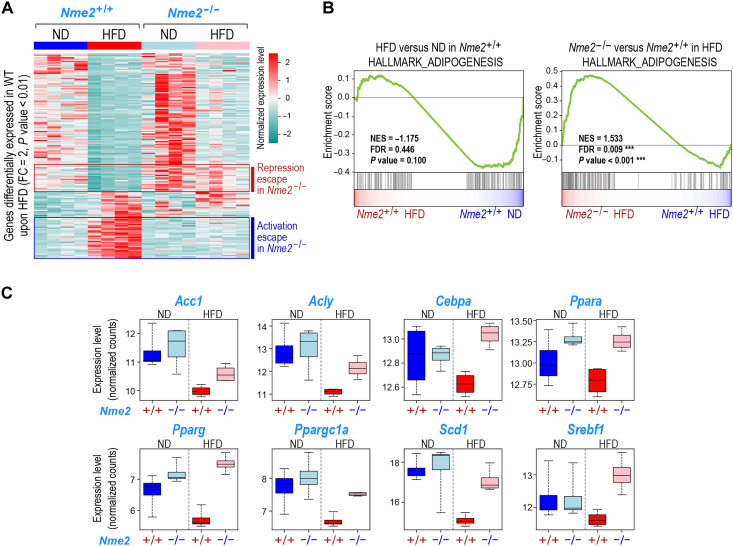
NME2 regulates the liver cell response to an HFD challenge. (**A**) RNAs extracted from liver of four mice of each indicated genotype kept under ND or fed for 6 weeks with an HFD were subjected to RNA-seq. The expression of genes differentially expressed between the liver of HFD and ND fed *Nme2*^+/+^ mice (with a fold change absolute value >2 and a *P* value <0.01) is shown as a heatmap in *Nme2*^+/+^ and *Nme2^−/−^* mice for both conditions as indicated. Hierarchical clustering enables the identification of genes that escape HFD-dependent repression or activation in *Nme2^−/−^* mice (indicated on the right side of the heatmap by red and blue bars, respectively). (**B**) GSEA plots showing a down-regulation of the gene set corresponding to genes involved in adipogenesis in the hepatocytes of *Nme2*^+/+^ mice liver treated with HFD for 6 weeks compared with ND (reference, left). The same gene set is significantly enriched in hepatocytes of *Nme2^−/−^* mice compared to *Nme2^+/+^* mice (reference) under HFD (right), showing that the HFD-induced repression of this gene set is abolished in *Nme2^−/−^* hepatocytes. (**C**) Box plots representing the distribution of the expression levels of the indicated individual genes in liver from *Nme2*^+/+^or *Nme2^−/−^* mice under ND or HFD are shown. The values correspond to the normalized RNA-seq read counts.

To verify this hypothesis, we performed a transcriptomic analysis of liver cells from *Nme2*^+/+^ and *Nme2^−/−^* mice fed normally (ND) or undergoing an HFD challenge. In *Nme2*^+/+^ mice, the HFD challenge induced changes in the transcriptional activity of hepatocytes characterized by the marked repression and activation of 107 and 52 genes, respectively (absolute fold change ≥ 2; *P* value ≤ 0.01) (Fig. 6A, *Nme2*^+/+^, ND versus HFD). *Nme2*^−/−^ mice exhibited a notably different pattern, revealing that the HFD-dependent gene activation was abolished for the majority (~70%) of genes in the latter group, while ~20% of genes escaped the HFD-dependent transcriptional repression (Fig. 6A, *Nme2*^−/−^, ND versus HFD). A gene set enrichment analysis (GSEA) revealed that HFD repressed a considerable number of genes involved in adipogenesis in the liver of *Nme2*^+/+^ mice (Fig. 6B, left), as previously reported (*35*). This HFD-dependent repression only occurred in the presence of NME2 (Fig. 6B, right). More specifically, in WT hepatocytes from mice under HFD, the expression of known master transcription activators of genes involved in lipid metabolism, such as peroxisome proliferator–activated receptor α (*PPAR*α), *PPAR*γ, *PPARγC1*α (*PGC1*α), and the lipogenic gene couple *SREBF1/CEBP*α, was repressed, whereas in the absence of NME2, these genes either failed to be repressed or were even up-regulated despite the HFD challenge (Fig. 6C). The continuous expression of these master transcription factors explains the inability of *Nme2*^−/−^ hepatocytes to repress lipogenic genes in response to an HFD challenge.

The GSEA also revealed specific genes that were up-regulated in response to an HFD challenge in an NME2-dependent manner. These genes are mostly involved in the interleukin-6 (IL-6)/Janus kinase (Jak)/signal transducer and activator of transcription 3 (Stat3), interferon α and γ, and tumor necrosis factor–α (TNFα) pathways (fig. S12A), and include a number that could be directly regulated by a gain in H3K9ac at their +1 and +2 nucleosomes (see the next section and table S4). All these gene expression pathways have been previously shown to play a critical protective role during HFD-induced liver injury and liver regeneration (*36*). Together, the above transcriptomic analyses suggest that NME2 could be a general regulator of a protective transcriptional response of liver cells to an HFD challenge that acts by suppressing DNL and activating a protective cytokine response.

To exclude a role for AMPK (known to phosphorylate and inactivate ACC1) in the regulation of lipogenesis by NME1/2, we used immunoblotting to monitor the overall expression and phosphorylation of ACC1 and AMPK proteins (fig. S13A). As expected, the variations of ACC1 mirrored that of its encoding mRNA under the different conditions tested (compare fig. S13A and Fig. 6C, ACC1). The level of inactivating ACC1 phosphorylation by AMPK remained unchanged between *Nme2*^−/−^ and *Nme2*^+/+^ livers, consistent with unchanged levels of AMPK activation (P-AMPK/AMPK ratio, fig. S13A) and of cellular ATP, ADP, and AMP under the different tested conditions (fig. S13B). Thus, AMPK signaling is not involved in ACC1 regulation or in the increased DNL under these conditions. Overall, these data demonstrate that NME2 directly controls the liver cell response to HFD.

### NME2 modulates H3K9ac distribution at gene TSSs

We next investigated whether the transcriptional regulation observed in liver under HFD involved an NME2-dependent control of histone acetylation. We focused on H3K9ac as a histone mark known to be associated with active gene transcription and responsive to a metabolic change (*37*, *38*). As shown in fig. S12B, an HFD challenge or *Nme2*^−/−^ did not change the global level of H3K9ac in hepatocytes, which was comparable across all tested conditions. Measurement of the cellular CoA and AcCoA concentrations showed that, although the HFD challenge increased the amount of CoA in both WT and ko cells, it did not significantly alter the amount of AcCoA in WT cells (fig. S12C). In contrast, in the absence of NME2, an HFD challenge led to a significant (1.65-fold relative to ND) increase in AcCoA (fig. S12C), which could contribute to the observed increase in fatty acid synthesis (Fig. 5, C and D).

**Fig. 7.**
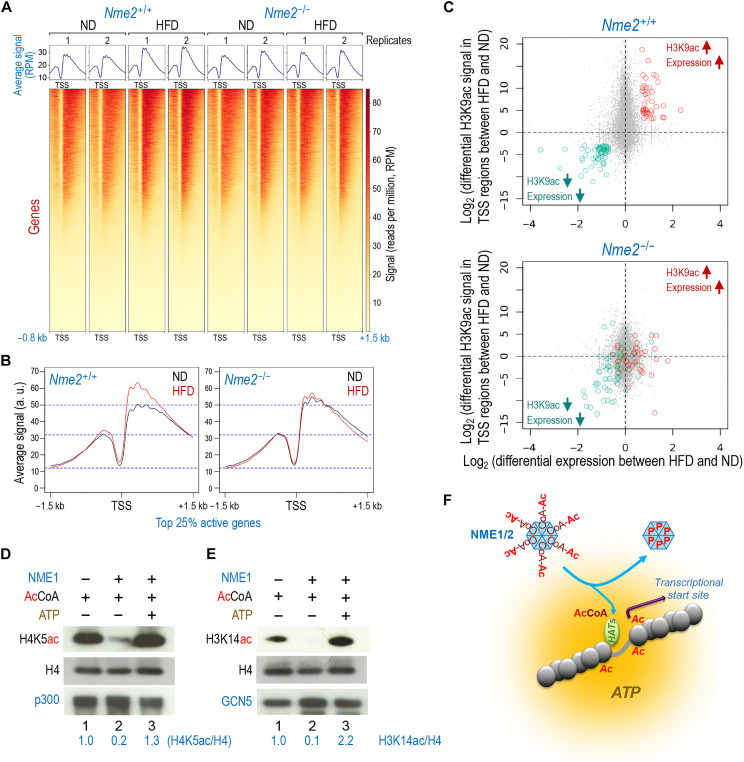
NME2 modulates H3K9ac distribution at gene TSSs. (**A**) Purified mouse liver nuclei (*N =* 2 per condition) were MNase-digested. Mono-nucleosomes were immunoprecipitated using an anti-H3K9ac antibody. Normalized read counts at the corresponding gene TSS were visualized. Normalization assumed similar total H3K9ac levels in all samples, as confirmed in fig. S12B. TSS regions are ranked by mean signal value across all conditions, from highest to lowest. (**B**) The mean values of two independent H3K9ac ChIP read counts from HFD (red) and ND (black) in *Nme2^+/+^* and *Nme2^−/−^* mice were plotted over ±1.5 kb centered on TSS regions corresponding to the 25% top most highly expressed genes (according to the transcriptomic data of *Nme2^+/+^* mouse liver samples under ND, *n* = 3500 TSS). (**C**) The difference of the mean H3K9ac ChIP-seq values between HFD and ND for each TSS-0.8 + 1.5 kb region was plotted against the differential expression values (log_2_ of the fold change) of the corresponding genes between the same conditions. Considering the *Nme2^+/+^* liver samples (top), two groups of TSS/genes were selected, either presenting both an enhanced expression and an increased TSS H3K9ac level between HFD and ND (red dots) or showing a reduced expression and a decreased H3K9ac level between HFD and ND (green dots). The same genes are visualized on the plot corresponding to the *Nme2^−/−^* liver in the bottom (i.e., genes symbolized by red or green dots are the same in both panels). (**D**) Purified mononucleosomes and Flag-p300 were incubated with equimolar AcCoA and purified NME1 (49 μM) as indicated and histone acetylation (H4K5ac) was detected by immunoblotting. (**E**) The same experiment as in (D), but p300 was replaced by Flag-GCN5, and H3K14ac was immunodetected. (**F**) Scheme showing the proposed role for NME1/2 in releasing AcCoA in the active chromatin region harboring an ATP-rich environment in the vicinity of HATs and subsequent targeted histone acetylation.

Using a chromatin immunoprecipitation sequencing (ChIP-seq) approach, we then investigated whether NME2 was involved in controlling the distribution of H3K9ac around gene transcriptional start sites (TSSs), which would explain the transcriptional reprograming of HFD-dependent genes. Figure 7 (A and B) shows that, in *Nme2*^+/+^ liver cells, the HFD challenge led to an increase of H3K9ac at gene TSSs, which was especially pronounced at active genes. The enhanced acetylation occurred on nucleosomes (primarily at positions +1, +2, and +3) downstream of the TSS of active genes but not on upstream nucleosomes (Fig. 7B, left). This redistribution of H3K9ac did not occur in *Nme2*^−/−^ liver cells (Fig. 7B, right).

Enhanced H3K9ac downstream of TSSs is known to promote RNA polymerase II pause release (*39*) and thereby to enhance transcription. To correlate the redistribution of H3K9ac at TSS-associated nucleosomes with transcriptional activity (*40*), we highlighted genes whose acetylation and transcription levels either both increased or both decreased under HFD compared to ND in the liver of *Nme2*^+/+^ mice (Fig. 7C, top, red and green dots, respectively). Thus, the redistribution of H3K9ac on the TSS regions of these genes is associated with the transcriptional response to HFD in *Nme2*^+/+^ hepatocytes. In *Nme2*^−/−^ hepatocytes, the HFD-dependent H3K9ac redistribution and transcriptional response to HFD was lost for many of these genes, including nearly all those that normally showed a combined increase in expression and TSS-associated H3K9ac (Fig. 7C, bottom, red dots). NME2 seems required for the combined decrease in expression and TSS-associated H3K9ac for only a subset of genes under HFD (Fig. 7C, bottom, green dots). These include the critical regulator of DNL, *Acc1*, suggesting that the NME2-dependent reprogramming of H3K9 acetylation could mediate *Acc1* repression in response to the HFD challenge (Fig. 6C).

### HAT activity in vitro is inhibited by NME1 and rescued by ATP

The above findings point to NME1/2 as critical mediators of an increase in H3K9 acetylation at nucleosomes localized downstream of a defined number of genes’ TSSs and hence of the enhancement of their transcriptional activity (Fig. 7, A to C). Because enzymatic activity depends on substrate availability and since NME1/2 bind the histone acetyltransferase (HAT) substrate AcCoA competitively with ATP (Fig. 4C; fig. S10, A and B; and fig. S11A), we wondered whether NME1/2 might directly modulate HAT activity in an ATP-dependent manner. To this end, we incubated mononucleosomes with AcCoA and with the purified HAT p300 or GCN5 (Fig. 7, D and E, lanes 1 to 3), followed by immunoblotting to visualize histone lysines typically acetylated by these HATs (H4K5 and H3K14; Fig. 7, D and E, lane 1). The addition of equimolar purified NME1 strongly inhibited histone acetylation by both HATs (Fig. 7, D and E, lane 2), while the simultaneous addition of equimolar ATP completely restored acetylation, presumably by antagonizing AcCoA sequestration by NME1 (Fig. 7, D and E, lane 3). These results point to NME1/2 as AcCoA carrier molecules that are able to release their AcCoA load in response to ATP. We speculate that, after the HFD-induced shutdown of DNL and a decrease in AcCoA usage in the cytosol, a fraction of AcCoA-loaded NME1/2 could become exposed to a high level of locally produced ATP around transcriptionally active chromatin (*41*, *42*), where ATP fuels various chromatin remodelers, and releases their AcCoA in a targeted manner, thereby accounting for the observed increase in H3K9ac mediated by NME1/2 specifically in active chromatin regions (Fig. 7F).

## DISCUSSION

In this study, we showed that NME1 binds AcCoA and CoA with similar affinity via a binding mode distinct from that of canonical ADP and ATP substrates and critically dependent on the CoA 3′ phosphate group. This plasticity of ligand recognition is primarily achieved through a versatile array of structural waters and the lack of base-constraining H-bonding groups in the nucleotide-binding site. These structural features make NME1/2 uniquely adapted to recognize ATP and CoA through the same binding site and thereby monitor the cellular balance between these key metabolites. This observation, together with the prevalence of NME1/2 over other cellular CoA-binding factors in our CoA pull-downs, led us to consider the role played by NME1/2 in lipogenesis, a pathway controlled by the cellular energy status and a major consumer of AcCoA, the most abundant cellular CoA derivative. The absence of a compensatory NME1/2 accumulation in the liver of *Nme2*^−/−^ mice identified this organ, a major site of lipogenesis, as a good model for exploring the role of NME1/2 in fatty acid synthesis and accumulation.

A lipidomic analysis of the liver from *Nme2*^+/+^ and *Nme2^−/−^* mice revealed a repressive role for NME1/2 in lipogenesis. We observed that NME2 depletion abolishes the well-documented ability of hepatocytes to repress DNL under HFD. Exploring the underlying mechanism revealed that, whereas an HFD challenge normally represses the expression of major lipogenic genes, as previously reported (*35*), in *Nme2*^−/−^ hepatocytes under the same HFD conditions, these genes remain active. More specifically, we observed that, while HFD ordinarily represses the expression of master transcription factors involved in lipid metabolism such as PPARα, PPARγ, PPARγC1α (PGC1α), and SREBF1/CEBPα, in the absence of NME2, the expression of these factors persists. In agreement with the role of these factors as transcription drivers, we observed that a series of other genes involved in adipogenesis also escape repression under HFD in *Nme2*^−/−^ liver cells. HFD also induces the expression of multiple genes known to play a protective role during liver regeneration, including those involved in the IL6/Jak/Stat3, interferon α and γ, and TNFα pathways (*36*). These transcriptomic analyses therefore suggest that NME1/2 could be general regulators of the liver response to stressful assaults.

Our findings raise two interesting questions: How do NME1/2 repress DNL in response to HFD, and how does DNL escape repression in the absence of NME1/2? Regarding the former, the genome-wide mapping of H3K9ac in *Nme2*^+/+^ and *Nme2^−/−^* liver cells from mice under ND or HFD challenge revealed the occurrence of an HFD-induced increase of H3K9ac at active gene TSSs. This specific H3K9ac accumulation was completely abolished in the absence of NME2. On the basis of the known competition between DNL and histone acetylation (*43*–*45*), a possible explanation could be that the NME1/2-dependent down-regulation of DNL under HFD makes the pool of NME1/2-bound AcCoA more available for histone acetylation. However, our structural and biochemical studies indicate that AcCoA binding by NME1/2 restricts AcCoA usage by AcCoA-consuming enzymes, including HATs. Our data also show that ATP could release AcCoA from NME1/2, suggesting that an NME1/2-mediated increase in histone acetylation needs an ATP-rich environment. ATP can be produced in cell nuclei by NUDIX5 using ADP-ribose to generate ATP, a process critical for hormone-dependent chromatin remodeling and subsequent transcriptional activation (*41*, *42*). We could reconstitute this process in vitro by showing that ATP can release AcCoA from NME1 and provide it to HATs for histone acetylation. On the basis of these data, we propose that the HFD-induced shutdown of DNL makes a fraction of AcCoA-loaded NME1/2 available for histone acetylation and that the ATP-rich environment of active chromatin regions makes this AcCoA specifically available to HATs usually present at transcriptionally active chromatin.

Regarding the mechanism underlying the lack of DNL repression in *Nme2* ko liver, the master lipogenic transcription factor SREBP1 seems likely to play a critical role. Whereas an HFD challenge led to a down-regulation of SREBP1 expression in WT liver cells, in *Nme2* ko liver cells, SREBP1 expression escaped HFD-induced down-regulation and even exhibited an enhanced expression. Moreover, SREBP1 acetylation at K289 and K309 could further activate this transcription factor (*46*). Mechanistically, we speculate that, in the absence of NME2, the increased availability of cytoplasmic AcCoA and an increased acetylation of the SREBP1 cytoplasmic precursor could lead to hyperactivity of this transcription factor after its cleavage and import into the nucleus, where it could bind its own promoter and activate its own expression (*47*) as well as that of all downstream lipogenic genes. In the absence of NME1/2 and the inability of HFD to repress DNL, AcCoA could continue to fuel DNL and to be used for nonprogrammed acetylation of cytoplasmic proteins, including that of SREBP1.

More generally, our findings suggest that the ability of NME1/2 to bind CoA/AcCoA could play a major regulatory role in different critical cellular processes dependent on AcCoA usage. For instance, the ability of NME1/2 to sequester AcCoA in an ATP-dependent manner might provide an additional level of control of lipogenesis that could potentially synergize with the well-known AMPK-dependent energy sensing mechanism. High cellular ATP concentrations would not only prevent the inactivating phosphorylation of ACC1 by AMPK but, by inhibiting sequestration by NME1/2, would also make more AcCoA available to ACC1, leading to enhanced DNL and a consequent increase of ACC1 accumulation (*48*–*50*). We note that NME1/2 are sufficiently abundant to have an appreciable buffering effect on the free CoA/AcCoA levels in the cytosol. Our measurements indicate that the combined concentration of CoA and AcCoA in mouse liver cells remains below 200 μM, consistent with previous estimates in rat liver cells (90 to 100 μM) (*51*, *52*) and mammalian cells in general (20 to 140 μM) (*53*). However, most of these molecules (90 to 95%) will be in the mitochondria (*52*, *54*), and an additional fraction will be bound by various carriers and enzymes, and so the available cytosolic concentration is likely to be in the 1 to 20 μM range. NME proteins are among the 100 most abundant cellular proteins from *Escherichia coli* (*55*) to human (*56*). Although the NME1/2 concentration in mouse liver cells is unknown, a study of mouse fibroblasts reported an NME1 copy number of >21 million molecules per cell (*57*). Assuming a cell volume of 2000 μm^3^ (*58*), this implies an NME1 concentration of ~18 μM, closely matching the *K*_d_ value we measured for CoA binding. The fact that both values are commensurate with the cytosolic CoA/AcCoA concentration means that a considerable fraction (up to 40 to 50%) of these molecules are potentially bound by NME1/2, in line with the observed prevalence of NME1/2 over other cellular CoA/AcCoA-binding factors in our CoA pull-downs.

In conclusion, the work reported here highlights the function of NME1/2 as a major regulator of AcCoA usage in hepatocytes and responsible for a liver protective gene expression program. In agreement with this finding, a previous study investigating genetic factors controlling liver injury susceptibility identified NME1/2 as important determinants in protecting the liver against an injury-inducing treatment (*59*). Therefore, NME1/2 may be a general regulator of a protective liver response, as well as a general regulator of AcCoA usage in different tissues, in various pathophysiological contexts.

## MATERIALS AND METHODS

### *E. coli* culture

*E. coli* BL21-Gold(D3) (Agilent no. 230132) bacteria were transformed by pETM-11-Nme1/T94D-mutant, and pGEX-4T-1-Nme1/T94D-mutant and clones were selected on LB agar plates containing kanamycin and ampicillin, respectively, and cultured at 37°C. One Shot TOP10 Chemically Competent *E. coli* (Invitrogen no. C404010) were cultured at 37°C to amplify the plasmids pETM-11-Nme1/T94D-mutant and pGEX-4T-1-Nme1/T94D-mutant.

### Animals

All mouse experiment protocols were approved by the official ethics committee of the University Grenoble Alpes (ComEth, C2EA-12), and all the investigators directly involved in the care and breeding of the mice have an official animal-handling authorization obtained after 2 weeks of intensive training and a final formal evaluation. All mice were in a light-regulated colony room (12 hours light/12 hours dark), with food and water available ad libitum. Heterozygotes *Nme2* KO male and female mice [C57BL/6N-Nme2tm1.1(KOMP)Vlcg/MbpMmucd] were obtained from MMRRC (Mutant Mouse Resource & Research Centers supported by the National Institutes of Health, stock no.: 048819-UCD). Mice were bred in an animal core facility [Grenoble High Technology Animal Facility (PHTA), University Grenoble Alpes]. Both male and female mice of 2 to 5 months of age were used for all studies.

### Lysate preparation for CoA pull-down

#### Total testis

For each CoA pull-down experiment, two adult male mice were euthanized and the testes were collected. After removing the albuginea, the seminiferous tubules were directly immersed in 1 ml of lysis buffer [20% glycerol, 3 mM MgCl_2_, 50 mM Hepes, 250/500 mM KCl, 0.1% NP40, 1 mM dithiothreitol (DTT), and 1 mini-tablet of EDTA-free protease inhibitor (Roche, no. 04693159001)], homogenized for up to 1 min with homogenizer (Heidolph RGL 55/1), and incubated for 30 min on ice. The sample was then centrifugated at 394*g* for 10 min at 4°C, and the supernatant extract was used immediately for the purification of the CoA-bound proteins by CoA pull-down. The 500 mM KCl lysate was diluted to 250 mM with buffer before proceeding.

#### Spermatogenic cells

The enrichment (or fractionation) of spermatogenic cells at different stages of their maturation from the testes of six mice was performed as described in detail in (*60*, *61*). Briefly, the seminiferous tubules were incubated with collagenase/phosphate-buffered saline (PBS) (1 mg/ml) for 15 min at 35°C, dissociated by pipetting for 10 min, and filtered through a 100-μm filter. Total germ cells were pelleted, suspended in 0.5% bovine serum albumin (BSA)–Dulbecco’s modified Eagle’s medium/F12 (Gibco, no. 31331028) and sedimented through 2 to 4% BSA gradient for 70 min at 4°C. The fractions were then collected and examined under a phase-contrast microscope. Fractions of total germ cells (spermatogenic cells before sedimentation) or respectively enriched in pachytene spermatocytes, in round spermatids, and in elongating and condensing spermatids were pooled for the preparation of cell lysates. In each CoA pull-down experiment, the cell pellets were incubated in 125 μl of lysis buffer (20% glycerol, 3 mM MgCl_2_, 50 mM Hepes, 500 mM KCl, 0.1% NP40, 1 mM DTT, and mini-tablet of EDTA-free protease inhibitor) and treated as previously described for total testis lysate.

#### Liver

Fifty milligrams of liver from *Nme2* WT or *Nme2* KO mice were incubated in 500 μl of 250 mM lysis buffer and treated as previously described for total testis lysate.

### Purification of CoA-bound proteins

CoA-agarose (C7013-500MG, Sigma-Aldrich) beads were suspended in 3.5 ml of 10 mM sodium acetate (pH 6.0) and stored at −20°C. For each experiment, 50 μl of the CoA-agarose mixture was washed three times in lysis buffer (250 mM KCl) and mixed with 1 ml of each of the lysates (respectively from testis, spermatogenic cells, or liver) and incubated with rotation overnight at 4°C. The CoA-agarose beads were then washed three times in the lysis buffer, followed by a final wash with PBS. Half of the beads were then incubated for 1 hour at 4°C in 50 μl of 5 mM free CoA (C4282, Sigma-Aldrich) (dissolved in H_2_O), and the other half was incubated in 50 μl of H_2_O (Control). Eluted proteins were collected following a centrifugation step (1650*g*, 2 min, 4°C). Ten microliters of the eluted proteins was separated by migration on a NuPAGE 4 to 12% bis-tris protein gel (Invitrogen) and silver-stained. Another 10 μl was analyzed by Western blotting (WB). The rest of the eluted proteins were analyzed by MS.

### Gene cloning

The full-length codon sequence of mouse *Nme1* and *Nme2*, was cloned in pGEX-4T-1 (GST tag) and pETM-11 (His tag) vectors for protein production in BL21 *E. coli*. The T94D mutation was introduced in the plasmids by polymerase chain reaction (PCR) using the QuickChange Multi-Site Directed Mutagenesis kit (no. 200514, Agilent Technologies). The sequence of the primer used to introduce the mutation T94D in the *Nme1* sequence of plasmids is reported in key resources table. Every plasmid was sequence-verified.

### Recombinant mouse GST-NME1 purification

*E. coli* BL21-Gold(D3) (230132, Agilent) were transformed with 50 ng of either GST-Nme1 or GST-NME1-T94D-mutant vector (pGEX-4T-1) and cultured from a single colony. Once 600 ml of culture reached an optical density at 600 nm (OD_600nm_) of 0.8, protein expression was induced by adding 0.2 mM isopropyl-β-d-thiogalactopyranoside (IPTG), followed by incubation with shaking overnight at 18°C. The cells were pelleted at 9000*g* at 4°C for 30 min, resuspended in 10 ml of 50 mM tris-HCl (pH 7.4), 500 mM NaCl, and 0.3% Triton X-100 plus protease inhibitors, and disrupted by sonication for 2 min using cycles of alternating on/off pulses (5-s On/5-s Off) at 4°C. Then, the lysate was clarified by centrifugation at 48,000*g* 4°C for 20 min and incubated for 1 hour at room temperature (RT) with 2 ml of pre-washed Glutathione Sepharose (Cytiva, catalog no. 17-5132-01). After three washes with a buffer containing 50 mM tris-HCl (pH 7.4), 500 mM NaCl, and protease inhibitors, GST-NME1 was eluted three times for 30 min each in 1 ml of a buffer containing 50 mM tris-HCl (pH 7.4), 500 mM NaCl, and 25 mM glutatione. The GST-NME1 proteins (WT and T94D-mutant) were collected and adjusted to 1 mg/ml in the same buffer and subsequently flash-cooled in liquid nitrogen for storage at −80°C.

### Recombinant mouse NME1 purification for biochemical and structural analyses

*E. coli* BL21-Gold(D3) (230132, Agilent) were transformed with 50 ng of the His-Nme1/T94D-mutant vector (pETM-11) and cultured from a single colony. Once 1 liter of culture reached an OD_600nm_ of 0.8, protein expression was induced with 0.2 mM IPTG, and the culture was incubated with shaking overnight at 18°C. The cells were pelleted by centrifugation at 9000*g* at 4°C for 30 min and stored at −80°C until further use. The pellet was subsequently thawed and resuspended in 12.5 ml of phosphate buffer 0.1 M (pH 7.4), containing 150 mM NaCl, 25 mM imidazole, and protease inhibitors (buffer A), and the cells were disrupted by sonication using 60 cycles of alternating on/off pulses (2-s on/10-s off) at 4°C. Then, the lysate was clarified by centrifugation at 48,000*g* at 4°C for 1 hour and applied to a 5-ml HisTrap HP prepacked column (Cytiva no. 175248). The column was then washed with buffer A (15 to 20 column volumes), and NME1 was eluted in a 100-ml linear gradient ranging from 0 to 500 mM imidazole using 0.1 M phosphate buffer (pH 7.4), 150 mM NaCl, and 500 mM imidazole (buffer B) and collected. The N-terminal 6-His tag was cleaved by Tobacco Etch Virus (TEV) protease (1 mg protease per 15 mg of His-NME1) during dialysis against phosphate buffer with 150 mM NaCl overnight at 4°C. After removal of the cleaved His-tag by a 5-ml HisTrap HP column, NME1 was collected from the flow-through and subjected to size exclusion chromatography using a Superdex 200 16/60 column (Cytiva), which was previously equilibrated with phosphate buffer containing 150 mM NaCl. NME1 appeared at an elution volume of approximately 170 ml. The protein was collected, pooled, concentrated to 11 mg/ml using a 10-kDa cutoff centrifugal filter (Amicon Ultra-15 10K, Millipore no. UFC901096), flash-cooled in liquid nitrogen, and stored at −80°C.

### In vitro histidine phosphorylation assay

In vitro autophosphorylation of the WT or T94D mutant forms of untagged or GST-tagged NME1 was performed in TMD buffer [20 mM tris-HCl (pH 8.8), 5 mM MgCl_2_, and 1 mM DTT] with ATP at RT in a 20-μl reaction volume. In a reaction reported in fig. S2B, 0.23 mM NME1 was incubated with 0.5 mM ATP for 10 min, and subsequently, 0.028 mM phosphorylated NME1 was incubated with GDP (0.1, 0.2, and 0.4 mM) for another 10 min. In fig. S2A, 5.7 mM NME1 was incubated with 100 μM ATP for 10 min. In fig. S8C, GST-WT and T94D mutant (1 μg) were incubated with 100 μM ATP for 10 min and subsequently with GDP (200 μM) for another 10 min. In fig. S8D, WT and T94D mutant (1 μg) were incubated with ATP (0 to 100 μM) for 1 min. Each reaction was arrested by adding 5 μl of 5× gel loading buffer [10% SDS, 250 mM tris-HCl (pH 8.8), 0.02% bromophenol blue, 50% glycerol, 50 mM EDTA, and 500 mM DTT], and 10 μl of the solution was analyzed immediately by WB to detect 1-*P*-histidine phosphorylation. WB was performed as reported in detail in (*62*) with some modifications. Briefly, the stacking gel buffer was adjusted to pH 8 to 9, and the samples were not heated. All electrophoresis steps were performed at 4°C, and samples were resolved at 100 V for 2 to 3 hours. Proteins were transferred to polyvinylidene difluoride membranes at 100 V for 2 hours at 4°C and immediately incubated for 1 hour at RT in BSA blocking buffer 3% BSA in 1× TBS-T [20 mM tris buffer (pH 8.8), 150 mM NaCl, and 0.1% tween 20]. The membranes were incubated with a primary anti–1-*P*-histidine antibody (clone SC1-1 1/1000 in TBS-T) overnight at 4°C. After incubation with horseradish peroxidase–conjugated secondary antibodies, the membranes were washed three times for 10 min each with 0.1% TBS-T. The revelation of the membranes was performed using the Clarity Western ECL Blotting Substrate (Bio-Rad, 1705060) following the usual procedure.

### Native PAGE

NME1 (24 μM) was incubated for 10 min at RT with ATP (from 0 to 384 μM) and phosphorylated NME1 (previously prepared by incubating with 192 μM ATP) was incubated with ADP (from 192 to 1728 μM) in TMD buffer for 10 min at RT. After adding 2 μl of native loading buffer [62.8 mM tris-HCl (pH 6.8), 40% glycerol, and 0.01% bromophenol blue], 5 μl of samples was analyzed on a 10% TGX (Bio-Rad) gel and run under native conditions (0.5× TBE buffer, 4°C, 100 V, 120 min). Gels were stained with Coomassie blue and scanned on a ChemiDoc MP gel imaging system (Bio-Rad).

### In vitro CoA binding assay of the NME1/2 proteins

CoA pull-down was performed in the presence of purified WT NME1 or the T94D mutant. Three micrograms of WT or mutant GST-tagged or untagged NME1 was incubated overnight at 4°C (300 μl final volume) with 50 μl of pre-washed CoA-agarose resin (buffer: 20% glycerol, 3 mM MgCl_2_, 50 mM Hepes, 500 mM KCl, 1 mM DTT, and one mini-tablet of EDTA-free protease inhibitor) in the presence or absence of ATP (Sigma-Aldrich). The enriched CoA-agarose was washed three times in the buffer and, after a final wash with PBS, was incubated for 1 hour at 4°C in 50 μl of 5 mM free ligand (either CoA, NAD, FAD, ADP-ribose, ATP, or dephospho-CoA; all from Sigma-Aldrich) or 50 μl of H_2_O as a control. Flow-through and CoA-agarose (eluted by free CoA) fractions were analyzed by WB to detect NME1 (by an anti-GST or anti-NME2 antibody) as well as histidine phosphorylation. GST-NME1 and GST-NME2 were also pulled down using extracts from bacteria expressing these proteins, following the same protocol as the CoA pull-down from eukaryotic cell extracts.

### Determination of NDPK activity

NME kinase activity was determined spectrophotometrically with 4 mM ATP and 1 mM thymidine 5′-diphosphate as substrates using a coupled enzyme assay. ADP production was coupled by pyruvate kinase (160 U/ml) and lactate dehydrogenase (800 U/ml) to NADH (reduced form of NAD^+^) oxidation using 4.5 mM Mg-acetate, 0.9 mM phosphoenolpyruvate, and 0.45 mM NADH in 0.1 M triethanolamine buffer (pH 7). The assay was started by the addition of NME (20 ng of WT or 7 μg of mutant/1 ml), and changes in NADH redox state were followed at 340 nm and 25°C for 10 min. NME kinase activity was then inhibited with 1 mM AcCoA and followed for another 10 min. Data were corrected for nonspecific inhibitory effects of AcCoA on the coupled enzyme system by running controls with creatine kinase (4 mM ATP and 20 mM creatine as substrates) with the same protocol.

### NME1 and ATP-dependent nucleosome acetylation

Equimolar amounts of purified NME1 and AcCoA (49 μM) were preincubated in HAT buffer [0.1 mM sodium phosphate (pH 7.4), 6 mM MgCl_2_, 1 mM DTT, and 1× Complete EDTA-free protease inhibitor (Roche)] for 30 min at 4°C before initiation of the reaction (30 min at RT) by adding pure mononucleosome (0.4 μg/μl), FLAG-P300 (0.2 μM)/FLAG-GCN5 (0.6 μM), and ATP (50 μM) in 13 μl of the final volume. Seven microliters of the reaction was quenched by adding 3 μl of 4× Laemmli buffer, followed by analysis on a 4 to 20% SDS-PAGE gel. WB was performed with anti-FLAG, H4K5ac, H3K14ac, and H4 antibodies.

### Fatty acid detection

Total fatty acids were extracted from the liver using a 1:2 (v/v) mixture of chloroform/methanol in the presence of 10 nmol of tridecanoic acid (Sigma-Aldrich no. T0502) and 10 nmol of phosphatidylcholine (C21:0/C21:0, Avanti no. 850370) as an internal standard. The liver samples were dissected into small pieces and ground in methanol before the addition of chloroform. After vigorous sonication and vortexing, the organic phase was extracted by biphasic separation generated by the addition of chloroform and 0.2% KCl to obtain a chloroform/methanol/aqueous ratio of 2:1:0.8 (v/v/v). The resulting bottom organic phase was dried and on-line derivatized to fatty acid methyl ester by a chloroform/methanol (1:2)-trimethylsulfonium hydroxide (TMSH) solution (Macherey-Nagel) and analyzed by gas chromatography-MS (Agilent, 5977A-7890B). The abundance of each fatty acid was calculated and normalized according to the internal standard and cell number or wet weight.

### HFD treatment

The HFD treatment was approved by the official ethics committee of the University Grenoble Alpes (ComEth, C2EA-12). WT and Nme2 ko mice [C57BL/6N-Nme2tm1.1(KOMP)Vlcg/MbpMmucd; MMRRC stock no.: 048819-UCD] were fed with ND (Safe A03, France) and high-fat custom diet (SAFE U8978, version 19) for 6 weeks. The food and mice were weighed every 3 to 4 days.

### Detection of adenine nucleotides, CoA, and AcCoAs in liver

Adenine nucleotides, CoA, and AcCoAs were determined in protein-free extracts obtained by perchloric acid precipitation as follows. Freeze-clamped livers were homogenized in 0.5 N perchloric acid (2:1 v/w) in liquid nitrogen. After thawing at RT, 4 N perchloric acid (1:10 v/w) was added, and the homogenate was incubated on ice for 30 min and centrifuged (4000*g*, 5 min, 4°C). The supernatant was neutralized by the addition of 5 M K_2_CO_3_ and centrifuged again (4000*g*, 5 min, 4°C). The metabolites were determined by high-performance liquid chromatography (Varian 410, France) with RP-C18 column (Polaris C18-A, 250 × 4.6, 5 μm, Varian, France; ref. A2000250R046) at a 1 ml/min flow rate at 30°C, as described in detail elsewhere (*63*, *64*). Briefly, to determine adenine nucleotides, the protein-free extract (75 ml premixed with 50 ml mobile phase) was separated in pyrophosphate buffer (28 mM, pH 5.75), and the detection was performed at 254 nm. The ATP, ADP, and AMP were eluted at 6.5, 8, and 14 min, respectively. To determine CoA and AcCoA, the protein-free extract (75 ml premixed with 50 ml mobile phase) complemented with DTT (2 mM final) was separated in 100 mM monosodium phosphate and 75 mM sodium acetate (pH 4.6) combined with acetonitrile in the proportion 94:6 (v/v). The detection was performed at 259 nm. The CoA and AcCoA were respectively eluted at 7 and 16 min. The elution peaks were integrated using the STAR software (Varian, France).

### Measurement of liver triglyceride content

Frozen liver fragments (50 mg) were digested in 0.15 ml of 3 M alcoholic potassium hydroxide (70°C, 2 hours), and the amount of liver triglycerides was measured using a triglycerides kit (Erba Mannheim, Brno, Czech Republic, ref: BLT00059, lot 2107029); spectroscopy was used to measure the samples’ absorbance.

### Histological analyses

The liver tissues were fixed in a formalin solution, neutral-buffered at 10% (Sigma-Aldrich, Steinheim am Albuch, Germany), and paraffin-embedded. Four-micrometer sections of tissue were prepared. Hematoxylin and eosin staining was used for histopathological examination. The presence of steatosis was determined through a blind evaluation of the slides.

### Antibody dilutions for WB

Antibody dilutions for WB are as follows: Anti Nme2, 1/5000; Acc1, 1/1000; P-Acc1, 1/1000; AMPKα, 1/1000; P-AMPKα, 1/1000; glyceraldehyde-3-phosphate dehydrogenase, 1/1000; β-tubulin, 1/2000; α-tubulin, 1/2000; β-actin, 1/2000; Histidine 1P (SC1-1 clone), 1/1000, H3K9ac, 1/1000; and H3, 1/5000.

### Supplementary materials and methods

Experimental procedures concering MS-based proteomic analyses, crystallization and crystal structure determination, nanoDSF, ITC, LC/ESI MS, native MS, transcriptome, and ChIP-seq experiments and analyses are detailed in the Supplementary Materials. All the resources used—reagents, antibodies, kits, and PCR primers—are presented as a resource table (table S5).

### Quantification and statistical analysis

MS-based proteomics of the CoA-bound proteins (Fig. 1, C to E, and fig. S1): Four testes from two adult male mice were the biological source of total testis CoA–bound proteins, while 12 testes from six adult males were the source of fractionated spermatogenic cells CoA-bound proteins.

NME1/2 WB in liver (Fig. 5A): (2-month-old mice); 2 *Nme2^+/+^* [2 females (F)], *2 Nme2^−/−^* (2 F).

CoA pull-down liver-silver staining: (Fig. 5B): (2- to 3-month-old mice); 3 *Nme2^+/+^* [3 males (M)], 3 *Nme2*^−/−^ (3 M).

Fatty acid detection (Fig. 5, C and D): The graphs show the average values and SD. Liver from adult mice (2 to 3 months); 5 *Nme2^+/+^* (2 F, 3 M), 4 *Nme2^−/−^* (2 F, 2 M).

Steatosis in liver (Fig. 5E): (2- to 4-month-old mice); 4 *Nme2^+/+^* in ND (2 F, 2 M)—Steatosis was not detected in these liver samples; 5 *Nme2^−/−^* in ND (2 F, 3 M), no steatosis; 6 *Nme2^+/+^* in HFD (1 F, 5 M), no steatosis; 7 *Nme2^−/−^* in HFD mice (3 F, 4 M)—Steatosis was detected in 4 M, and mild steatosis in 3 F.

Triglyceride detection in liver (Fig. 5F): (3- to-5-month mice); 4 *Nme2^+/+^* (2 F, 2 M) in ND, 4 *Nme2^−/−^* (2 F, 2 M) in ND, 10 *Nme2^+/+^* (3 F, 7 M) in HFD, and 12 *Nme2^−/−^* (5 F, 7 M) in HFD. The graph shows the average values and SD.

RNA-seq (Fig. 6A): (2- to 4-month-old mice), 4 *Nme2^+/+^* in ND (4 M), 4 *Nme2^+/+^* in HFD (4 M), 4 *Nme2^−/−^* in ND (4 M), and 4 *Nme2^−/−^* in HFD (4 M).

CoA and AcetylCoA detection in liver (fig. S12C): (3- to 5-month-old mice), 9 *Nme2^+/+^* in ND (2 F, 7 M), 4 *Nme2^+/+^* in HFD (2 F, 2 M), 9 *Nme2^−/−^* in ND (2 F, 7 M), and 5 *Nme2^−/−^* in HFD (2 F, 3 M). The graph shows the average values and SD. Student’s *t* test was used to calculate the *P* values for HFD versus ND in all conditions.

Chip-seq and H3K9ac WB (Fig. 7A and fig. S12B): (3- to 5-month-old mice), 2 *Nme2^+/+^* in ND (2 M), 2 *Nme2^+/+^* in HFD (2 M), 2 *Nme2^−/−^* in ND (2 M), and 4 *Nme2^−/−^* in HFD (2 M).

WB in liver (fig. S13A): (2- to 4-month-old mice); 3 *Nme2^+/+^* in ND (3 M), 3 *Nme2^−/−^* in ND (3 M), 4 *Nme2^+/+^* in HFD (4 M), and 4 *Nme2^−/−^* in HFD (4 M).

ATP/ADP/AMP detection in liver (fig. S13B): (3- to 5-month-old mice), 9 *Nme2^+/+^* in ND (2 F, 7 M), 4 *Nme2^+/+^* in HFD (2 F, 2 M), 9 *Nme2^−/−^* in ND (2 F, 7 M), and 5 *Nme2^−/−^* in HFD (2 F, 3 M). The graph shows the average values and SD.

In all cases, the statistical significance is marked with the symbols *, **, and *** for *P* values <0.05, <0.01, and <0.001, respectively.

## Supplementary Materials

### This PDF file includes:

Supplementary Materials and Methods Figs. S1 to S13 Legend for table S1 Tables S2 to S5 Legends for data S1 and S2 References
