# Supplementary material for: Nucleoside diphosphate kinases 1 and 2 regulate a protective liver response to a high-fat diet
Source: Sci Adv. 2023 Sep 6;9(36):eadh0140. doi: 10.1126/sciadv.adh0140 (PMC10482350; doi:10.1126/sciadv.adh0140)
Supplement: Supplementary file 1 — Supplementary Materials and Methods Figs. S1 to S13 Legend for table S1 Tables S2 to S5 Legends for data S1 and S2 References [file sciadv.adh0140_sm.pdf]

Science Advances
AAAS

Supplementary Materials for

Nucleoside diphosphate kinases 1 and 2 regulate a protective liver response to a high-fat diet

Domenico Iuso *et al.*

Corresponding author: Carlo Petosa, carlo.petosa@ibs.fr; Saadi Khochbin, saadi.khochbin@univ-grenoble-alpes.fr

Sci. Adv. **9**, eadh0140 (2023)
DOI: 10.1126/sciadv.adh0140

The PDF file includes:

Supplementary Materials and Methods
Figs. S1 to S13
Legend for table S1
Tables S2 to S5
Legends for data S1 and S2
References

Other Supplementary Material for this manuscript includes the following:

Table S1
Data S1 and S2

Supplementary Materials and Methods

Mass spectrometry (MS)-based proteomic analyses of the CoA bound proteins

The eluted proteins solubilized in Laemmli buffer were stacked in the top of a 4-12% NuPAGE gel (Invitrogen). After staining with R-250 Coomassie Blue (Biorad), the proteins were digested in-gel using trypsin (modified, sequencing purity, Promega), as previously described (*66*). The resulting peptides were analyzed by online nanoliquid chromatography coupled to MS/MS (Ultimate 3000 and LTQ-Orbitrap Velos Pro, Thermo Fisher Scientific) using a 124 min gradient. For this purpose, the peptides were sampled on a precolumn (300 µm x 5 mm PepMap C18, Thermo Scientific) and separated in a 75 µm x 250 mm C18 column (PepMap C18, 3 µm, Thermo Fisher Scientific). The MS and MS/MS data were acquired by Xcalibur (Thermo Fisher Scientific).

The peptides and proteins were identified by Mascot (version 2.7.0, Matrix Science) through concomitant searches against the Uniprot database (*Mus musculus* taxonomy, June 2021 download), and a homemade classical database containing the sequences of classical contaminant proteins found in proteomic analyses (human keratins, trypsin, etc.). Trypsin/P was chosen as the enzyme and two missed cleavages were allowed. Precursor and fragment mass error tolerances were set respectively at 10 ppm and 0.6 Da. Peptide modifications allowed during the search were: Carbamidomethyl (C, fixed), Acetyl (Protein N-term, variable) and Oxidation (M, variable). The Proline software (*67*) was used for the compilation, grouping, and filtering of the results (conservation of rank 1 peptides, peptide length ≥ 6 amino acids, false discovery rate of peptide-spectrum-match identifications < 1%, and minimum of one specific peptide per identified protein group (*68*). Proline was then used to perform MS1-based label free quantification of the identified protein groups based on razor and specific peptides. The proteins from the contaminant database were discarded from the final list of identified proteins. To calculate fold changes between CoA and control eluates, missing values were imputed in ProStaR (*69*) using the DetQuantile algorithm (imputation with a value corresponding to the first percentile of the sample). To be considered as a potential CoA interactor, a protein must be quantified with a minimum of two peptides and be enriched at least 10 times in the CoA eluates compared to the control eluate. The relative abundance of the different proteins in each sample was evaluated through calculation of their intensity-based absolute quantification (iBAQ) (*57*) values.

Crystallization and crystal structure determination

Protein crystallization was performed by the hanging drop vapor diffusion method at 20°C (for ADP- and CoA-bound NME1) or 4°C (for SucCoA-bound NME1) by mixing 1 µl of the NME1/ligand complex with 1 µl of reservoir solution. ADP-bound NME1 was crystallized by mixing a solution of 6.1 mg/ml NME1, 1 mM ADP, 250 mM NaCl and 25 mM Tris pH 7.4 with 0.2 M NaCl, 0.1 M Tris pH 8.0 and 30% (w/v) PEG 400. CoA-bound NME1 was crystallized by mixing a solution of 5.5 mg/ml NME1, 1 mM CoA, 150 mM NaCl and 0.1 M phosphate buffer pH 7.4 with 0.2 M $Mg(NO_3)_2$ and 36% PEG 3350. SucCoA-bound NME1 was crystallized by mixing a solution of 5.5 mg/ml NME1, 1 mM SucCoA, 150 mM NaCl and 0.1 M phosphate buffer pH 7.4 with 0.1 M citric acid pH 3.5 and 4-10% PEG 1000. Harvested crystals were flash-cooled in liquid nitrogen.

Diffraction data were collected at beamline ID30A-1 of the European Synchrotron Radiation Facility (ESRF). All crystals were monoclinic with space group $P2_1$ and contained either one (ADP, CoA) or two (SucCoA) NME1 hexamers in the asymmetric unit. Data collection statistics are summarized in table S2. Data for ADP- and SucCoA-bound NME1 were automatically integrated with XDS (*70*) which is included in the automatic software toolbox auto-PROC (*71*) and scaled with AIMLESS (*72*). Data for CoA-bound NME1 was manually integrated with XDS and scaled with AIMLESS. Structures were solved by molecular replacement in Phaser (*73*) using the structure of human NME1 bound to ADP (PDB 2HVD) (*26*) or imidazole fluorosulfate (PDB 5UI4) (*74*) after removing ligands and water molecules, as search models. Iterative rounds of refinement and model building were performed using PHENIX (*75*) and Coot (*76*), resulting in the final R-values shown in table S2. In the ADP-bound structure, ADP is observed bound to 5 of the 6 subunits in the NME1 hexamer, whereas the remaining binding site is empty because of crystal packing constraints. For CoA- and SucCoA-bound NME1 all ligand-binding sites are occupied. Weak or missing electron density precluded reliable modeling of the CoA/SucCoA pantetheine moiety and the SucCoA succinyl group, which are consequently omitted from the final refined atomic coordinates.

Nano-differential scanning fluorimetry (nano-DSF)

10 µL samples of 0.5 mM NME1 prepared in the absence or presence of either CoA, AcCoA, SucCoA or ADP at a concentration of 0.62 mM in a buffer containing 25 mM Tris pH 7.4, 250 mM NaCl were loaded into nanoDSF Grade Standard Capillaries (NanoTemper, #PR-C002) and analysed on a Prometheus NT 48 instrument (Nanotemper). Assay samples were heated from

20°C to 95°C at a rate of 0.5°C/min. Intrinsic tryptophan fluorescence was measured at 330 nm and 350 nm using an excitation power of 10%. The 350/330 fluorescence ratio was plotted against temperature and the T_m value determined from the inflection point using the instrument software.

Isothermal titration calorimetry (ITC)

Calorimetric experiments were performed in triplicate on a MicroCal iTC200 calorimeter (Malvern Panalytical) at 20°C while stirring at 330 rpm. The syringe and cell were respectively filled with 900 µM CoA and 30 µM NME1, both in 0.1 M phosphate buffer pH 7.4, 150 mM NaCl. Titrations consisted of 50 identical injections of 6 µl made at time intervals of 450 s. ITC data were corrected for the heating of CoA injection into buffer and analyzed with the MicroCal PEAQ-ITC Analysis Software (Malvern Panalytical).

LC/ESI mass spectrometry

Liquid Chromatography Electrospray Ionization Mass Spectrometry (LC/ESI-MS) was performed on a 6210 LC-TOF spectrometer coupled to an HPLC system (Agilent Technologies). All solvents used were HPLC grade (Chromasolv, Sigma-Aldrich). Trifluoroacetic acid (TFA) was from Acros Organics (puriss., p.a.). Solvent A was 0.03% TFA in water, solvent B was 95% acetonitrile-5% water-0.03% TFA. Just before analysis NME1 samples (57 mM in sample buffer: 20 mM Tris-HCl pH 8.8, 5 mM $MgCl_2$, 1 mM DTT) were diluted with sample buffer to a final concentration of 5.7 µM and 4 ml were injected for MS analysis. Protein samples were first desalted on a reverse phase-C8 cartridge (Zorbax 300SB-C8, 5 mm, 300 µm ID´5mm, Agilent Technologies) for 3 min at a flow rate of 50 ml/min with 100% solvent A and then eluted with 70% solvent B at a flow rate of 50 ml/min for MS detection. MS acquisition was carried out in positive ion mode in the 300-3200 *m/z* range. MS spectra were acquired and the data processed with MassHunter workstation software (v. B.02.00, Agilent Technologies) and with GPMAW software (v. 7.00b2, Lighthouse Data, Denmark).

Native mass spectrometry

Mass Spectrometry under native conditions was performed to detect NME1 species following incubation with either CoA, dephospho-CoA, AcCoA, SucCoA, or a mixture of both CoA and ATP (all from Sigma-Aldrich). NME1 (50 µM) was incubated with the ligands (5 to 2500 µM) in a buffer containing 250 mM ammonium acetate, 0.5 mM magnesium acetate and DTT 1 mM for 15 min

at room temperature and subsequently loaded by a nanoflow platinum-coated borosilicate electrospary capillary (Thermo Electron SAS; Courtaboeuf, France) into a quadrupole time-of-flight mass spectrometer (nano-ESI-Q-TOF instrument, Q-TOF Ultima, Waters Corporation, Manchester, U.K.). The following instrumental parameters were used: capillary voltage = 1.2-1.3 kV, cone potential = 40 V, RF lens-1 potential = 40 V, RF lens-2 potential = 1 V, aperture-1 potential = 0 V, collision energy = 30-140 V, and microchannel plate (MCP) = 1900 V. The pressure in the collision cell was set to ~2 × 10^{-2} mbar. All mass spectra were calibrated externally using a solution of cesium iodide (6 mg/mL in 50% isopropanol) and were processed with the Masslynx 4.0 software (Waters Corporation, Manchester, U.K.) and with Massign software package.

Transcriptome (RNAseq)

RNAseq overall design

Transcriptomic analysis was performed on mouse liver samples obtained from 4 conditions, corresponding to two genotypes, Nme2 WT ($Nme2^{+/+}$) and KO ($Nme2^{-/-}$), and two experimental diets, normal diet (ND) and high fat diet (HFD). For each condition, RNAseq was performed in 4 replicates corresponding to 4 individual mice.

Extraction protocol

Total RNA was isolated and purified from the liver tissue by NucleoSpin RNA kit (Machery-Nagel-740955.50). Four independent RNA extractions were performed for each condition.

Library construction protocol

For each sample, one μg of RNA (RIN>8) was used for library preparation with the Illumina Stranded total RNA Prep Ligation with Ribo-Zero Plus kit (Illumina) according to the manufacturer's instructions. Each library was quantified on Qubit with the Qubit® dsDNA HS Assay Kit (Life Technologies) and the size distribution was examined on the Fragment Analyzer with High Sensitivity NGS Fragment Analysis kit (Agilent).

Library strategy

The libraries were then sequenced on the Illumina NextSeq 500 (paired-end 75) at the TGML Platform of Aix-Marseille University (France).

Data Processing Pipeline

Alignment

The sequenced reads from the raw sequence (.fastq files) were aligned on the UCSC mm10 genome using the STAR software (2.7.1a) (*77*) to produce bam files.

Counts

The aligned reads (.bam files) were counted using HTSeq framework (0.11.2)) (*78*) with options: -t exon -f bam -r pos –stranded=reverse -m intersection-strict –nonunique none.

Normalization and differential analysis

The normalization, pseudo-log transformation of the read counts, and the differential analyses were performed using the R software (*80*), DESeq2 (1.22.2) (*81, 82*) and SARTools (*83*) packages.

Grouping genes/features according to their quartile of expression

The features corresponding to non-coding transcripts and/or with zero counts were filtered out, in order to retain all non-zero count protein-coding genes from the NCBI Reference Sequence Database (RefSeq). The corresponding genes and features were ranked according to their DESeq2 normalized expression mean value in the samples corresponding to the 4 replicates of liver from wild-type mice under normal diet, and allocated into four subsets corresponding to the quartiles of this expression and exported as .bed files.

Identification of up- and down-regulated genes and selection for heatmap representation

Supervised transcriptomic analyses were performed to identify genes significantly up- and down-regulated between two conditions using thresholds of a Student *t*-test *p*-value <0.01 and a fold change absolute value of 2.

The normalized, pseudo-log transformed and standardized read counts of the up- and down-regulated genes in wild type mice between those submitted to a high fat diet (HFD) and those with a normal diet (ND) were used to generate the heatmap presented in Fig. 6A.

ChIPseq sequencing and analysis

H3K9ac ChIP assay

ChIP assays for H3K9ac were carried out as previously described with minor modifications (*60, 61*). Briefly, 50 µl of Dynabeads protein G (Thermo-fisher 10004D) were washed 3 times with blocking solution (5% BSA/PBS) and incubated in 500 µl overnight at 4°C with 5 µl of H3K9ac antibody. On the second day, liver tissue was homogenized in cold buffer (3 ml/each liver) containing 2.2 M sucrose, 10 mM Tris-HCl pH 7.5, 10 mM $MgCl_2$, 10 mM sodium butyrate and protease inhibitor, filtered by hydrophilic gauze and centrifuged for 3 h at 100,000 g at 4°C. Nuclei in the pellet were washed in a buffer containing 0.5 M sucrose, 10 mM Tris-HCl pH 7.5, 10 mM $MgCl_2$, 0.2% Triton X-100, 10 mM sodium butyrate, protease inhibitor, centrifuged for 1467 g at 4°C and resuspended in a buffer containing 1 M sucrose, 10 mM Tris-HCl, 10 mM $MgCl_2$, 10 mM sodium butyrate (Sigma-Aldrich B-2503) and protease inhibitor. 45 µg of nuclei were digested

with 0.75 µg of Nuclease S7 (Sigma-Aldrich, Cat#10107921001) for 15 min at 37 °C in 100 µl of a buffer containing 20 mM of Tris-HCl pH7.5, 5 mM $CaCl_2$ to obtain mononucleosomes (146 bp). The reaction was stopped by adding 5 mM EDTA. Small aliquots of mononucleosome solutions were collected for input. Digested mononucleosomes were diluted with LSDB buffer (50 mM HEPES pH 7.0, 3 mM $MgCl_2$, 500 mM KCl, 20% glycerol, protease cocktail inhibitor containing 10 mM sodium butyrate) to achieve the final KCl concentration of 350 mM and incubated with antibody-coupled beads overnight at 4°C for 16 hours. On the third day, the beads were washed four times with LSDB 350 mM KCl and once in TE buffer (10 mM Tris-HCl containing 1 mM EDTA, pH 8.0). ChIP samples were eluted in 150 µl of the buffer with SDS 1% for 20 min at 65°C. The DNA was purified from the eluted ChIP samples as well as from the input samples by phenol-chloroform extraction and ethanol precipitation.

ChIPseq overall design

ChIPseq analysis was performed in mouse liver samples obtained from 4 conditions, corresponding to two genotypes, Nme2 WT ($Nme2^{+/+}$) and KO ($Nme2^{-/-}$), and two experimental diets, normal diet (ND) and high fat diet (HFD). For each condition, chromatin immunoprecipitation was performed twice in the respective livers of two independent mice using anti-H3K9ac antibody of mNase digested chromatin and both the immune-precipitated (chip) and input materials were sequenced.

Extraction protocol

As previously reported, the DNA was purified from the eluted ChIP samples as well as from the input samples by phenol-chloroform extraction and ethanol precipitation.

Library construction protocol

For sequencing, ChIP libraries were prepared using MicroPlex Library Preparation Kit v3 (Diagenode) according to manufacturer's instructions. Each library was quantified on Qubit with Qubit® dsDNA HS Assay Kit (Life Technologies) and size distribution was examined on the Fragment Analyzer with High Sensitivity NGS Fragment Analysis kit (Agilent).

Library strategy

The ChIP libraries were sequenced on a High-output flow cell (400M clusters) using the NextSeq® 500/550 High Output v2.5 150 cycles kit (Illumina), in paired-end 75/75nt mode, according to manufacturer's instructions at the TGML Platform of Aix-Marseille University (France). Base calling was performed using RTA version 2.*

Data Processing Pipeline

Data processing step: Trimming

The raw fastq files were processed by 5 prime trimming, keeping 30bp-length fragments, using fastx_trimmer [http://hannonlab.cshl.edu/fastx_toolkit/. Accessed 28 Feb. 2022.], with options -l 30 -Q33.

Data processing step: Alignment

The trimmed fastq files were aligned on the UCSC Mus_musculus mm10 genome using the Bowtie2 aligner (*83*), with options –end-to-end, –no-mixed, –no-discordant.

Genome build: UCSC Mus_musculus mm10 genome

Processed data files format and content: big wig files (.bw) containing normalized integrated aligned read count signals.

Selection of features corresponding to TSS and normalization

Raw ChIP counts corresponding to TSS +1500/-800bp of the RefSeq genes were computed using featureCounts (*84*), with option -a mm10_pc_tss8001500.saf -F SAF -s 0 -Q 30 -T 8 -o mm10_pc_tss8001500_count_30.txt.

For each sample, scale factors were computed on these features using DESeq2 (*80, 81*) assuming that the global level of ChIP signal value (corresponding lysine acetylation in all genes promoters) should be equal in all samples.

The scale factors are given in the "Deseq2_sf " table shown below.

sample	sf	inv_sf	bw_file
normal_chip_nme2wt_m301	1	1,13	normal_chip_nme2wt_m301_end-to-end_trim30_srt_mmq30_PESF_30_4_None_SF1.13.bw
normal_chip_nme2wt_m302	1	1,48	normal_chip_nme2wt_m302_end-to-end_trim30_srt_mmq30_PESF_30_4_None_SF1.48.bw
hghfat_chip_nme2wt_m300	1	0,7	hghfat_chip_nme2wt_m300_end-to-end_trim30_srt_mmq30_PESF_30_4_None_SF0.70.bw
hghfat_chip_nme2wt_m303	1	0,77	hghfat_chip_nme2wt_m303_end-to-end_trim30_srt_mmq30_PESF_30_4_None_SF0.77.bw
normal_chip_nme2ko_m291	1	1,31	normal_chip_nme2ko_m291_end-to-end_trim30_srt_mmq30_PESF_30_4_None_SF1.31.bw
normal_chip_nme2ko_m293	1	1,08	normal_chip_nme2ko_m293_end-to-end_trim30_srt_mmq30_PESF_30_4_None_SF1.08.bw
hghfat_chip_nme2ko_m273	1	0,95	hghfat_chip_nme2ko_m273_end-to-end_trim30_srt_mmq30_PESF_30_4_None_SF0.95.bw
hghfat_chip_nme2ko_m292	1	0,79	hghfat_chip_nme2ko_m292_end-to-end_trim30_srt_mmq30_PESF_30_4_None_SF0.79.bw

The bam signals were normalized and smoothed using bamCoverage (from deepTools suite (*85*)) with options: –binSize 4 –minMappingQuality 30 –normalizeUsing None –scaleFactor inv_sf where inv_sf is the inverse of the scaling factor previously computed.

ChIPseq Heatmaps

Repeat masker tagged loci GSAT_MM and SYNREP_MM as well as the Sfi1 locus (chr11:3126500-3200500) and mitochondrial (chrM) genes were excluded from the analysis.

The normalized ChIP signals were converted into a 10bp bin matrix of the signal 1.5Kb upstream and downstream of protein-coding genes TSS, using computeMatrix (from deepTools suite (*85*)),

with options reference-point –referencePoint TSS –binSize 10 –beforeRegionStartLength 1500 –afterRegionStartLength 1500 –sortRegions descend

Heatmaps were generated using plotHeatmap (deepTools suite (*85*) with options –colorMap YlOrRd –sortRegions descend).

Highly expressed gene TSS profiles

The normalized ChIP signal were converted into a 10bp bin matrix of the signal 1.5Kb upstream and downstream 25% more expressed (see Mat & Meth RNA-seq) protein-coding genes TSS, using computeMatrix.

The ChIPseq profiles were generated using the computed computeMatrix outputs and a custom R script (*79*).

Expression vs. Acetylation

The normalized ChIP signals were converted into a 25 bp bin matrix of the signal 750 bp upstream and 1500 bp downstream protein-coding genes TSS, using computeMatrix.

For each gene and condition the mean signal over this region in all replicates was plotted.

In Fig. 7C, the *x*-axis represents the $\log_2$ (fold changes) of the differential expression (DESeq2 normalized values) between HFD and ND respectively for Nme2 WT (upper panel) and KO (lower panel).

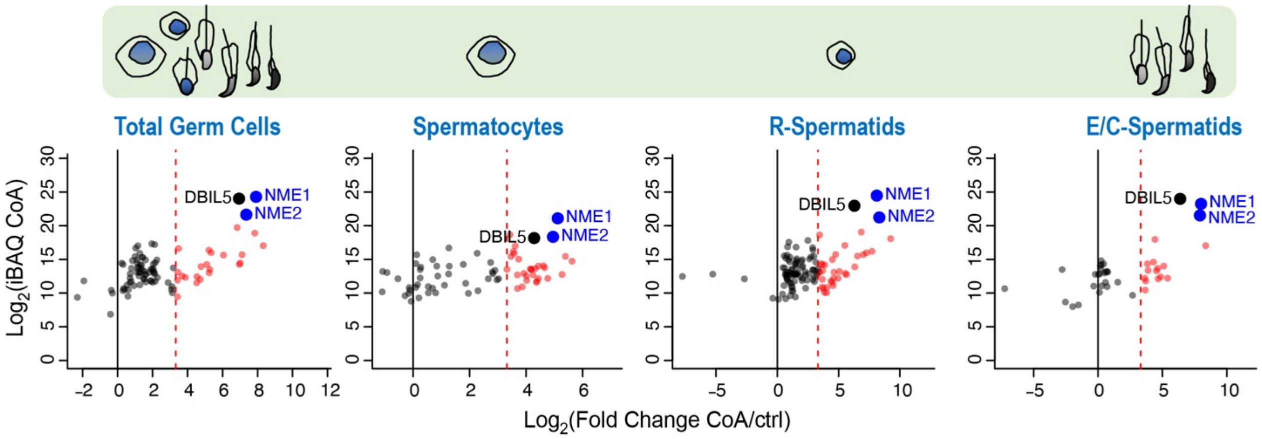

Figure S1. NME1/2 are major CoA-binding factors in mouse spermatogenic cells. CoA-pull downs were performed in the presence of 500 mM KCl on extracts from total germ cells, pachytene spermatocytes, round spermatids, and elongating and condensing spermatids.

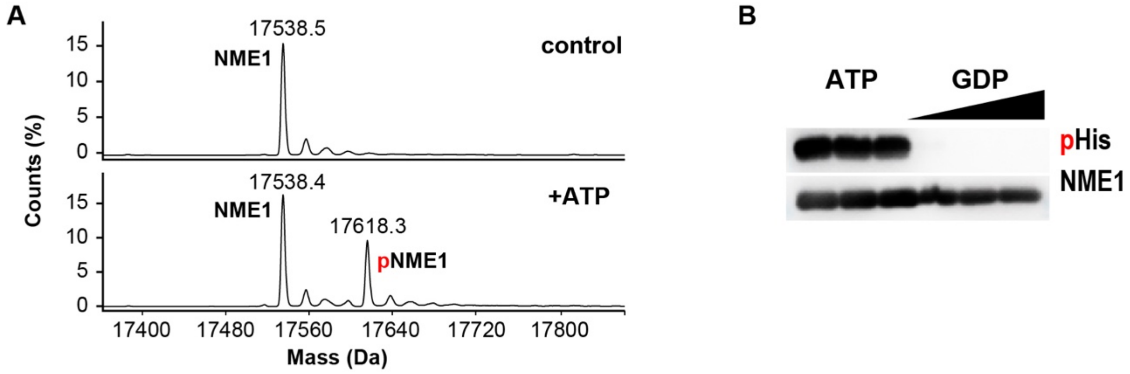

Figure S2. Recombinant NME1 is phosphorylated by ATP and dephosphorylated by GDP.
(**A**) MS analysis of NME1 incubated without (top) or with (bottom) ATP. Under the experimental conditions used (5.7 μM NME1, 100 μM ATP) approximately 40% of NME1 appears to be phosphorylated.
(**B**) NDPK activity confirmed by an immunoblot. An anti-phosphohistidine antibody detects phosphorylated recombinant NME1 (0.23 mM incubated with 0.5 mM ATP in triplicate). The phosphohistidine signal is lost after the incubation of phosphorylated NME1 (0.028 mM) with GDP (0.1, 0.2 and 0.4 mM).

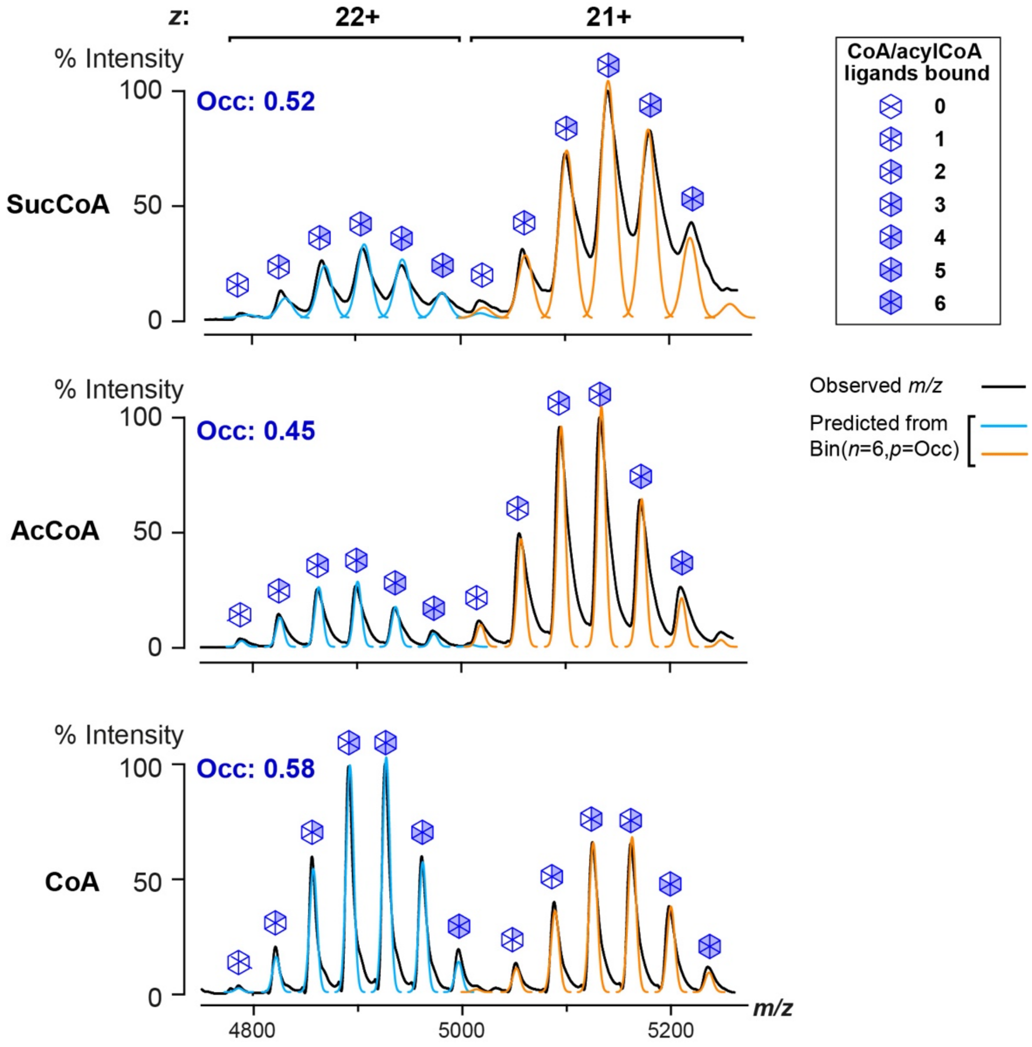

Figure S3. Native MS spectra reveal a binomial distribution of CoA/acyl-CoA-bound hexameric NME1 species. Spectra shown are the same as in Fig. 2C. Peaks in the spectra were modelled as a simple Gaussian function whose amplitude was scaled by the values given by the binomial distribution, Bin(N,p), where N is the number of binding sites on the NME1 hexamer (N=6) and p is the fitted probability of a single site being occupied by a ligand. The fraction of NME1 hexamers bound to r ligands (r = 0 to 6) is given by C(6,r)p^r(1-p)$^{1-r}$ where C(6,r)= 6!/[r!(6-r)!]. Recorded spectra are shown in black and fitted curves for the 22+ and 21+ charge states are in cyan and orange, respectively. The good agreement between the recorded spectra and the fitted curves confirms that CoA/acylCoA ligands bind independently to the six binding sites on each NME1 hexamer, allowing one to estimate the fraction (*Occ*, equal to p) of NME1 monomers bound to each ligand, which for these experiments ranged between 0.45 and 0.58.

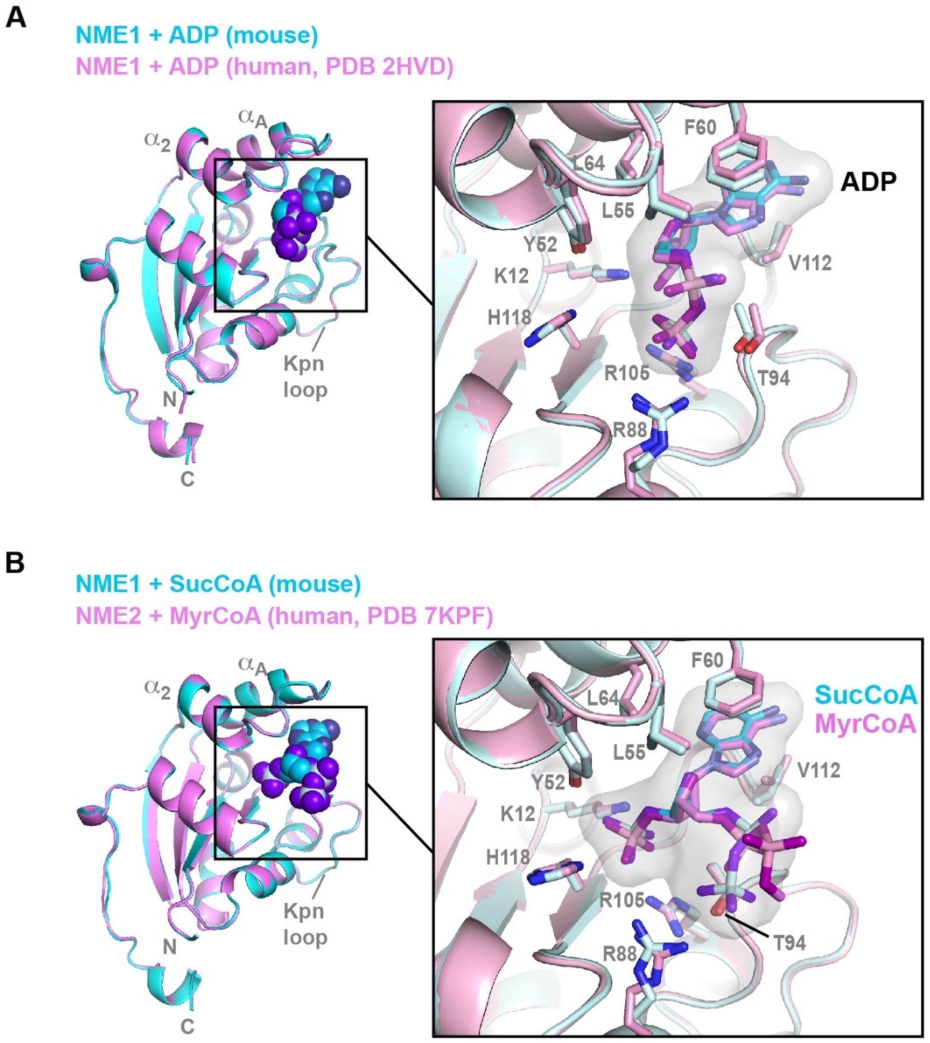

Figure S4. Similarity with previously reported NME1/2 structures.

(**A**) Alignment of ADP-bound structures of murine NME1 and human NME1 (PDB 2HVD) (*26*).

(**B**) Structural alignment of murine NME1 bound to SucCoA with human NME2 bound to myristoyl-CoA (PDB 7KPF)(*15*).

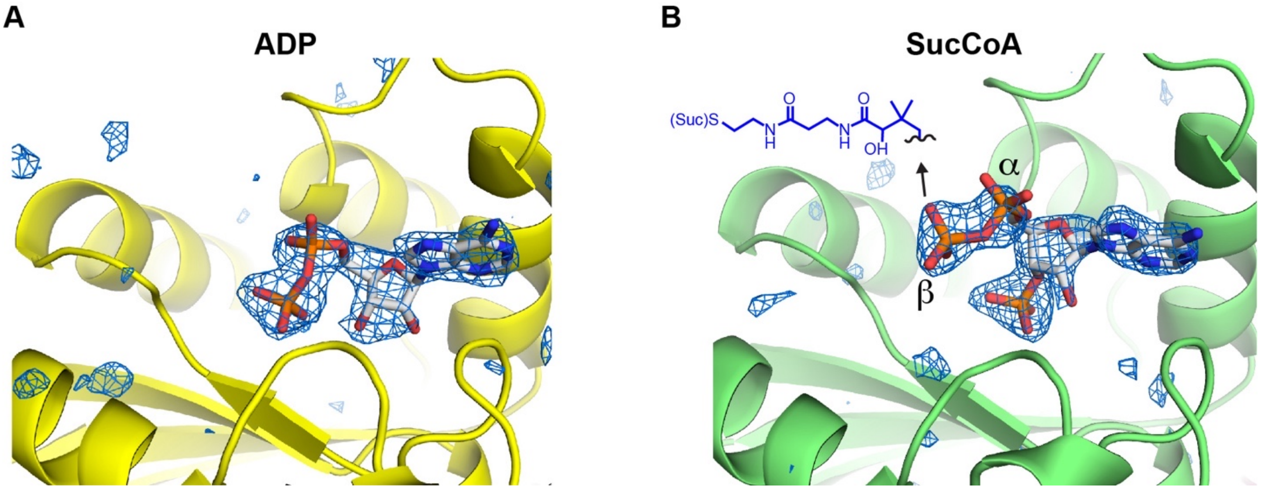

Figure S5. Omit map density for (A) ADP and (B) SucCoA bound to NME1. The electron density (mesh in blue) shows F_o-F_c omit maps contoured at 3.0σ (ADP) and 2.0σ (SucCoA) where ligands were omitted from the map calculation. No strong density is observed for the pantetheine and succinyl moities of the SucCoA ligand.

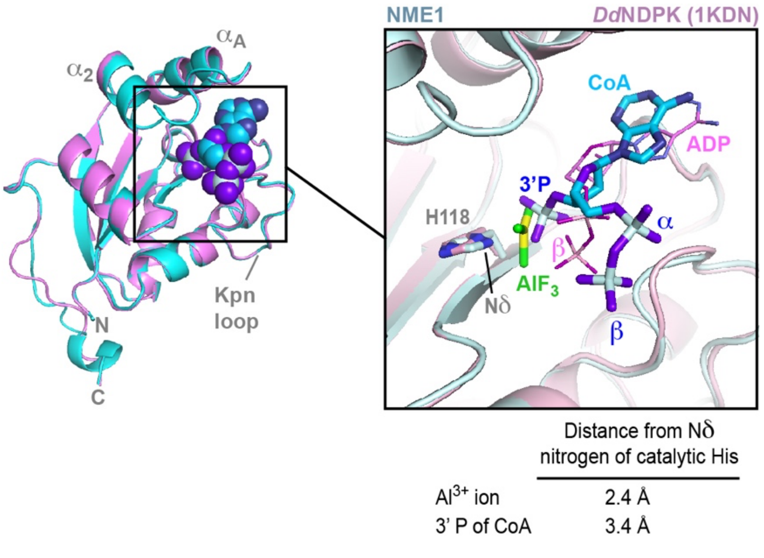

Figure S6. The CoA β-phosphate occupies the position of the ATP γ-phosphate prior to nucleophilic attack.

Alignment of the CoA-bound NME1 structure with that of *Dd*NDPK bound to ADP-AlF_3, solved at 2.8 Å-resolution (*86*). AlF_3 mimics the transition state of the ATP γ phosphate. The ADP ligand bound to *Dd*NDPK is shown with a thin stick radius. The CoA 3' phosphate is within 1.5 Å of the aluminum ion in AlF_3. Compared to the Al^{3+} ion, the CoA 3' phosphate is farther from the histidine's nucleophilic Nδ nitrogen and closer to the β-phosphate of ADP-AlF_3, suggesting that the CoA 3' phosphate occupies the position of the ATP γ-phosphate prior to nucleophilic attack.

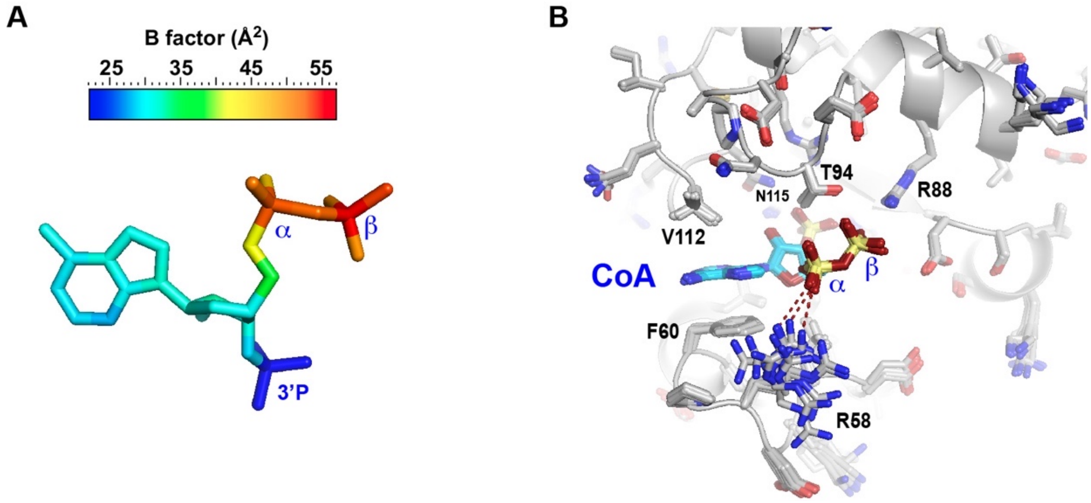

Figure S7. The α- and β-phosphates of the CoA nucleotide are poorly recognized by NME1.

(**A**) Structure of the NME1-bound CoA nucleotide with atoms colored according to their average crystallographic B factor, showing that the α- and β-phosphates are the most mobile atoms in the ligand. In contrast, the 3' phosphate is highly constrained by numerous H bond interactions with the protein, explaining the low values of its B factors. The average B factors shown were calculated by averaging over the 12 independent copies of the ligand in the crystal structure of SucCoA-bound NME1.

(**B**) Alignment of the 12 crystallographically independent subunits of NME1 in the crystal structure of SucCoA-bound NME1. Active site residues adopt a uniform sidechain conformation in all 12 subunits except for residue Arg58, which is located close to the CoA α-phosphate and exhibits considerable variability. Arg58 is within H bonding distance of the CoA α-phosphate in only 4 of the 12 monomers. H bonds are indicated as red dashed lines.

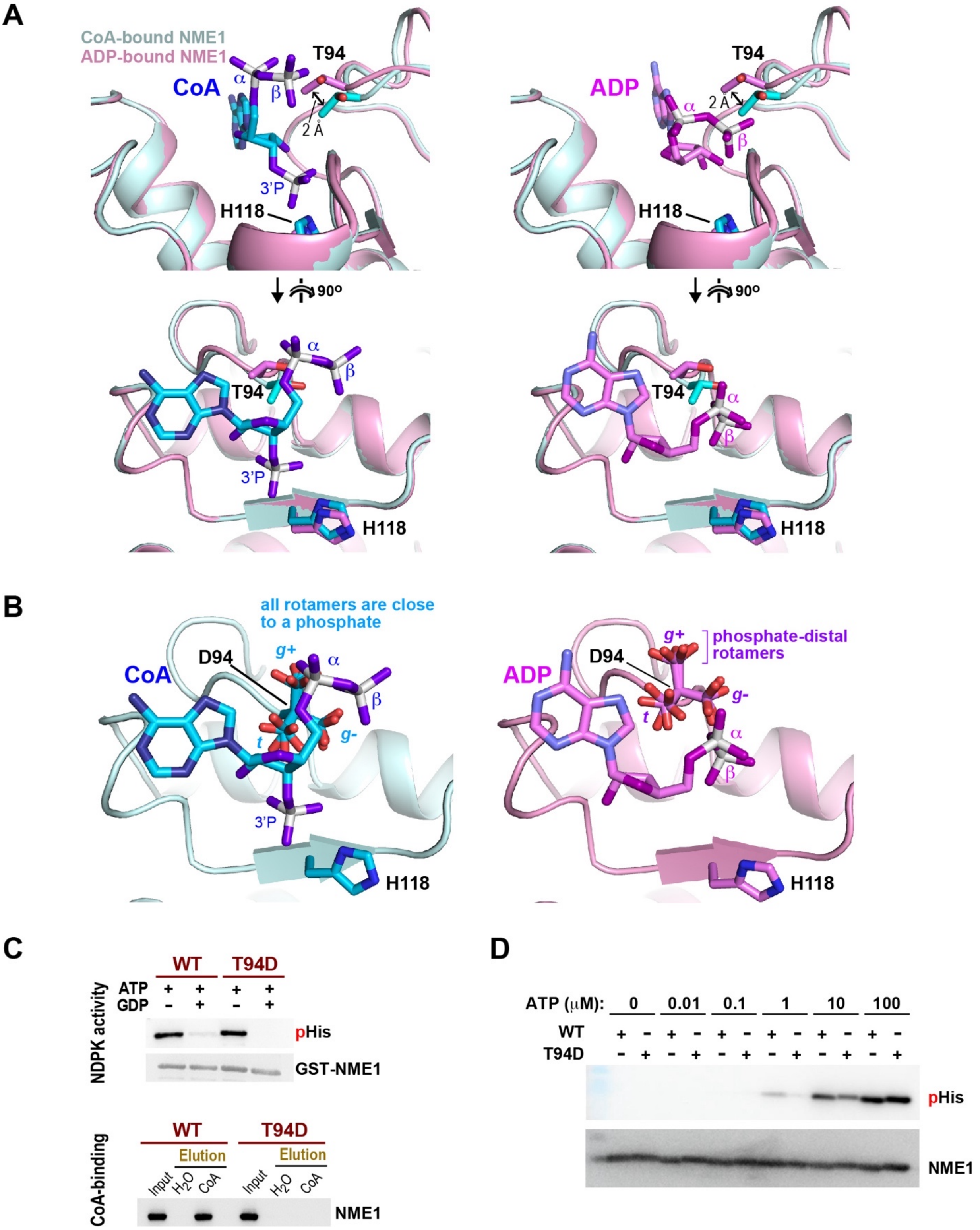

Figure S8 . Positional shift of Thr94 and analysis of the T94D mutant.

(**A**) Structural alignment of the CoA- and ADP-bound NME1 illustrating the shift of position of residue Thr94. Only one ligand, CoA or ADP, is shown in the left and right views, respectively. Thr94 is the only active site residue whose backbone atoms shift considerably between the CoA- and ADP-bound structures, resulting in a ~2 Å displacement of the sidechain hydroxyl and methyl groups. The lower view shows that these groups sit directly below the diphosphate of CoA but are more peripherally located with respect to that of ADP.

(**B**) *In silico* modeling of the T94D mutant. The Asp residue is modeled in its nine preferred sidechain conformations. The labels g^+, g^- and *t* refer to the *gauche*$^+$, *gauche*$^-$ and *trans* conformations of the χ_1 angle. *Left:* Replacing the Thr residue in the CoA-bound NME1 crystal structure by an Asp residue and modeling its sidechain conformations revealed that all Asp rotamers point their negatively charged carboxylate group towards one or more CoA phosphate groups located within unfavourably close proximity. *Right:* Repeating the same exercise with the ADP-bound structure revealed several Asp rotamers (all with χ_1 in a g^+ conformation) that point their carboxylate away from the ADP diphosphate, located over 5 Å away. This suggests that replacing Thr94 by an Asp residue should yield an NME1 mutant that could accommodate ADP in the active site by adopting a permissive Asp94 rotamer, whereas no Asp94 conformation could avoid an electrostatic repulsion with a bound CoA ligand.
(**C**) *Upper panel.* The NME1 T94D mutant retains notable NDPK activity. Both WT and mutant forms of GST-tagged NME1 are phosphorylated on histidine after incubation with ATP (100 μM) and become dephosphorylated upon incubation with GDP (200 μM). Proteins were detected using anti-phosphohistidine (pHis) and anti-NME1 antibodies (NME1).
Lower panel. The T94D mutant is defective for CoA-binding activity. WT and mutant NME1 were incubated with CoA beads, eluted with free CoA after a pull-down as described in Fig. 1B and the blot was probed with an anti-NME1 antibody.
(**D**) The T94D mutant has lower NDPK activity than WT. Both WT and mutant forms of NME1 are phosphorylated on histidine following incubation with ATP. The degree of phosphorylation observed at 1-10 μM ATP is approximately an order of magnitude lower for the mutant compared to the WT. Proteins were detected using anti-phosphohistidine (pHis) and anti-NME1 antibodies (NME1).

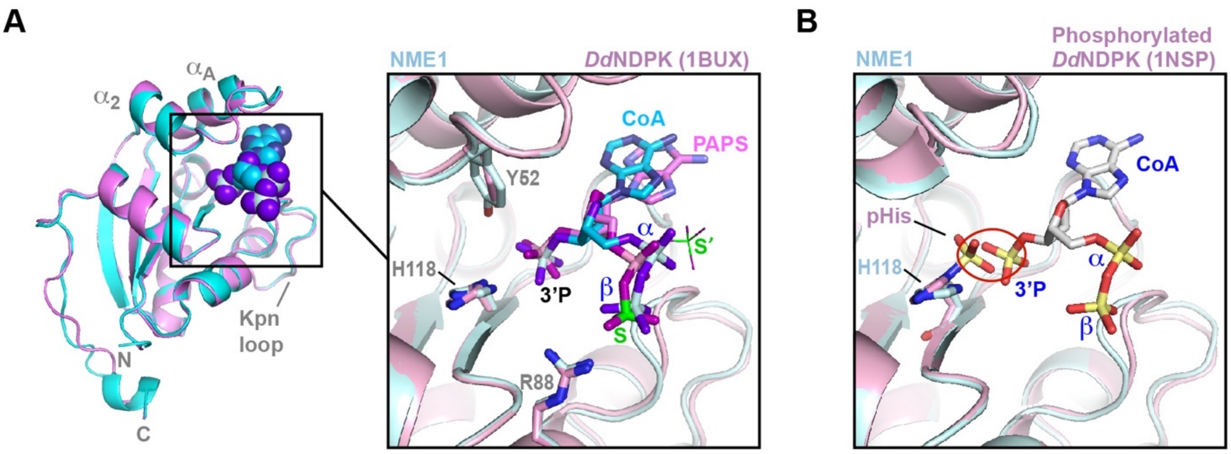

Figure S9. Structural analyses predict that CoA binding and histidine phosphorylation are mutually inhibitory.
(**A**) Alignment of SucCoA-bound NME1 and PAPS-bound *Dd*NDPK (PDB 1BUX). PAPS is chemically identical to the CoA nucleotide except that its sulfate group replaces the CoA β-phosphate. In the crystal structure, the PAPS sulfate group was observed to occupy two positions, each with partial occupancy, labeled S and S'. The latter is shown with a thinner stick radius. Residue numbering is that of mammalian NME1.
(**B**) Structural alignment of NME1:SucCoA with *Dd*NDPK (the drosophila NDPK) phosphorylated on His119 (equivalent to murine His118) (PDB 1NSP) predicts a steric clash and electrostatic repulsion between the histidine phosphate and the CoA 3' phosphate, located only 1.4 Å apart (red oval).

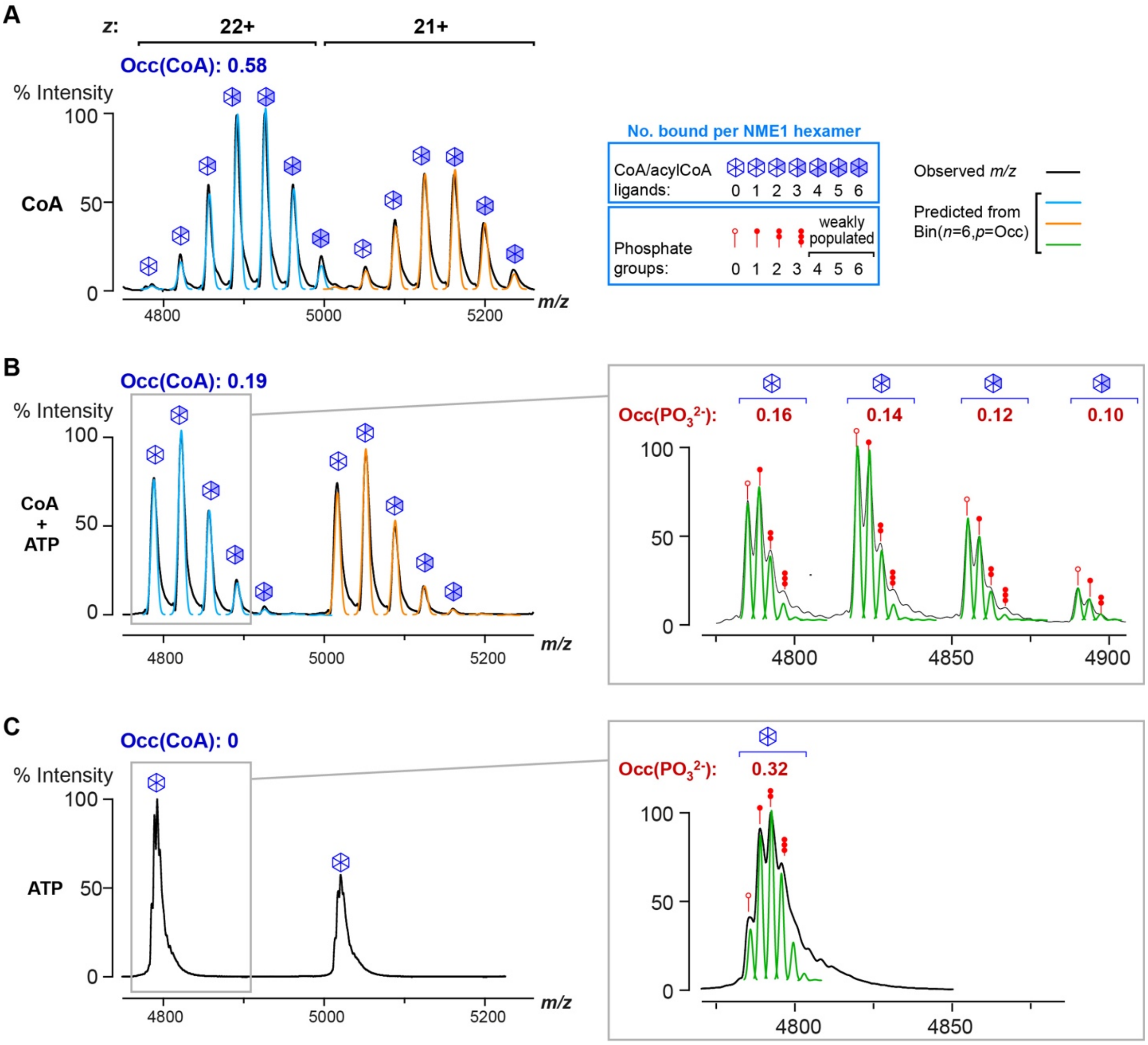

Figure S10. CoA binding and NME1 phosphorylation are mutually inhibitory.

Native MS spectra shown are the same as the corresponding spectra in Fig. 4A. Primary peaks in the spectra were modelled as a Gaussian function convoluted with a binomial distribution (blue and orange curves) to describe the distribution of CoA-bound NME1 states, as described in Fig. S3. *Insets*. A magnified view of individual peaks reveals sub-peaks corresponding to the presence of phosphoryl groups on NME1. The distribution of phosphorylation states was fitted as a Gaussian function convoluted with a binomial distribution (green curves) in a manner analogous to that used for primary peak fitting. The reasonable agreement between the recorded spectra and the fitted curves confirms that phosphorylation occurs independently at the six catalytic sites of the NME1 hexamer, allowing one to estimate the fraction of NME1 monomers that are phosphorylated [$Occ(PO_3^{2-})$].

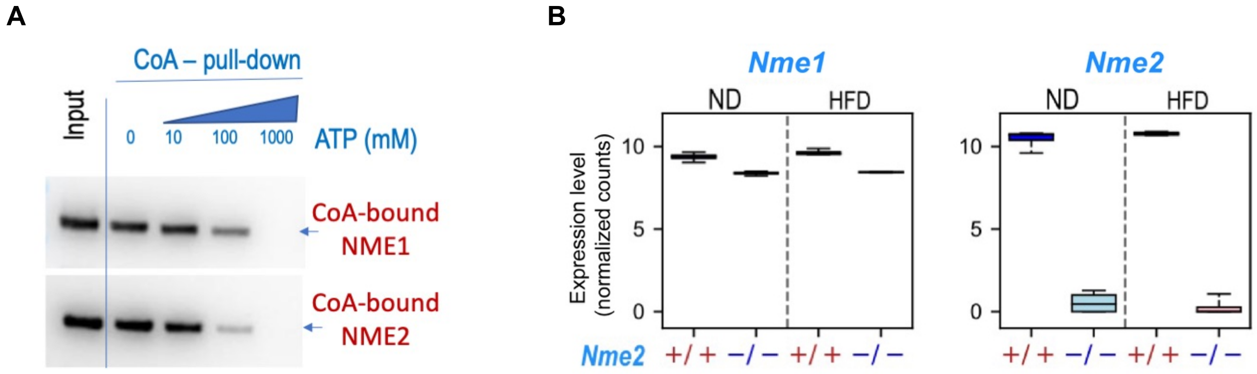

Figure S11. ATP-sensitive CoA binding by NME1 and NME2 and expression levels in *Nme2* ko mouse liver. (**A**) Competitive CoA and ATP binding by NME1 and NME2. Extracts from bacteria expressing GST-NME1 and GST-NME2 were used in CoA pull-down experiments in the presence of increasing amounts of ATP as indicated. CoA-bound NME1/2 were then visualised after immunoblotting using an anti-NME1/2 antibody. (**B**) *Nme1* and *Nme2* expression levels in wild-type and *Nme2* ko liver under either ND or HFD. The level of *Nme1* and *Nme2* expression from the transcriptomic data presented in Fig. 6 were analyzed and shown as boxplots. The values correspond to RNA-seq read counts normalized by DESeq method and then log-transformed with $\log_2$(DESeq+1).

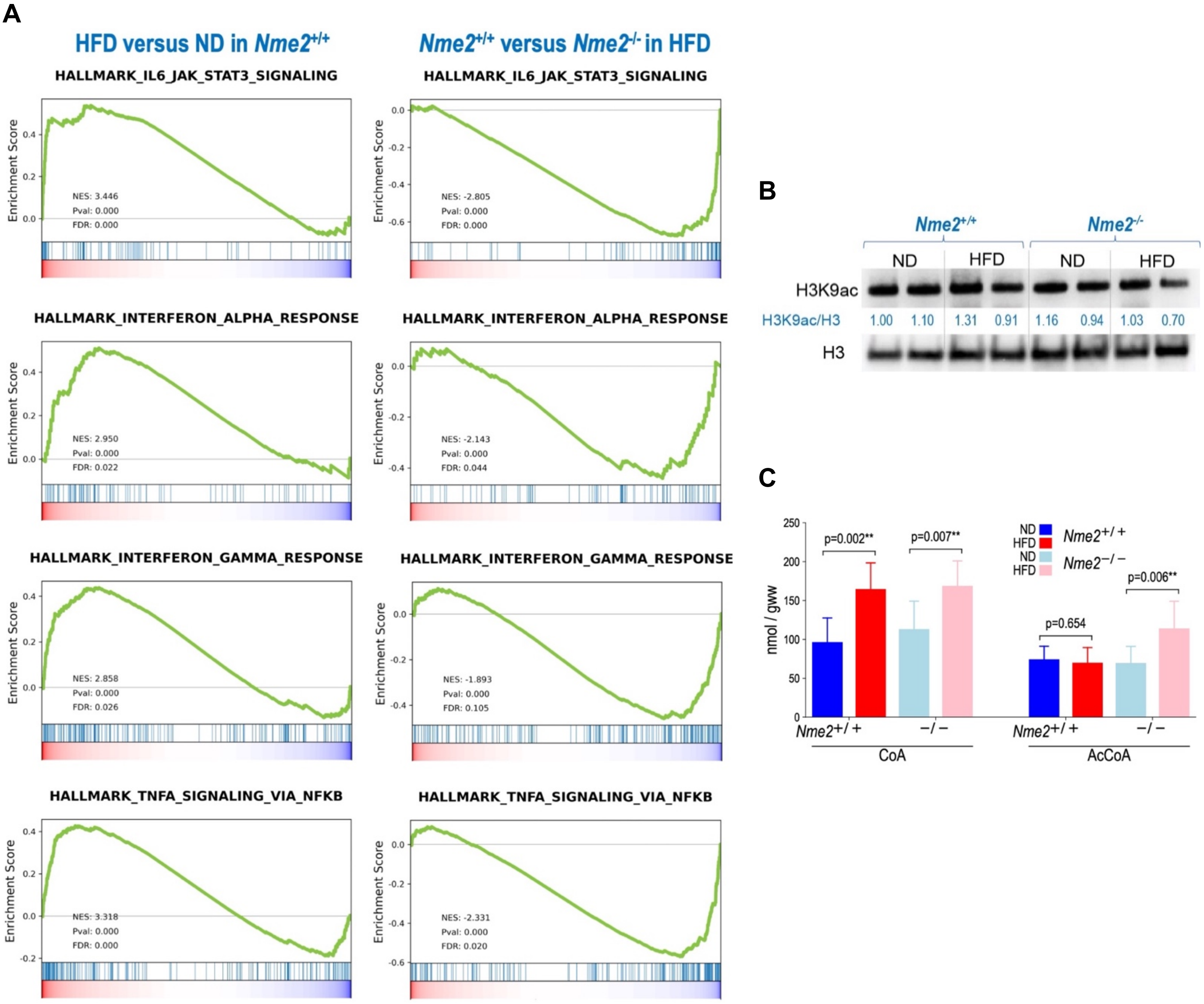

Figure S12. NME2-dependent activation of genes involved in protective signaling pathways in the liver of HFD mice.

(**A**) GSEA plots showing that gene sets corresponding to genes involved in the indicated signaling pathways are enriched in the hepatocytes of WT mice treated with HFD for 6 weeks compared to ND (left panels). These same gene sets are depleted in the liver of *Nme2*$^{-/-}$ mice under HFD compared to *Nme2*$^{+/+}$ mice, demonstrating that, in the absence of NME2, the corresponding signaling pathways are not activated under HFD.

(**B**) The levels of H3K9 acetylation and histone H3 in the liver extracts from mice treated as above were visualized by immunoblots using the corresponding antibodies. Two samples per condition were collected from independent mice. The normalized ratio of H3K9ac/H3 signals intensity for each sample is shown.

(**C**). The cellular concentrations of CoA and AcCoA were respectively measured in liver extracts from *Nme2*$^{+/+}$ and *Nme2*$^{-/-}$ mice. For each measurement, livers from independent mice were used as follows: *Nme2*$^{+/+}$ ND, $N = 9$; *Nme2*$^{+/+}$ HFD, $N = 4$; *Nme2*$^{-/-}$ ND, $N = 9$; *Nme2*$^{-/-}$ HFD, $N = 5$. The graphs show the average of the concentration values (in nmol/g of liver) and ± standard deviation. Student's *t*-test was used to calculate the *p*-values for HFD vs ND in all conditions. Statistical significance is indicated by the symbols *, ** and *** for *p*-values < 0.05, < 0.01 and < 0.001, respectively.

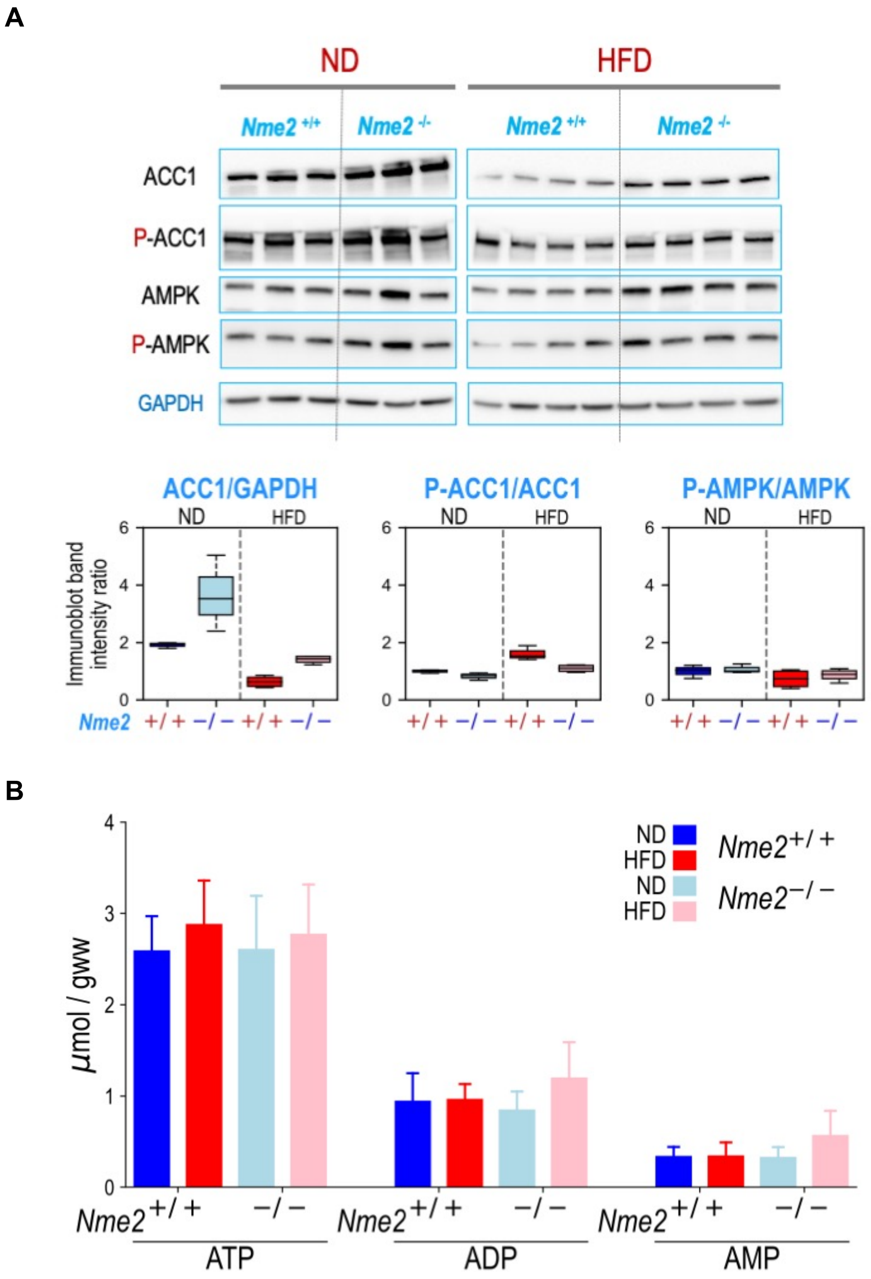

Figure S13. AMPK signaling is not involved in the response to a HFD challenge and the NME2-dependent control of fatty-acid synthesis.

(**A**) Extracts from livers from three independent *Nme2*$^{+/+}$ or *Nme2*$^{-/-}$ mice under ND or from four independent *Nme2*$^{+/+}$ or *Nme2*$^{-/-}$ mice under HFD for 6 weeks were probed with the indicated antibodies. Band intensities are shown as boxplots.

(**B**) Cellular concentrations of ATP, ADP and AMP measured in liver extracts from *Nme2*$^{+/+}$ and *Nme2*$^{-/-}$ mice. For each measurement, livers from independent mice were used as follows: *Nme2*$^{+/+}$ ND, *N* = 9; *Nme2*$^{+/+}$ HFD, *N* = 4; *Nme2*$^{-/-}$ ND, *N* = 9; *Nme2*$^{-/-}$ HFD, *N* = 5. The graph shows the average values (in µmol/g of liver) and SD.

Table S1. MS-based characterization of CoA-binding proteins. See accompanying auxiliary file.

Table S2. Crystallographic data collection and refinement statistics.

**Data collection**[1]	**CoA/NME1**	**SucCoA/NME1**	**ADP/NME1**
**PDB accession code**	7ZTK	7ZL8	7ZLW
**ESRF beamline**	ID30A-1	ID30A-1	ID30A-1
**Space group**	$P2_1$	$P2_1$	$P2_1$
NME1 **monomers/asym. unit**	6	12	6
**Unit cell parameters:**			
a **(Å)**	54.17	53.84	51.66
b **(Å)**	69.45	69.09	70.19
c **(Å)**	116.30	225.13	113.17
β	90.06°	89.72°	90.96°
**Resolution range (Å)**	49.13 - 2.60	56.28 – 1.96	59.65 – 2.20
**(outer shell)**	(2.693 - 2.60)	(2.03 – 1.96)	(2.23 – 2.20)
**Total measured reflections**	84,560 (8703)	388,841 (39,041)	99,867 (8875)
**Unique reflections**	26,589 (2649)	115,209 (11,894)	40,517 (3536)
**Multiplicity**	3.2 (3.3)	3.4 (3.3)	2.5 (2.5)
**Completeness (%)**	99.23 (98.53)	95.43 (96.61)	98.2 (98.9)
**Mean (I)/sd (I)**	3.37 (0.84)	5.04 (0.57)	11.4 (1.7)
R_{merge}	0.25 (1.05)	0.18 (1.92)	0.03 (0.31)
R_{meas}	0.31 (1.25)	0.22 (2.30)	0.04 (0.44)
$CC_{1/2}$	0.94 (0.54)	0.995 (0.41)	0.999 (0.88)
**Refinement**[1]			
**Resolution range (Å)**	49.13 - 2.60	56.28 – 1.96	59.65 – 2.20
**(outer shell)**	(2.693 – 2.60)	(2.03 – 1.96)	(2.279 -2.20)
**Reflections used for refinement**	26593 (2613)	113575 (11498)	39106 (3035)
**Reflections used for** R_{free}	1322 (102)	5728 (630)	1992 (141)
R_{work}	27.73 (39.01)	21.82 (37.13)	17.13 (22.81)
R_{free}	28.93 (37.76)	25.10 (38.94)	22.01 (28.75)
**Number of non-hydrogen**	7603	16043	7745
**atoms**	7272	14718	7158
**Protein**	254	372	135
**Ligands**	77	953	452
**Solvent**			
**Protein residues**	912	1831	895
**RMS deviations**			
**Bond distances** (Å)	0.003	0.007	0.004
Bond angles (**°**)	0.61	0.91	0.79
**Ramachandran analysis (%)**			
**Favored/allowed/outliers**	97.22 /2.11/0.67	97.18/2.19/0.66	97.50/1.82/0.68
**Molprobity analysis**			
**Clash score/Overall score**	9.02/2.06	4.93/1.47	4.13/1.52
**Average B-factor**	47.68	26.93	38.10
**Protein**	46.94	26.61	38.08
**Ligands**	70.81	38.12	46.12
**Solvent**	41.05	27.62	36.12
**Number of TLS groups**	6	12	6

[1] Values in parenthesis are for the highest-resolution shell.

Table S3. RMS differences between ligand-bound NME1/2 structures.

Aligned pair of structures				No. monomers/ asymmetric unit			No. pairwise alignments		RMSD[3] (150 Cα atoms aligned)			
Species	Protein	Ligand	PDB ID	Total	Ligand-bound	Un-bound[1]	All	Bound[2]	Min	Max	Mean[4] (All)	Mean[4] (Bound)
human	NME1	ADP	2HVD	3	3	0	18	15	0.573	1.309	0.758	0.649
mouse	"	"	7ZLW	6	5	1					± 0.254	± 0.039
mouse	NME1	ADP	7ZLW	6	5	1	36	30	0.292	1.189	0.480	0.342
"	"	CoA	7ZTK	6	6	0					± 0.314	± 0.028
mouse	NME1	ADP	7ZLW	6	5	1	72	60	0.313	1.185	0.500	0.365
"	"	SucCoA	7ZL8	12	12	0					± 0.304	± 0.025
mouse	NME1	CoA	7ZTK	6	6	0	72	72	0.269	0.353	0.305	0.305
"	"	SucCoA	7ZL8	12	12	0					± 0.018	± 0.018
mouse	NME1	ADP	7ZLW	6	5	1	36	15	0.312	2.328	1.028	0.357
human	NME2	myrCoA	7KPF	6	3	3					± 0.814	± 0.023
mouse	NME1	CoA	7ZTK	6	6	0	36	18	0.300	2.295	0.958	0.345
human	NME2	myrCoA	7KPF	6	3	3					± 0.853	± 0.024
mouse	NME1	SucCoA	7ZL8	12	12	0	72	36	0.296	2.357	0.971	0.350
human	NME2	myrCoA	7KPF	6	3	3					± 0.864	± 0.026

[1] Certain monomers in some of the crystal structures have an empty ligand-binding site. In most of these cases the residues defining the binding site differ considerably in conformation between the ligand-bound and unbound monomers.
[2] Number of structural alignments excluding those involving a monomer with an empty binding site.
[3] Root-mean-square deviation of aligned structures.
[4] Values represent the mean ±S.D. calculated over the corresponding number of pairwise alignments.

Table S4. Intersections between genes presented as GSEA (fig. S12a) and genes that could be regulated by H3K9 acetylation/deacetylation around their TSS (Fig. 7C)

Hallmark	Total No. genes	No. common with Fig. 7C	Common genes
IL6_JAK_STAT3_SIGNALING	80	4	*Il1r1, Tlr2, Jun, Socs1*
INTERFERON_ALPHA_RESPONSE	94	0	--
INTERFERON_GAMMA_RESPONSE	188	6	*H2-Aa, Mthfd2, Socs1, Icam1, Cdkn1a, Tnfaip3*
TNFA_SIGNALING_VIA_NFKB	196	8	*Btg2, Egr1, Tlr2, Efna1, Icam1, Cdkn1a, Tnfaip3, Jun*

Table S5. Key resources information

REAGENT or RESOURCE	SOURCE	IDENTIFIER
**Antibodies**		
Rabbit monoclonal anti-NME2 (it also recognizes NME1)	Abcam	ab131329
Rabbit monoclonal anti-N1-Phosphohistidine (1-pHis), clone SC1-1	Sigma-Aldrich	MABS1330
Rabbit polyclonal anti-Acetyl-CoA Carboxylase1 (ACC1)	Cell Signaling Technology	#3662
Rabbit polyclonal anti-PhosphoACC1 (Ser79)	Cell Signaling Technology	#3661
Rabbit polyclonal anti-ATP-Citrate Lyase (Acly)	Cell Signaling Technology	#4332
Rabbit monoclonal anti-Acetyl-CoA synthetase (ACSS2)(AceCs1)	Cell Signaling Technology	#3658
Rabbit polyclonal anti-AMPKα	Cell Signaling Technology	#2532
Rabbit monoclonal anti-PhosphoAMPKα (Thr172)	Cell Signaling Technology	#2535
Rabbit monoclonal anti-β-Tubulin	Cell Signaling Technology	#2128
Mouse monoclonal anti-α-Tubulin	Sigma-Aldrich	#T5168
Mouse monoclonal anti-β-actin	Sigma-Aldrich	#A5441
Goat polyclonal anti-GAPDH	Santa Cruz	#sc-20357
Mouse anti-Histone H3K9ac	Active motif	#61251
Mouse Anti-H4K5ac	Active motif	#61523
Rabbit monoclonal anti-H3K14ac	PTMbiolabs	# PTM-113RM
Rabbit polyclonal Anti-H3	Abcam	#Ab1791
Rabbit polyclonal Anti-H4	Active motif	#39269
Rabbit polyclonal anti-H3	Abcam	ab1791
Mouse monoclonal anti-GST	Santa Cruz	sc-138
Mouse Anti-FLAG	Sigma-Aldrich	# F3165
**Bacterial and virus strains**		
One Shot TOP10	Invitrogen	C404010
E. coli BL21-Gold(D3)	Agilent	#230132
**Chemicals, peptides, and recombinant proteins**		
Dulbecco's modified Eagle medium (DMEM) plus glutaMax	Gibco	#31966021
FBS	Corning	#35-079-CV
EDTA-free protease inhibitor	Roche	#04693159001
DMEM/F12 medium	Gibco	#31331028
Glutathione Sepharose	Cytiva	#17-5248
PEG 3350	Sigma-Aldrich	#1546547
PEG 1000	Sigma-Aldrich	#1546489
Tridecanoic acid	Sigma-Aldrich	#T0502
Phosphatidylcholine	Avanti	#850370
Lipofectamine RNAiMAX	Invitrogen	#13778075
Coenzyme A-Agarose	Sigma-Aldrich	C7013
Coenzyme A	Sigma-Aldrich	C4282
Acetyl-CoA	Sigma-Aldrich	A2056
Succinyl-CoA	Sigma-Aldrich	S1129
ATP	Sigma-Aldrich	11140965001

ADP	Sigma-Aldrich	C7344
GDP	Sigma-Aldrich	G7127
3'-Dephosphocoenzyme A	Sigma-Aldrich	D3385
NAD	Sigma-Aldrich	N1511
FAD	Sigma-Aldrich	F6625
ADP-ribose	Sigma-Aldrich	C7344
n-Butyric acid	Sigma-Aldrich	B-2503
Dynabeads protein G	Thermo-fisher	10004D
Nuclease S7	Sigma-Aldrich	#10107921001
Mouse WT/T94D NME1	This paper	N/A
Mouse WT/T94D GST-NME1	This paper	N/A
Recombinant human FLAG-P300	Active motif	#81158
Recombinant human FLAG-GCN5	Active motif	#31591
Recombinant human mononucleosome	Active motif	#81070
**Critical commercial assays**		
NucleoSpin RNA kit	Machery-Nagel	740955.50
QuickChange Multi-Site Directed Mutagenesis kit	Agilent	#200514
Triglycerides detection kit	Erba Mannheim	BLT00059
Illumina® Stranded Total RNA Prep, Ligation with Ribo-Zero Plus	Illumina	20040525
Qubit® dsDNA HS Assay Kit	Life Technologies	Q32854
HS NGS Fragment Kit (1-6000bp)	Agilent	DNF-474-0500
MicroPlex Library Preparation Kit v3	Diagenode	C05010001
NextSeq® 500/550 High Output v2.5 (150 cycles)	Illumina	20024907
**Deposited data**		
RNA-seq data	This paper	GEO: GSE198688
Chip-seq data	This paper	GEO: GSE198688
NME1-ADP structure	This paper	PDB:7ZLW
NME1-CoA	This paper	PDB: 7ZTK
NME1-SuccinylCoA	This paper	PDB:7ZL8
Mass spectrometry proteomics data of CoA binding factors (testis samples)	This paper	ProteomeXchange Consortium PMID: 30395289
**Experimental models: Organisms/strains**		
Mouse: C57BL/6N-Nme2tm1.1(KOMP)Vlcg/MbpMmucd	MMRRC (Mutant Mouse Resource & Research Centers supported by NIH)	stock #: 048819-UCD
**Oligonucleotides**		
siRNA pGL2 luciferase (si-control)	Eurogentec	SR-CL011-005
siRNA targeting sequence: human Nme1 CAAGUUGGCAGGAACAUUA	This paper	N/A
siRNA targeting sequence: human Acly UUCUUGAUCAGCUUUCUCGUGAGGG	This paper	N/A
Primers for cloning cds mouse Nme1<br>FW: ATGGCCAACAGTGAGCGCACCT<br>RW: TCACTCATAGATCCAGTTCTGCG	This paper	N/A
Primer to introduce mutation T94D in cds of Nme1 plasmids.<br>GTGATGCTTGGAGAGgaCAACCCCGCAGACTCTAAGC	This paper	N/A

Recombinant DNA		
pETM-11-His-Nme1	This paper	N/A
pETM-11-Nme1mutant (T94D)	This paper	N/A
pETM-11 (N-ter His_6)	EMBL	N/A
pGEX-4T-1-Nme1	This paper	N/A
pGEX-4T-1-Nme1mutant (T94D)	This paper	N/A
pGEX-4T-1 (N-ter GST)	Addgene	27458001
**Software and algorithms**		
Masslynx 4.0 software	Waters Corporation, Manchester, U.K.)	https://www.waters.com
Massign software package	Massign	https://doi.org/10.1021/ac300056a
GraphPad Prism	GraphPad	Graphpad.com
MicroCal PEAQ-ITC Analysis Software	MalvernPanalytical	https://www.malvernpanalytical.com/
R Software	R Core Team	https://www.R-project.org/
**Other**		
Normal Diet food (standard)	Safe, France	A03
High Fat Diet food	Safe, France	U8978 Version 19
Superdex 200 16/60 column	Cytiva	28-9893-35
Amicon Ultra-15 10K	Millipore	#UFC901096

Supplementary Auxiliary Files

Table S1. MS-based characterization of CoA-binding proteins.
Excel file summarizing MS-based characterization of CoA-binding proteins.

Data S1. Original images of Western blots and gels.
PDF file containing original images of Western blots and gels shown in Figures 1C, 1D, 2B, 4B, 4C, 5A, 5B, 7D, 7E, S2B, S8C, S8D, S12B and S13A.

Data S2. Original data used to generate graphs, boxplots and histograms.
Excel file containing raw data used to generate graphs, boxplots and histograms shown in Figures 5C, 5D, 5F, 6C, S11B, S12C, S13A and S13B.

REFERENCES AND NOTES

1. M. Wallace, C. M. Metallo, Tracing insights into de novo lipogenesis in liver and adipose tissues. *Semin. Cell Dev. Biol.* **108**, 65–71 (2020).

2. F. Ameer, L. Scandiuzzi, S. Hasnain, H. Kalbacher, N. Zaidi, De novo lipogenesis in health and disease. *Metabolism* **63**, 895–902 (2014).

3. J. E. Lambert, M. A. Ramos-Roman, J. D. Browning, E. J. Parks, Increased de novo lipogenesis is a distinct characteristic of individuals with nonalcoholic fatty liver disease. *Gastroenterology* **146**, 726–735 (2014).

4. G. I. Smith, M. Shankaran, M. Yoshino, G. G. Schweitzer, M. Chondronikola, J. W. Beals, A. L. Okunade, B. W. Patterson, E. Nyangau, T. Field, C. B. Sirlin, S. Talukdar, M. K. Hellerstein, S. Klein, Insulin resistance drives hepatic de novo lipogenesis in nonalcoholic fatty liver disease. *J. Clin. Invest.* **130**, 1453–1460 (2020).

5. K. H. M. Roumans, L. Lindeboom, P. Veeraiah, C. M. E. Remie, E. Phielix, B. Havekes, Y. M. H. Bruls, M. Brouwers, M. Stahlman, M. Alssema, H. P. F. Peters, R. de Mutsert, B. Staels, M. R. Taskinen, J. Boren, P. Schrauwen, V. B. Schrauwen-Hinderling, Hepatic saturated fatty acid fraction is associated with de novo lipogenesis and hepatic insulin resistance. *Nat. Commun.* **11**, 1891 (2020).

6. P. Kasper, A. Martin, S. Lang, F. Kutting, T. Goeser, M. Demir, H. M. Steffen, NAFLD and cardiovascular diseases: A clinical review. *Clin. Res. Cardiol.* **110**, 921–937 (2021).

7. Y. Lee, H. T. M. Lai, M. C. de Oliveira Otto, R. N. Lemaitre, B. McKnight, I. B. King, X. Song, G. S. Huggins, A. R. Vest, D. S. Siscovick, D. Mozaffarian, Serial biomarkers of de novo lipogenesis fatty acids and incident heart failure in older adults: The cardiovascular health study. *J. Am. Heart Assoc.* **9**, e014119 (2020).

8. N. Koundouros, G. Poulogiannis, Reprogramming of fatty acid metabolism in cancer. *Br. J. Cancer* **122**, 4–22 (2020).

9. Z. T. Schug, J. Vande Voorde, E. Gottlieb, The metabolic fate of acetate in cancer. *Nat. Rev. Cancer* **16**, 708–717 (2016).

10. N. Zaidi, J. V. Swinnen, K. Smans, ATP-citrate lyase: A key player in cancer metabolism. *Cancer Res.* **72**, 3709–3714 (2012).

11. R. Ke, Q. Xu, C. Li, L. Luo, D. Huang, Mechanisms of AMPK in the maintenance of ATP balance during energy metabolism. *Cell Biol. Int.* **42**, 384–392 (2018).

12. M. Boissan, M. L. Lacombe, Learning about the functions of NME/NM23: Lessons from knockout mice to silencing strategies. *Naunyn Schmiedebergs Arch. Pharmacol.* **384**, 421–431 (2011).

13. K. Adam, J. Ning, J. Reina, T. Hunter, NME/NM23/NDPK and histidine phosphorylation. *Int. J. Mol. Sci.* **21**, 5848–5873 (2020).

14. B. Y. K. Yu, M. A. Tossounian, S. D. Hristov, R. Lawrence, P. Arora, Y. Tsuchiya, S. Y. Peak-Chew, V. Filonenko, S. Oxenford, R. Angell, J. Gouge, M. Skehel, I. Gout, Regulation of metastasis suppressor NME1 by a key metabolic cofactor coenzyme A. *Redox Biol.* **44**, 101978–101999 (2021).

15. S. Zhang, O. D. Nelson, I. R. Price, C. Zhu, X. Lu, I. R. Fernandez, R. S. Weiss, H. Lin, Long-chain fatty acyl coenzyme A inhibits NME1/2 and regulates cancer metastasis. *Proc. Natl. Acad. Sci. U.S.A.* **119**, e2117013119 (2022).

16. L. Lv, Q. Lei, Proteins moonlighting in tumor metabolism and epigenetics. *Front. Med.* **15**, 383–403 (2021).

17. A. Goudarzi, H. Shiota, S. Rousseaux, S. Khochbin, Genome-scale acetylation-dependent histone eviction during spermatogenesis. *J. Mol. Biol.* **426**, 3342–3349 (2014).

18. A. Goudarzi, D. Zhang, H. Huang, S. Barral, O. K. Kwon, S. Qi, Z. Tang, T. Buchou, A. L. Vitte, T. He, Z. Cheng, E. Montellier, J. Gaucher, S. Curtet, A. Debernardi, G. Charbonnier, D. Puthier, C. Petosa, D. Panne, S. Rousseaux, R. G. Roeder, Y. Zhao, S. Khochbin, Dynamic competing histone H4 K5K8 acetylation and butyrylation are hallmarks of highly active gene promoters. *Mol. Cell* **62**, 169–180 (2016).

19. H. Shiota, S. Barral, T. Buchou, M. Tan, Y. Coute, G. Charbonnier, N. Reynoird, F. Boussouar, M. Gerard, M. Zhu, L. Bargier, D. Puthier, F. Chuffart, E. Bourova-Flin, S. Picaud, P. Filippakopoulos, A. Goudarzi, Z. Ibrahim, D. Panne, S. Rousseaux, Y. Zhao, S. Khochbin, Nut directs p300-dependent, genome-wide h4 hyperacetylation in male germ cells. *Cell Rep.* **24**, 3477–3487.e6 (2018).

20. W. Pusch, M. Balvers, G. F. Weinbauer, R. Ivell, The rat endozepine-like peptide gene is highly expressed in late haploid stages of male germ cell development. *Biol. Reprod.* **63**, 763–768 (2000).

21. E. Boeri Erba, L. Signor, C. Petosa, Exploring the structure and dynamics of macromolecular complexes by native mass spectrometry. *J. Proteomics* **222**, 103799–103815 (2020).

22. L. Cervoni, I. Lascu, Y. Xu, P. Gonin, M. Morr, M. Merouani, J. Janin, A. Giartosio, Binding of nucleotides to nucleoside diphosphate kinase: A calorimetric study. *Biochemistry* **40**, 4583–4589 (2001).

23. Y. Chen, S. Morera, C. Pasti, A. Angusti, N. Solaroli, M. Veron, J. Janin, S. Manfredini, D. Deville-Bonne, Adenosine phosphonoacetic acid is slowly metabolized by NDP kinase. *Med. Chem.* **1**, 529–536 (2005).

24. C. Dumas, I. Lascu, S. Morera, P. Glaser, R. Fourme, V. Wallet, M. L. Lacombe, M. Veron, J. Janin, X-ray structure of nucleoside diphosphate kinase. *EMBO J.* **11**, 3203–3208 (1992).

25. B. Schneider, Y. W. Xu, J. Janin, M. Veron, D. Deville-Bonne, 3'-Phosphorylated nucleotides are tight binding inhibitors of nucleoside diphosphate kinase activity. *J. Biol. Chem.* **273**, 28773–28778 (1998).

26. M. F. Giraud, F. Georgescauld, I. Lascu, A. Dautant, Crystal structures of S120G mutant and wild type of human nucleoside diphosphate kinase A in complex with ADP. *J. Bioenerg. Biomembr.* **38**, 261–264 (2006).

27. J. Janin, C. Dumas, S. Morera, Y. Xu, P. Meyer, M. Chiadmi, J. Cherfils, Three-dimensional structure of nucleoside diphosphate kinase. *J. Bioenerg. Biomembr.* **32**, 215–225 (2000).

28. J. Bourdais, R. Biondi, S. Sarfati, C. Guerreiro, I. Lascu, J. Janin, M. Veron, Cellular phosphorylation of anti-HIV nucleosides. Role of nucleoside diphosphate kinase. *J. Biol. Chem.* **271**, 7887–7890 (1996).

29. B. Schneider, Y. W. Xu, O. Sellam, R. Sarfati, J. Janin, M. Veron, D. Deville-Bonne, Pre-steady state of reaction of nucleoside diphosphate kinase with anti-HIV nucleotides. *J. Biol. Chem.* **273**, 11491–11497 (1998).

30. S. Morera, M. Chiadmi, G. LeBras, I. Lascu, J. Janin, Mechanism of phosphate transfer by nucleoside diphosphate kinase: X-ray structures of the phosphohistidine intermediate of the enzymes from Drosophila and Dictyostelium. *Biochemistry* **34**, 11062–11070 (1995).

31. E. H. Postel, X. Zou, D. A. Notterman, K. M. La Perle, Double knockout Nme1/Nme2 mouse model suggests a critical role for NDP kinases in erythroid development. *Mol. Cell. Biochem.* **329**, 45–50 (2009).

32. A. Carrer, J. L. D. Parris, S. Trefely, R. A. Henry, D. C. Montgomery, A. Torres, J. M. Viola, Y. M. Kuo, I. A. Blair, J. L. Meier, A. J. Andrews, N. W. Snyder, K. E. Wellen, Impact of a high-fat diet on tissue acyl-CoA and histone acetylation levels. *J. Biol. Chem.* **292**, 3312–3322 (2017).

33. J. A. Duarte, F. Carvalho, M. Pearson, J. D. Horton, J. D. Browning, J. G. Jones, S. C. Burgess, A high-fat diet suppresses de novo lipogenesis and desaturation but not elongation and triglyceride synthesis in mice. *J. Lipid Res.* **55**, 2541–2553 (2014).

34. A. M. Lundsgaard, J. B. Holm, K. A. Sjoberg, K. N. Bojsen-Moller, L. S. Myrmel, E. Fjaere, B. A. H. Jensen, T. S. Nicolaisen, J. R. Hingst, S. L. Hansen, S. Doll, P. E. Geyer, A. S. Deshmukh, J. J. Holst, L. Madsen, K. Kristiansen, J. F. P. Wojtaszewski, E. A. Richter, B. Kiens, Mechanisms preserving insulin action during high dietary fat intake. *Cell Metab.* **29**, 50–63.e4 (2019).

35. J. C. Newman, A. J. Covarrubias, M. Zhao, X. Yu, P. Gut, C. P. Ng, Y. Huang, S. Haldar, E. Verdin, Ketogenic diet reduces midlife mortality and improves memory in aging mice. *Cell Metab.* **26**, 547–557.e8 (2017).

36. M. Ozaki, Cellular and molecular mechanisms of liver regeneration: Proliferation, growth, death and protection of hepatocytes. *Semin. Cell Dev. Biol.* **100**, 62–73 (2020).

37. W. C. Hsieh, B. M. Sutter, H. Ruess, S. D. Barnes, V. S. Malladi, B. P. Tu, Glucose starvation induces a switch in the histone acetylome for activation of gluconeogenic and fat metabolism genes. *Mol. Cell* **82**, 60–74.e5 (2022).

38. M. Mendoza, G. Egervari, S. Sidoli, G. Donahue, D. C. Alexander, P. Sen, B. A. Garcia, S. L. Berger, Enzymatic transfer of acetate on histones from lysine reservoir sites to lysine activating sites. *Sci. Adv.* **8**, eabj5688 (2022).

39. L. A. Gates, J. Shi, A. D. Rohira, Q. Feng, B. Zhu, M. T. Bedford, C. A. Sagum, S. Y. Jung, J. Qin, M. J. Tsai, S. Y. Tsai, W. Li, C. E. Foulds, B. W. O'Malley, Acetylation on histone H3 lysine 9 mediates a switch from transcription initiation to elongation. *J. Biol. Chem.* **292**, 14456–14472 (2017).

40. B. J. E. Martin, J. Brind'Amour, A. Kuzmin, K. N. Jensen, Z. C. Liu, M. Lorincz, L. J. Howe, Transcription shapes genome-wide histone acetylation patterns. *Nat. Commun.* **12**, 210–218 (2021).

41. R. H. Wright, A. Lioutas, F. Le Dily, D. Soronellas, A. Pohl, J. Bonet, A. S. Nacht, S. Samino, J. Font-Mateu, G. P. Vicent, M. Wierer, M. A. Trabado, C. Schelhorn, C. Carolis, M. J. Macias, O. Yanes, B. Oliva, M. Beato, ADP-ribose-derived nuclear ATP synthesis by NUDIX5 is required for chromatin remodeling. *Science* **352**, 1221–1225 (2016).

42. B. D. G. Page, N. C. K. Valerie, R. H. G. Wright, O. Wallner, R. Isaksson, M. Carter, S. G. Rudd, O. Loseva, A. S. Jemth, I. Almlof, J. Font-Mateu, S. Llona-Minguez, P. Baranczewski, F. Jeppsson, E. Homan, H. Almqvist, H. Axelsson, S. Regmi, A. L. Gustavsson, T. Lundback, M. Scobie, K. Stromberg, P. Stenmark, M. Beato, T. Helleday, Targeted NUDT5 inhibitors block hormone signaling in breast cancer cells. *Nat. Commun.* **9**, 250–263 (2018).

43. J. D. Chow, R. T. Lawrence, M. E. Healy, J. E. Dominy, J. A. Liao, D. S. Breen, F. L. Byrne, B. M. Kenwood, C. Lackner, S. Okutsu, V. R. Mas, S. H. Caldwell, J. L. Tomsig, G. J. Cooney, P. B. Puigserver, N. Turner, D. E. James, J. Villen, K. L. Hoehn, Genetic inhibition of hepatic acetyl-CoA carboxylase activity increases liver fat and alters global protein acetylation. *Mol. Metab.* **3**, 419–431 (2014).

44. L. Galdieri, A. Vancura, Acetyl-CoA carboxylase regulates global histone acetylation. *J. Biol. Chem.* **287**, 23865–23876 (2012).

45. M. Rios Garcia, B. Steinbauer, K. Srivastava, M. Singhal, F. Mattijssen, A. Maida, S. Christian, H. Hess-Stumpp, H. G. Augustin, K. Muller-Decker, P. P. Nawroth, S. Herzig, M. Berriel Diaz, Acetyl-CoA carboxylase 1-dependent protein acetylation controls breast cancer metastasis and recurrence. *Cell Metab.* **26**, 842–855.e5 (2017).

46. B. Ponugoti, D. H. Kim, Z. Xiao, Z. Smith, J. Miao, M. Zang, S. Y. Wu, C. M. Chiang, T. D. Veenstra, J. K. Kemper, SIRT1 deacetylates and inhibits SREBP-1C activity in regulation of hepatic lipid metabolism. *J. Biol. Chem.* **285**, 33959–33970 (2010).

47. M. Amemiya-Kudo, H. Shimano, T. Yoshikawa, N. Yahagi, A. H. Hasty, H. Okazaki, Y. Tamura, F. Shionoiri, Y. Iizuka, K. Ohashi, J. Osuga, K. Harada, T. Gotoda, R. Sato, S. Kimura, S. Ishibashi, N. Yamada, Promoter analysis of the mouse sterol regulatory element-binding protein-1c gene. *J. Biol. Chem.* **275**, 31078–31085 (2000).

48. S. N. Dankel, B. Bjorndal, C. Lindquist, M. L. Grinna, C. R. Rossmann, P. Bohov, O. Nygard, S. Hallstrom, E. Strand, R. K. Berge, Hepatic energy metabolism underlying differential lipidomic responses to high-carbohydrate and high-fat diets in male wistar rats. *J. Nutr.* **151**, 2610–2621 (2021).

49. X. Gao, S. H. Lin, F. Ren, J. T. Li, J. J. Chen, C. B. Yao, H. B. Yang, S. X. Jiang, G. Q. Yan, D. Wang, Y. Wang, Y. Liu, Z. Cai, Y. Y. Xu, J. Chen, W. Yu, P. Y. Yang, Q. Y. Lei, Acetate functions as an epigenetic metabolite to promote lipid synthesis under hypoxia. *Nat. Commun.* **7**, 11960–11973 (2016).

50. S. Zhao, C. Jang, J. Liu, K. Uehara, M. Gilbert, L. Izzo, X. Zeng, S. Trefely, S. Fernandez, A. Carrer, K. D. Miller, Z. T. Schug, N. W. Snyder, T. P. Gade, P. M. Titchenell, J. D. Rabinowitz, K. E. Wellen, Dietary fructose feeds hepatic lipogenesis via microbiota-derived acetate. *Nature* **579**, 586–591 (2020).

51. S. Horie, M. Isobe, T. Suga, Changes in CoA pools in hepatic peroxisomes of the rat under various conditions. *J. Biochem.* **99**, 1345–1352 (1986).

52. J. R. Williamson, B. E. Corkey, Assay of citric acid cycle intermediates and related compounds–Update with tissue metabolite levels and intracellular distribution. *Methods Enzymol.* **55**, 200–222 (1979).

53. R. Leonardi, Y. M. Zhang, C. O. Rock, S. Jackowski, Coenzyme A: Back in action. *Prog. Lipid Res.* **44**, 125–153 (2005).

54. J. A. Idell-Wenger, L. W. Grotyohann, J. R. Neely, Coenzyme A and carnitine distribution in normal and ischemic hearts. *J. Biol. Chem.* **253**, 4310–4318 (1978).

55. Y. Taniguchi, P. J. Choi, G. W. Li, H. Chen, M. Babu, J. Hearn, A. Emili, X. S. Xie, Quantifying *E. coli* proteome and transcriptome with single-molecule sensitivity in single cells. *Science* **329**, 533–538 (2010).

56. M. Beck, A. Schmidt, J. Malmstroem, M. Claassen, A. Ori, A. Szymborska, F. Herzog, O. Rinner, J. Ellenberg, R. Aebersold, The quantitative proteome of a human cell line. *Mol. Syst. Biol.* **7**, 549–556 (2011).

57. B. Schwanhausser, D. Busse, N. Li, G. Dittmar, J. Schuchhardt, J. Wolf, W. Chen, M. Selbach, Global quantification of mammalian gene expression control. *Nature* **473**, 337–342 (2011).

58. Y. Mitsui, E. L. Schneider, Relationship between cell replication and volume in senescent human diploid fibroblasts. *Mech. Ageing Dev.* **5**, 45–56 (1976).

59. N. T. Snider, S. V. Weerasinghe, A. Singla, J. M. Leonard, S. Hanada, P. C. Andrews, A. S. Lok, M. B. Omary, Energy determinants GAPDH and NDPK act as genetic modifiers for hepatocyte inclusion formation. *J. Cell Biol.* **195**, 217–229 (2011).

60. S. Barral, Y. Morozumi, H. Tanaka, E. Montellier, J. Govin, M. de Dieuleveult, G. Charbonnier, Y. Coute, D. Puthier, T. Buchou, F. Boussouar, T. Urahama, F. Fenaille, S. Curtet, P. Hery, N. Fernandez-Nunez, H. Shiota, M. Gerard, S. Rousseaux, H. Kurumizaka, S. Khochbin, Histone variant H2A.L.2 guides transition protein-dependent protamine assembly in male germ cells. *Mol. Cell* **66**, 89–101.e8 (2017).

61. T. Buchou, M. Tan, S. Barral, A. L. Vitte, S. Rousseaux, J. Arechaga, S. Khochbin, Purification and analysis of male germ cells from adult mouse testis. *Methods Mol. Biol.* **1510**, 159–168 (2017).

62. S. R. Fuhs, J. Meisenhelder, A. Aslanian, L. Ma, A. Zagorska, M. Stankova, A. Binnie, F. Al-Obeidi, J. Mauger, G. Lemke, J. R. Yates III, T. Hunter, Monoclonal 1- and 3-phosphohistidine antibodies: New tools to study histidine phosphorylation. *Cell* **162**, 198–210 (2015).

63. S. Gratia, L. Kay, L. Potenza, A. Seffouh, V. Novel-Chate, C. Schnebelen, P. Sestili, U. Schlattner, M. Tokarska-Schlattner, Inhibition of AMPK signalling by doxorubicin: At the crossroads of the cardiac responses to energetic, oxidative, and genotoxic stress. *Cardiovasc. Res.* **95**, 290–299 (2012).

64. Y. I. Shurubor, M. D'Aurelio, J. Clark-Matott, E. P. Isakova, Y. I. Deryabina, M. F. Beal, A. J. L. Cooper, B. F. Krasnikov, Determination of coenzyme A and acetyl-coenzyme A in biological samples using HPLC with UV detection. *Molecules* **22**, 1388–1400 (2017).

65. X. Liu, D. E. Cooper, A. A. Cluntun, M. O. Warmoes, S. Zhao, M. A. Reid, J. Liu, P. J. Lund, M. Lopes, B. A. Garcia, K. E. Wellen, D. G. Kirsch, J. W. Locasale, Acetate production from glucose and coupling to mitochondrial metabolism in mammals. *Cell* **175**, 502–513.e13 (2018).

66. M. G. Casabona, Y. Vandenbrouck, I. Attree, Y. Coute, Proteomic characterization of *Pseudomonas aeruginosa* PAO1 inner membrane. *Proteomics* **13**, 2419–2423 (2013).

67. D. Bouyssie, A. M. Hesse, E. Mouton-Barbosa, M. Rompais, C. Macron, C. Carapito, A. Gonzalez de Peredo, Y. Coute, V. Dupierris, A. Burel, J. P. Menetrey, A. Kalaitzakis, J. Poisat, A. Romdhani, O. Burlet-Schiltz, S. Cianferani, J. Garin, C. Bruley, Proline: An efficient and user-friendly software suite for large-scale proteomics. *Bioinformatics* **36**, 3148–3155 (2020).

68. Y. Coute, C. Bruley, T. Burger, Beyond target-decoy competition: Stable validation of peptide and protein identifications in mass spectrometry-based discovery proteomics. *Anal. Chem.* **92**, 14898–14906 (2020).

69. S. Wieczorek, F. Combes, C. Lazar, Q. Giai Gianetto, L. Gatto, A. Dorffer, A. M. Hesse, Y. Coute, M. Ferro, C. Bruley, T. Burger, DAPAR & ProStaR: Software to perform statistical analyses in quantitative discovery proteomics. *Bioinformatics* **33**, 135–136 (2017).

70. W. Kabsch, XDS. *Acta Crystallogr. D Biol. Crystallogr.* **66**, 125–132 (2010).

71. C. Vonrhein, C. Flensburg, P. Keller, A. Sharff, O. Smart, W. Paciorek, T. Womack, G. Bricogne, Data processing and analysis with the autoPROC toolbox. *Acta Crystallogr. D Biol. Crystallogr.* **67**, 293–302 (2011).

72. P. R. Evans, G. N. Murshudov, How good are my data and what is the resolution? *Acta Crystallogr. D Biol. Crystallogr.* **69**, 1204–1214 (2013).

73. A. J. McCoy, R. W. Grosse-Kunstleve, P. D. Adams, M. D. Winn, L. C. Storoni, R. J. Read, Phaser crystallographic software. *J. Appl. Cryst.* **40**, 658–674 (2007).

74. D. E. Mortenson, G. J. Brighty, L. Plate, G. Bare, W. Chen, S. Li, H. Wang, B. F. Cravatt, S. Forli, E. T. Powers, K. B. Sharpless, I. A. Wilson, J. W. Kelly, "Inverse drug discovery" strategy to identify proteins that are targeted by latent electrophiles as exemplified by aryl fluorosulfates. *J. Am. Chem. Soc.* **140**, 200–210 (2018).

75. P. D. Adams, P. V. Afonine, G. Bunkoczi, V. B. Chen, I. W. Davis, N. Echols, J. J. Headd, L. W. Hung, G. J. Kapral, R. W. Grosse-Kunstleve, A. J. McCoy, N. W. Moriarty, R. Oeffner, R. J. Read, D. C. Richardson, J. S. Richardson, T. C. Terwilliger, P. H. Zwart, PHENIX: A comprehensive Python-based system for macromolecular structure solution. *Acta Crystallogr. D Biol. Crystallogr.* **66**, 213–221 (2010).

76. P. Emsley, B. Lohkamp, W. G. Scott, K. Cowtan, Features and development of Coot. *Acta Crystallogr. D Biol. Crystallogr.* **66**, 486–501 (2010).

77. A. Dobin, C. A. Davis, F. Schlesinger, J. Drenkow, C. Zaleski, S. Jha, P. Batut, M. Chaisson, T. R. Gingeras, STAR: Ultrafast universal RNA-seq aligner. *Bioinformatics* **29**, 15–21 (2013).

78. S. Anders, P. T. Pyl, W. Huber, HTSeq—A Python framework to work with high-throughput sequencing data. *Bioinformatics* **31**, 166–169 (2015).

79. R Core Team. R: A language and environment for statistical computing. R Foundation for Statistical Computing, Vienna, Austria. https://R-project.org/ (2021).

80. S. Anders, W. Huber, Differential expression analysis for sequence count data. *Genome Biol.* **11**, R106 (2010).

81. M. I. Love, W. Huber, S. Anders, Moderated estimation of fold change and dispersion for RNA-seq data with DESeq2. *Genome Biol.* **15**, 550 (2014).

82. H. Varet, L. Brillet-Gueguen, J. Y. Coppee, M. A. Dillies, SARTools: A DESeq2- and EdgeR-based R pipeline for comprehensive differential analysis of RNA-Seq data. *PLOS ONE* **11**, e0157022 (2016).

83. B. Langmead, S. L. Salzberg, Fast gapped-read alignment with Bowtie 2. *Nat. Methods* **9**, 357–359 (2012).

84. Y. Liao, G. K. Smyth, W. Shi, featureCounts: An efficient general purpose program for assigning sequence reads to genomic features. *Bioinformatics* **30**, 923–930 (2014).

85. F. Ramirez, F. Dundar, S. Diehl, B. A. Gruning, T. Manke, deepTools: A flexible platform for exploring deep-sequencing data. *Nucleic Acids Res.* **42**, W187–W191 (2014).

86. Y. W. Xu, S. Morera, J. Janin, J. Cherfils, AlF3 mimics the transition state of protein phosphorylation in the crystal structure of nucleoside diphosphate kinase and MgADP. *Proc. Natl. Acad. Sci. U.S.A.* **94**, 3579–3583 (1997).
