# Supplementary figures and images for: Nucleoside diphosphate kinases 1 and 2 regulate a protective liver response to a high-fat diet
Source: Sci Adv. 2023 Sep 6;9(36):eadh0140. doi: 10.1126/sciadv.adh0140 (PMC10482350; doi:10.1126/sciadv.adh0140)

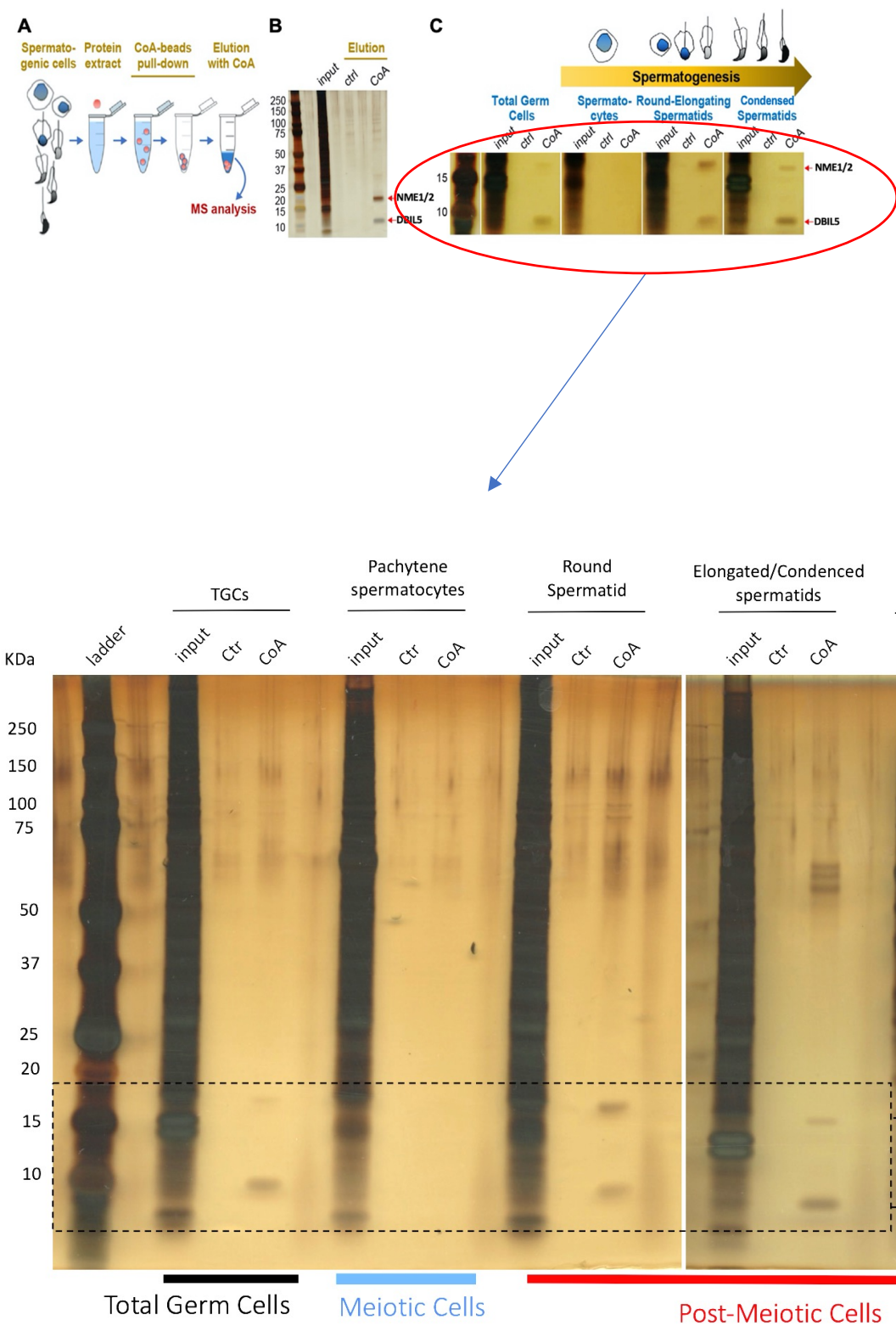

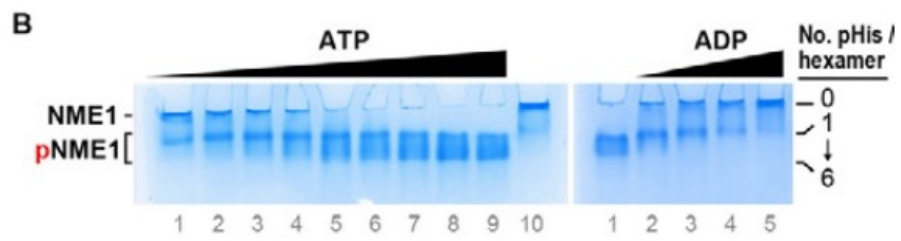

The protein-containing part of the gel is shown

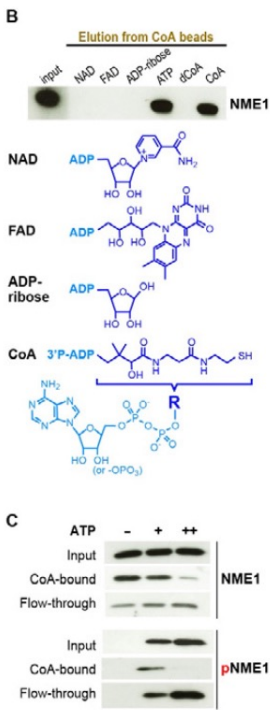

4B

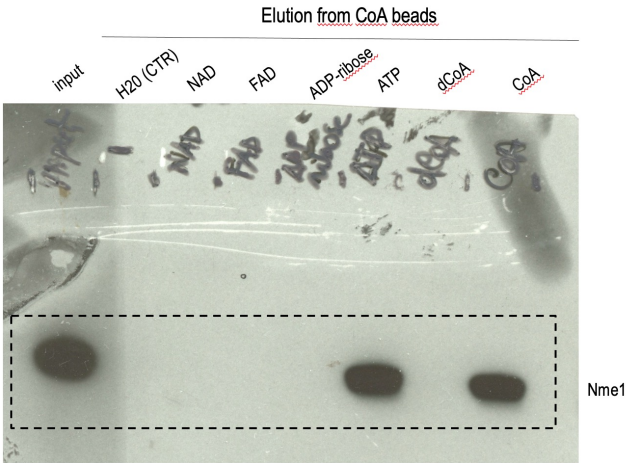

4C

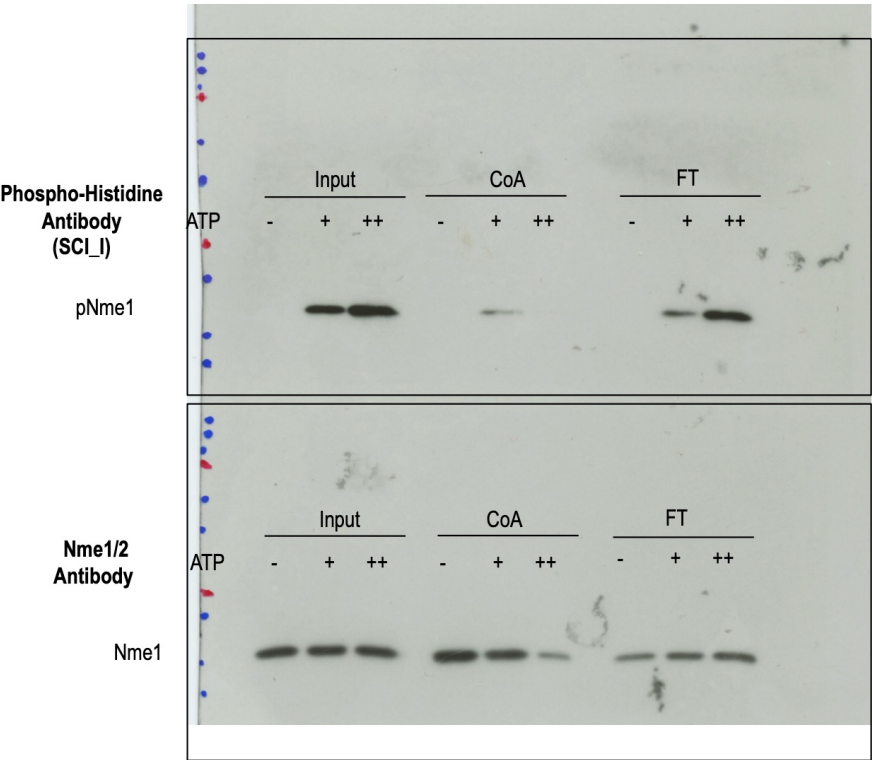

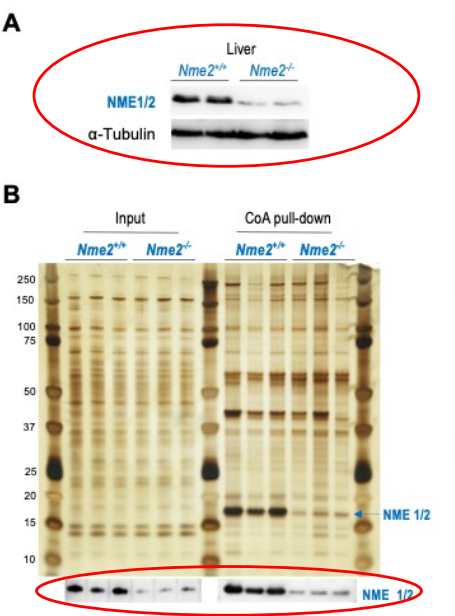

Fig.5A

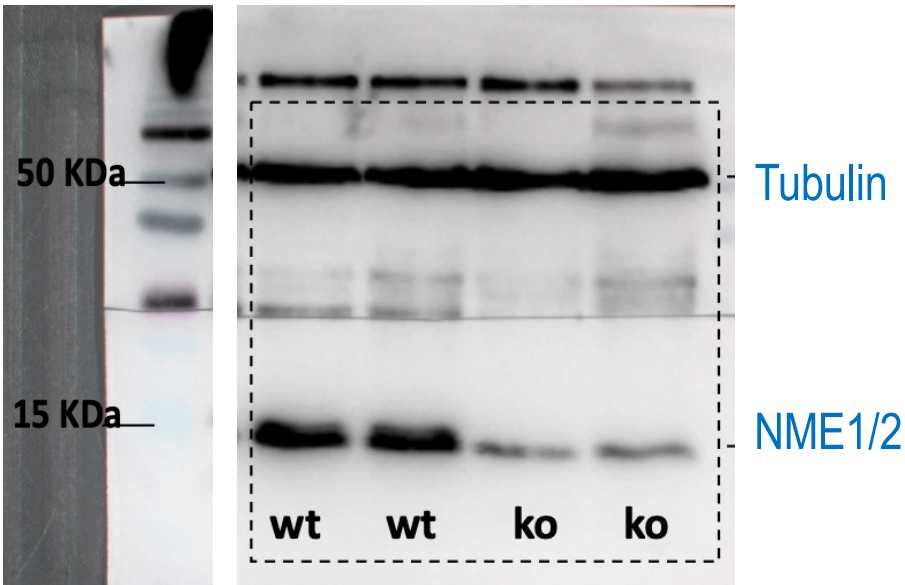

Fig.5B

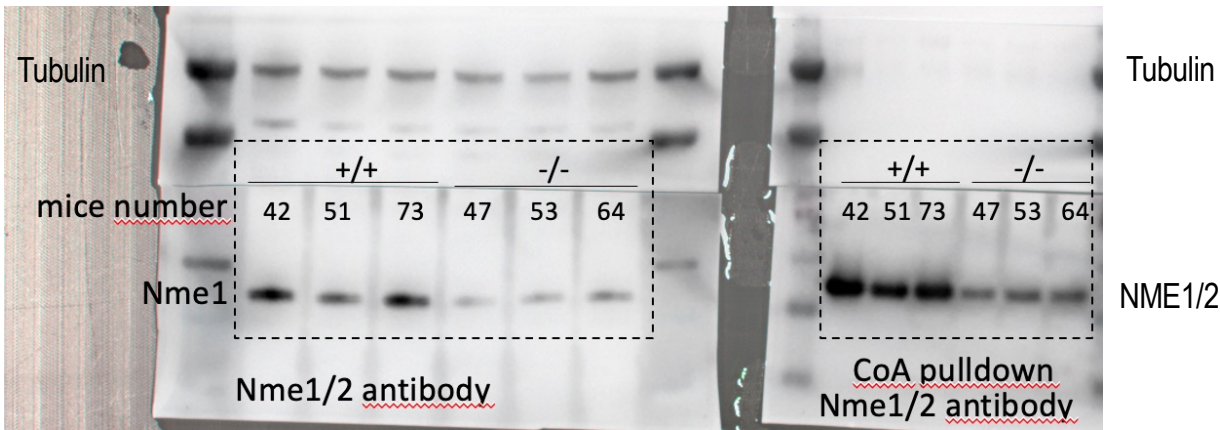

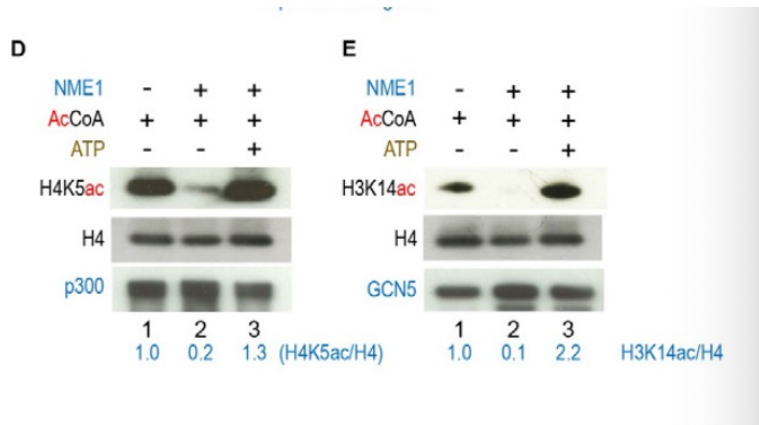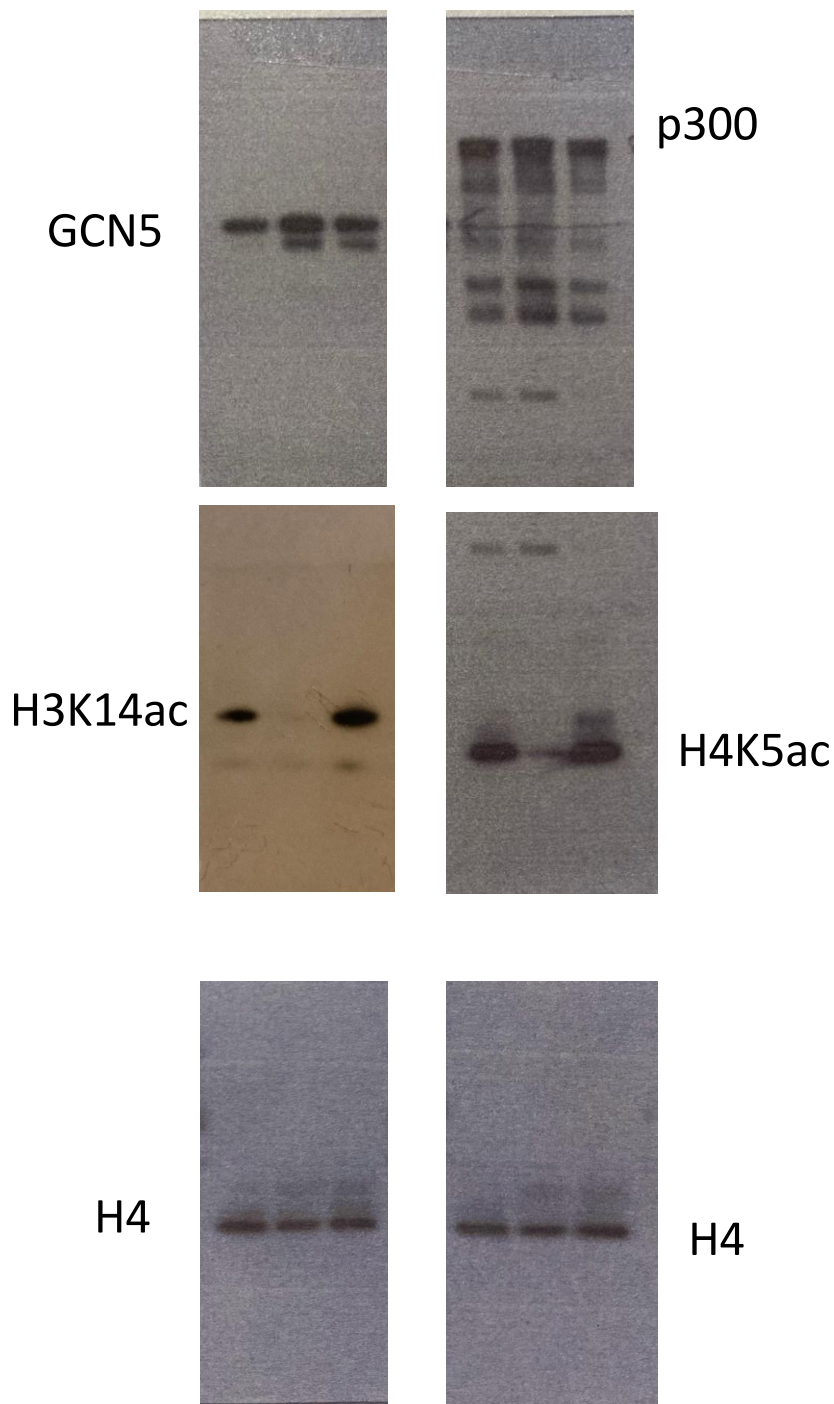

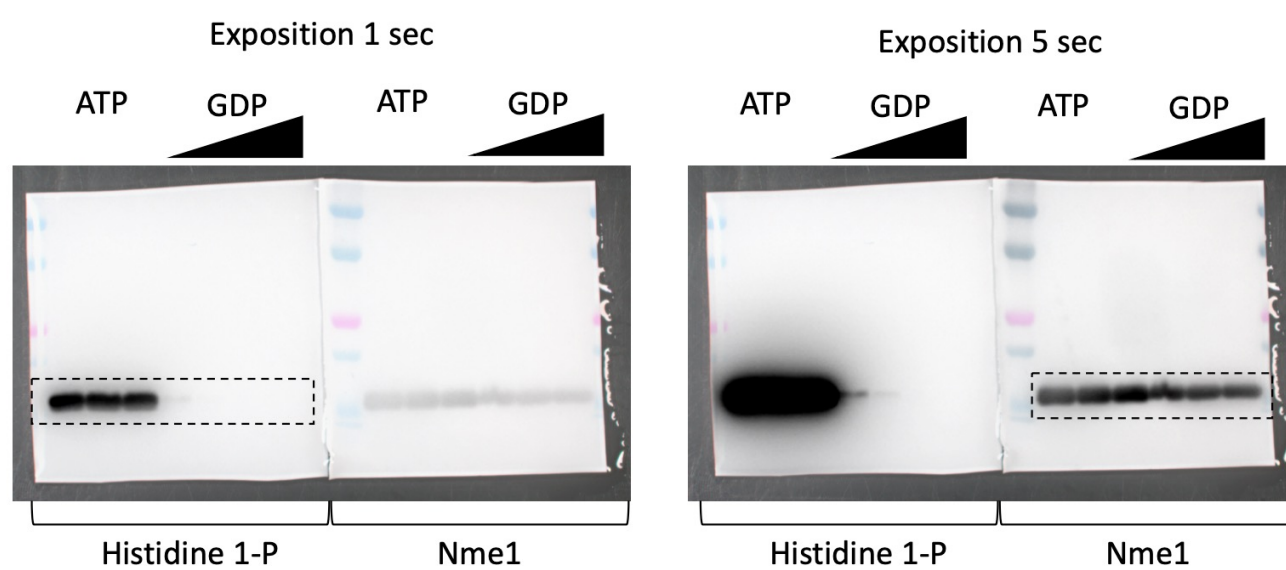

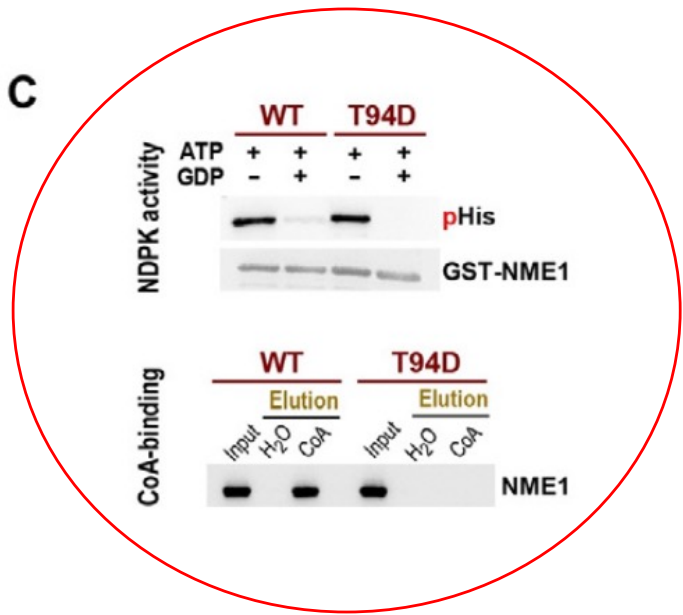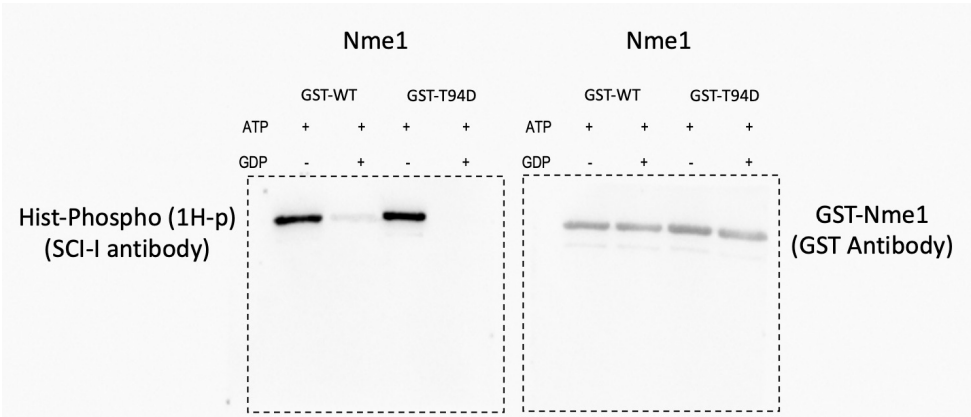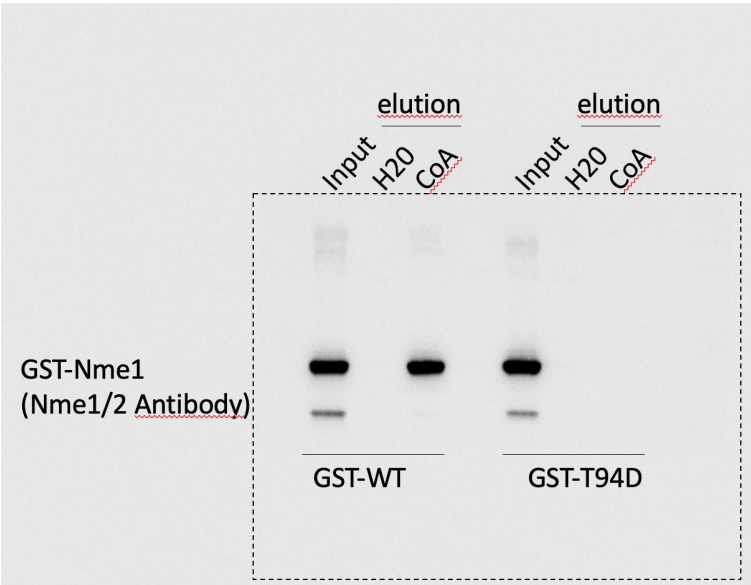

D

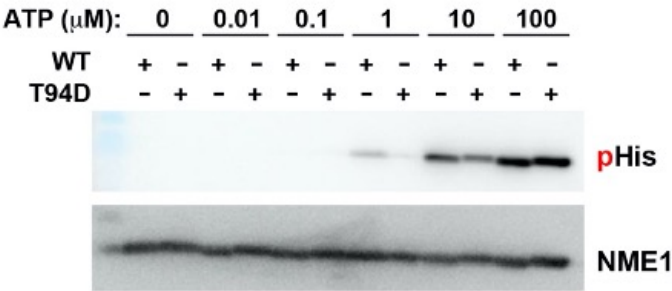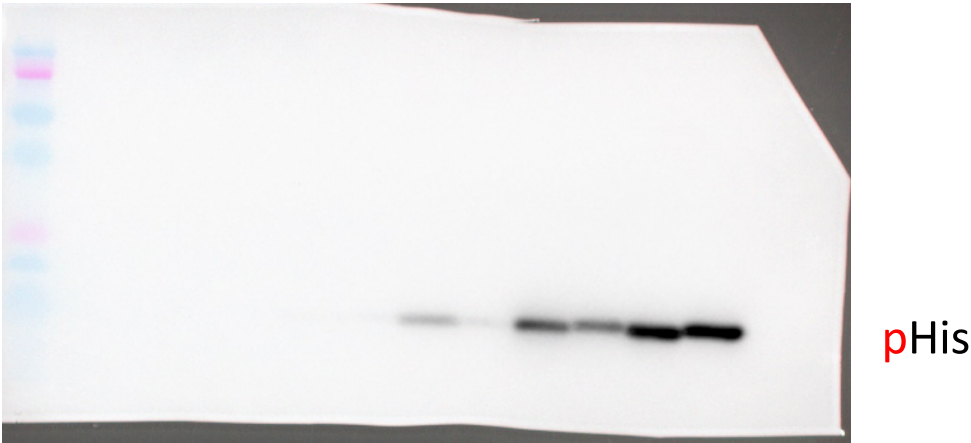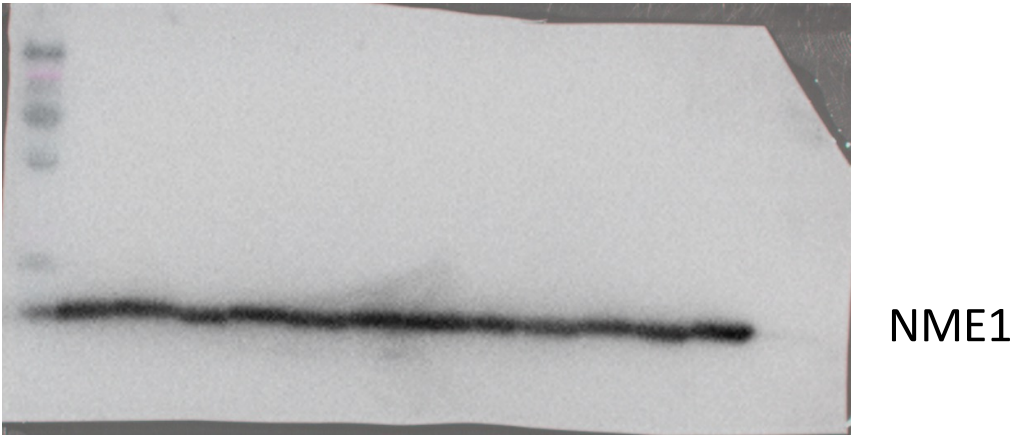

**B**

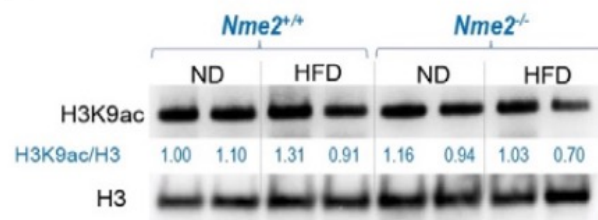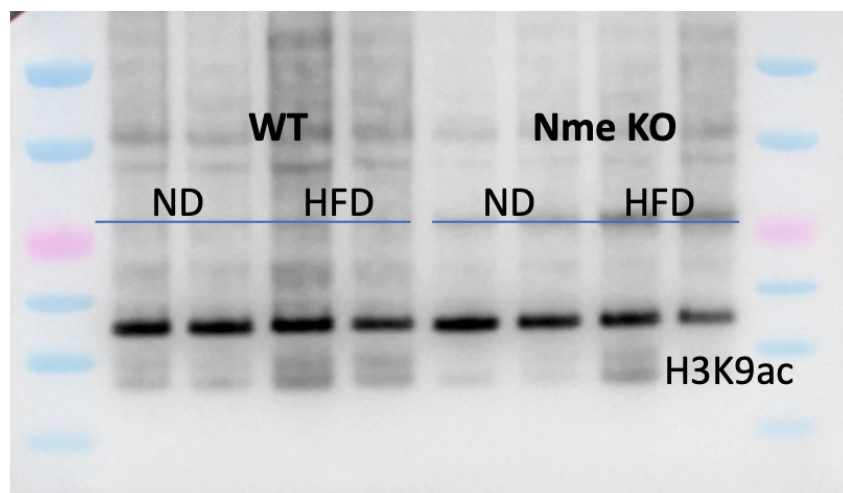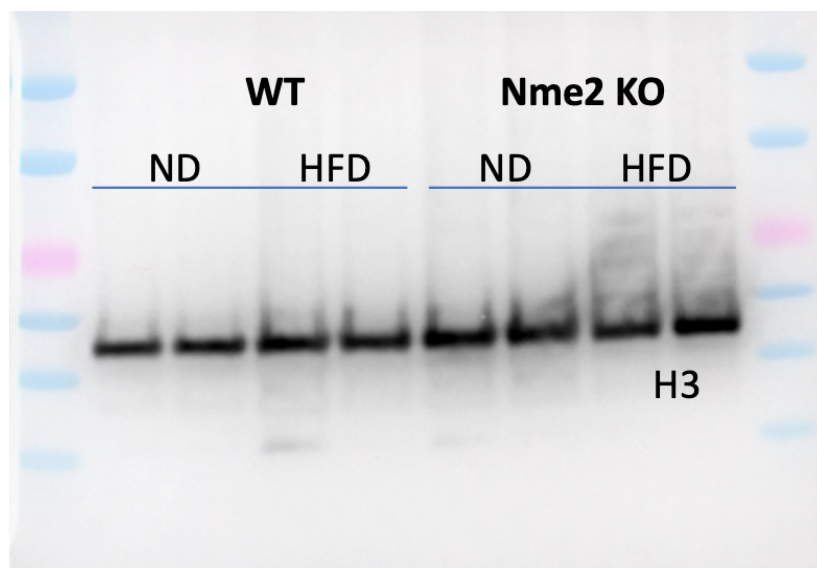

A

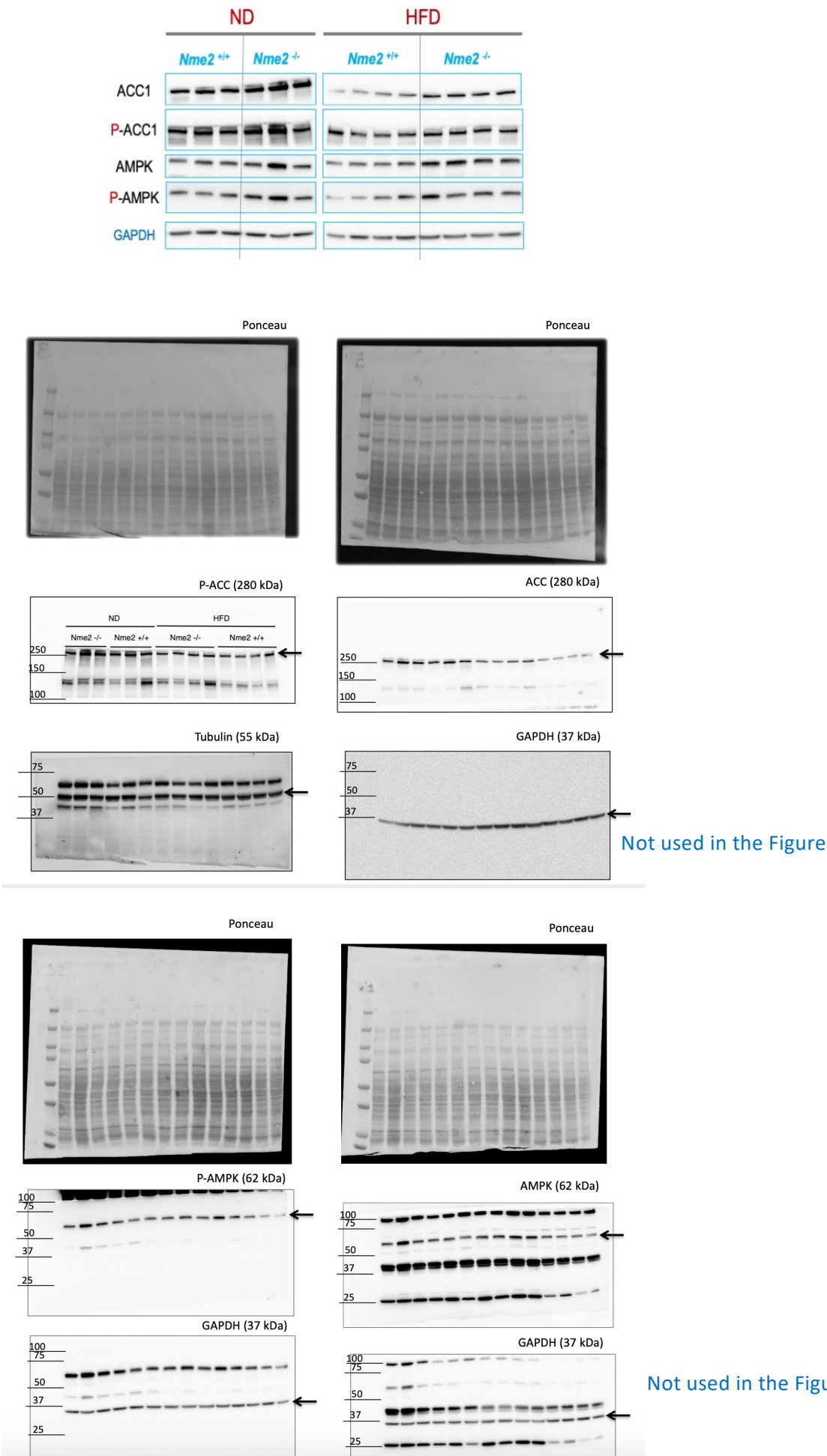

Supplement: Supplementary file 3 — Data S1 and S2 [file sciadv.adh0140_data_s1_and_s2.zip › adh0140_Data_S1.pdf]
